# Evaluating the Species Boundaries of Green Microalgae (*Coccomyxa*, Trebouxiophyceae, Chlorophyta) Using Integrative Taxonomy and DNA Barcoding with Further Implications for the Species Identification in Environmental Samples

**DOI:** 10.1371/journal.pone.0127838

**Published:** 2015-06-16

**Authors:** Tatyana Darienko, Lydia Gustavs, Anja Eggert, Wiebke Wolf, Thomas Pröschold

**Affiliations:** 1 M. G. Kholodny Institute of Botany, National Academy Science of Ukraine, Kyiv 01601, Ukraine; 2 University of Rostock, Institute of Biological Sciences, Applied Ecology and Phycology, Albert-Einstein-Strasse 3, D-18059 Rostock, Germany; 3 Leibniz Institute for Baltic Sea Research Warnemünde, Physical Oceanography, D-18119 Rostock, Germany; 4 University of Vienna, Department of Limnology and Biooceanography, A-1090 Vienna, Austria; Consiglio Nazionale delle Ricerche (CNR), ITALY

## Abstract

Integrative taxonomy is an approach for defining species and genera by taking phylogenetic, morphological, physiological, and ecological data into account. This approach is appropriate for microalgae, where morphological convergence and high levels of morphological plasticity complicate the application of the traditional classification. Although DNA barcode markers are well-established for animals, fungi, and higher plants, there is an ongoing discussion about suitable markers for microalgae and protists because these organisms are genetically more diverse compared to the former groups. To solve these problems, we assess the usage of a polyphasic approach combining phenotypic and genetic parameters for species and generic characterization. The application of barcode markers for database queries further allows conclusions about the ‘coverage’ of culture-based approaches in biodiversity studies and integrates additional aspects into modern taxonomic concepts. Although the culture-dependent approach revealed three new lineages, which are described as new species in this paper, the culture-independent analyses discovered additional putative new species. We evaluated three barcode markers (V4, V9 and ITS-2 regions, nuclear ribosomal operon) and studied the morphological and physiological plasticity of *Coccomyxa*, which became a model organism because its whole genome sequence has been published. In addition, several biotechnological patents have been registered for *Coccomyxa*. *Coccomyxa* representatives are distributed worldwide, are free-living or in symbioses, and colonize terrestrial and aquatic habitats. We investigated more than 40 strains and reviewed the biodiversity and biogeographical distribution of *Coccomyxa* species using DNA barcoding. The genus *Coccomyxa* formed a monophyletic group within the Trebouxiophyceae separated into seven independent phylogenetic lineages representing species. Summarizing, the combination of different characteristics in an integrative approach helps to evaluate environmental data and clearly identifies microalgae at generic and species levels.

## Introduction

Microalgae and protozoa (protists) are traditionally classified according to morphological and cytological characteristics of vegetative stages in their life cycle. Phylogenetic analyses of ribosomal genes (SSU and ITS rDNA sequences) have demonstrated that this morphological concept is artificial for most of the microalgal genera and should be revised [1, 2]. Several studies have shown that the morphology could be dependent on environmental factors and can vary under different environmental conditions, which led to uncertain identification at the species level, especially in investigations of natural samples using light microscopy [3–5]. For example, green algae have been divided into two major groups according to their reproductive mechanism: sexual or asexual species. In species that exhibit sexual reproduction, crossing experiments can be conducted to test the biological species concept and identify organisms at the species level, but the situation for asexual species is more complex. Organisms such as coccoid green microalgae often lack characteristic morphological features and therefore cannot be classified at a higher taxonomic level without additional data. For example based on SSU rDNA sequences, species of the genus *Chlorella* (in traditional morphological sense) occur in different lineages of the Chlorophyceae and Trebouxiophyceae (see details in [4] and references therein). Summarizing, two scenarios concerning morphological characteristics based on phylogenetic studies exist: (i) morphologically similar species belong to different phylogenetic lineages or (ii) species of different shapes and morphology are closely related and form a monophyletic lineage. Morphological characteristics, commonly used for the traditional classification of algae, need to be evaluated for each genus and species before being used as diagnostic characteristics in taxonomy. However, because these morphological evaluations are very time consuming and require experience, the molecular species concept seems to be the only method to distinguish organisms and define species and genera in a short period ([4] and references therein). The ‘molecular revolution’ resulted in an ongoing active discussion about the appropriate scale for species delimitation for a functional, meaningful taxonomy. Further difficulties arise from the nomenclatural rules for naming species. The International Code of Nomenclature for Algae, Fungi and Plants (ICN; Melbourne Code, [6]) solely regulates the naming process of species without an evaluation of characteristics leading to the species identification. However, as previously mentioned this evaluation process for assigning a scientifically correct name to a species is often difficult and time consuming. The latest version of ICN allows the depositing of DNA and cultured material (in a metabolic inactive state) as type material for a species name, but it is not commonly used. Taxonomic revision using this approach is impossible for large scale environmental studies and has been recently replaced by DNA barcoding. The International Barcoding of Life Initiative (IBOL; www.ibol.org) aims to find a single, universal, short DNA fragment that is easy to sequence and leads to a clear species identification. Zoologists and mycologists established the mitochondrial cytochrome oxidase subunit 1 (*cox 1* or COI) and nuclear internal transcribed spacer region of the ribosomal operon (ITS) respectively as general barcode markers [7–9]. A combination of two plastid markers (*rbcL* and *matK*) has been proposed as DNA barcode markers for land plants [10]. In contrast, a general marker for microalgae and protists has not been proposed because of the high diversity in different genes among those organisms resulting in different markers for each group. As a pre-barcode marker two variable regions of the SSU rDNA sequences called the V4 [11, 12] and V9 [13] regions have been suggested. The internal transcribed spacer region 2 (ITS-2) is often used for phylogenetic studies of microalgae at species level, but it has not been proposed as a barcode marker because of the difficulties with the alignment of sequences and the prediction of secondary structure. However, ITS-2 and its corresponding secondary structure provides a unique possibility to predict the mating ability of sexual organism; therefore, it is very useful for the discrimination of biological species [14, 15]. Considering the presence of compensatory base changes (CBCs) in the secondary structures of ITS rDNA sequences, the genus and species can be defined and show additional genetic signatures [16]. Interestingly, if two specimens differ in at least one CBC in the conserved region of ITS-2, they cannot mate [15]. Demchenko et al. [17] have used this region of ITS-2 as barcode marker for the green algal genus *Microglena* to support the species delimitation of this genus. The usage of ITS-2 as barcode marker among protists and microalgae, especially in asexual species is still unused at a larger scale and must be validated.

In a taxonomic context, DNA sequence information without other corroborating evidence can never be used by itself as an indicator for species delimitation [18]. Furthermore, molecular phylogenetic studies often reveal cryptic species, which are also discussed as operational taxonomical units (OTUs). It is not clearly defined whether the detected sequence differences could result in the delimitation of different species. One explanation is that lineage sorting is in progress and has not been completed yet. However, it is also possible that the detected differences are meaningless for taxonomic purposes, and the resolution of genetic differences has been considerably too detailed. A reasonable approach to a meaningful identification of species and taxa at all levels must be connected to a specimen (a voucher in a herbarium or a culture in a collection) of the target organism. A consequence of that uncertainty is that sequence databases are filled with entries that can be assigned to neither a morphotype nor a species name (e.g. ‘environmental sample’) or an ecological context (e.g. ‘unidentified Trebouxiophyceae’).

In this study, we demonstrate for the first time a compromise using an integrative approach for characterization of cultured green algal material, and their distribution pattern in different habitats to evaluate culture-dependent and culture-independent methods. We used the model organism *Coccomyxa subellipsoidea* and its close relatives for this study, which is the first terrestrial green alga for which the genome was completely sequenced [19].

The genus *Coccomyxa* belongs to the green algal class Trebouxiophyceae (sister to the Chlorophyceae and Ulvophyceae), which can be subdivided by molecular approaches into the *Chlorella*-, *Oocystis-* and *Trebouxia-* lineages (*sensu* [20]), the latter consisting of mostly terrestrial species. So far all known members of *Coccomyxa* belong to the *Elliptochloris*-clade [19, 21]. Representatives of the genus *Coccomyxa* occur as free-living in terrestrial green biofilms, as soil algae and associated with mosses [22–25] as well as planktonic in limnic ecosystems [26–27]. Further, this genus participates in symbiotic associations with asco- and basidiomycetes [28–30], with higher plants [31] and is able to infest marine mussels [32, 33]. *Coccomyxa* even occurs as a contaminant in chemical solutions or distilled water in laboratories [34] or in the cooling water of a nuclear power plant [35]. However, no evidence has been published about the distribution of *Coccomyxa* free-living in marine environment. *Coccomyxa* has a worldwide distribution, can also form biofilms, can be dominant in certain ecosystems and displays a remarkable versatility in habitat and lifestyle [5]. Representatives of this genus are characterized by a small size (6–14 x 3–6 μm), an irregular elliptical to globular cell shape, a parietal chloroplast shape without a pyrenoid and the absence of any flagellated stages [36]. *Coccomyxa*-like organisms have been subdivided into three genera according to their morphology. Fott [37] transferred all species of *Coccomyxa* lacking mucilage to *Choricystis* and all species with a one-sided mucilage cap (previously described by Jaag) to the genus *Pseudococcomyxa*. Therefore, only species with huge, partly layered mucilage remained in the genus *Coccomyxa* [24]. Pröschold et al. [21] have shown that *Choricystis* represents its own phylogenetic lineage among the Trebouxiophyceae, but whether *Coccomyxa* and *Pseudococcomyxa* are two different genera is an open question because only iconotypes of the type species of both genera exist and no culture material is available. Fortunately, most strains investigated by Jaag [36] are available in public culture collections, and some are even the authentic material of described species.

The aim of this study is to discover and characterize the variability of *Coccomyxa* using an integrative approach with further implications for biodiversity investigations using culture-dependent and culture-independent methods for research on microalgae and protists. To demonstrate this approach, we focussed on the following questions: (i) How we can detect species boundaries in those asexual microorganisms? (ii) Can we find a suitable molecular barcode marker to discriminate species? (iii) Do *Coccomyxa*-like algae form a monophyletic group and how many species can be distinguished within this genus? (iv) Is the missing occurrence in marine habitats caused by highly sensitive physiological response to enhanced salinity? (v) Does the biodiversity of *Coccomyxa* in culture represent its genetic diversity in nature, and are there any geographical distribution pattern, or ecological and lifestyle preferences?

**Fig 1:**
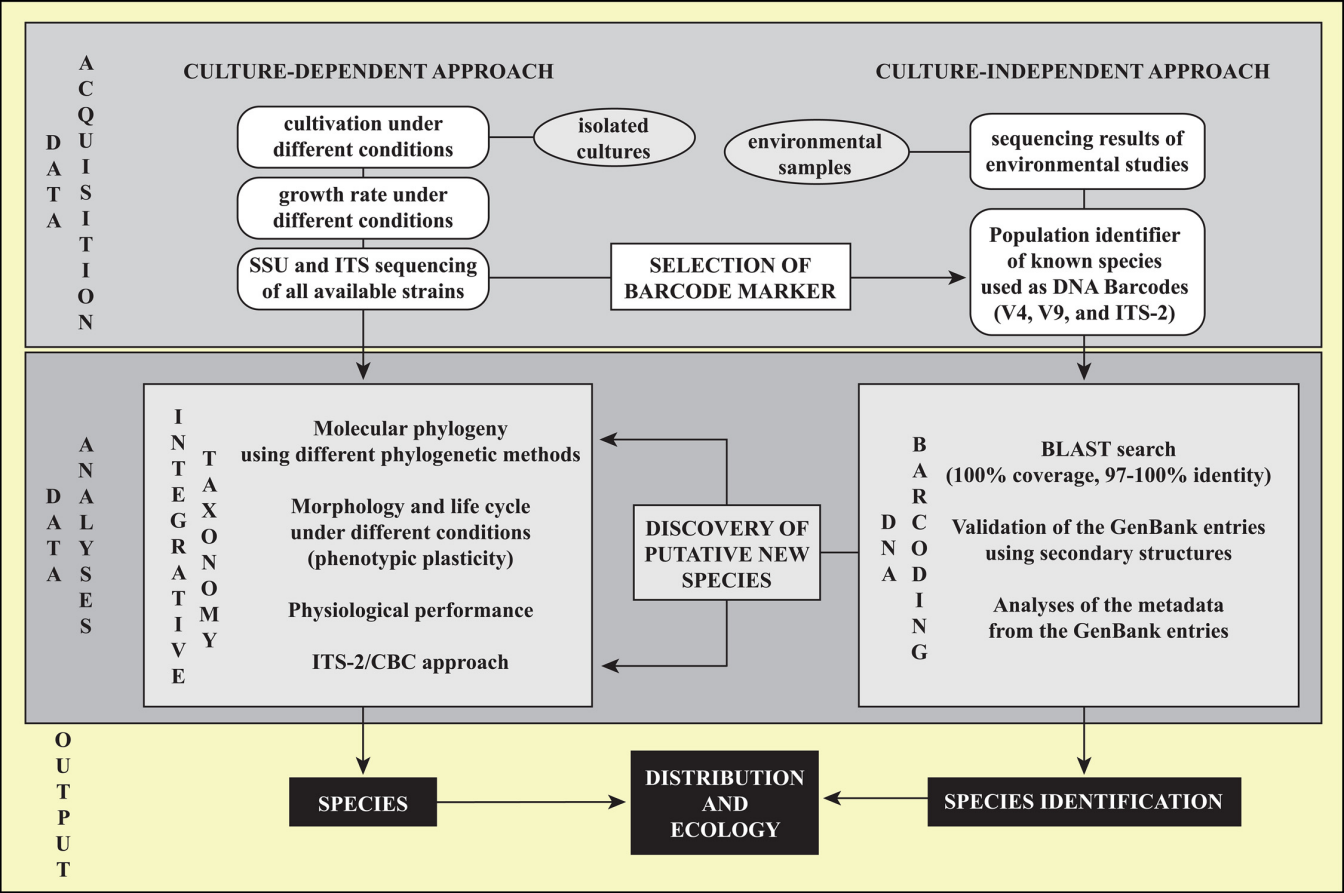
Work scheme in the present study.

To answer these questions, we studied all available strains of *Coccomyxa* (including the strain NIES 2166; genome sequence) using an integrative approach (phylogenetic analyses of the nuclear SSU and ITS rDNA based on their secondary structures, phenotypic and physiological plasticity). Three barcode marker regions based on our sequences (V4, V9 and ITS-2) have been evaluated and used to uncover the distribution pattern of the species discriminated in this study by a BLAST search algorithm. The working scheme is summarized in Fig 1.

## Material and Methods

### Strains, cultivation and morphological characterization

The 41 strains used in this study were obtained from public and working culture collections (details in S1 Table). All algal cultures were grown in modified Bold´s Basal Medium (3N-BBM+V; medium 26a in [38]) with one exception of SAG 49.84, which only grew in 3N-BBM+V with 1% (w/v) glucose added. For the morphological observations, the strains were cultivated on liquid 3N-BBM+V under standard laboratory conditions and a light: dark cycle of 16:8 hrs at +20 ±1°C and 35 μmol photons/m^2^s^1^ provided by daylight fluorescent tubes (Osram L 36W/954 Lumilux de lux daylight, Munich, Germany). To induce mucilage formation, which is regarded as a morphological feature useful for species identification, starvation experiments were conducted according to Vischer [39]. 3N-BBM+V medium was modified by addition of 1% (w/v) glucose and finally diluted with distilled water (1:20 v/v). The species were identified using the identification keys of Ettl and Gärtner [5] and Komárek and Fott [24] and compared with original descriptions of the species published by Schmidle [22], Acton [40], Chodat [41], Jaag [36], and Jaag and Thomas [42]. For the light microscopic investigations, an Olympus BX-51 was used (Olympus, Tokyo, Japan). The micrographs were taken with a CCD ColourView-II camera (Soft Imaging System, Münster, Germany) using the analySIS imaging system (Soft Imaging System, Münster, Germany).

### DNA isolation, PCR and sequencing

Genomic DNA was extracted from all strains (including two additional strains, which were used as an outgroup in the phylogenetic analyses: SAG 245.80 *Elliptochloris bilobata* and SAG 62.90 *Hemichloris antarctica*) using the DNeasy Plant Mini Kit (Qiagen, Hilden, Germany) following the instructions provided by the manufacturer. The SSU and ITS rDNA were amplified in PCR reactions using the Taq PCR Mastermix Kit (Qiagen, Hilden, Germany) with the primers EAF3 and ITS055R [43]. The PCR products were purified and sequenced as described by Demchenko et al. [17]. The nucleotide sequences are available in the EMBL, GenBank and DDBJ sequence databases under the accession numbers given in S1 Table.

### Phylogenetic analyses

The SSU rDNA sequences of all strains (including outgroup) were aligned according to their secondary structure by comparison of the structure presented for the strain NIES 2166 *Coccomyxa subellipsoidea* (S1 Fig). The sister taxa to *Coccomyxa*, *Elliptochloris bilobata* and *Hemichloris antarctica* were chosen as the outgroup [21]. The ITS-1 and ITS-2 sequences of all strains were folded by using the program mfold ([44, 45]; http://mfold.rna.albany.edu/?q=mfold/RNA-Folding-Form), and their ITS-2 structures are summarized in S2 Fig, produced using the programs PseudoViewer3 [46], Adobe Illustrator CS5.1 (Adobe, San Jose, California), and Pages’09 4.3 (Apple, Cupertino, California). A consensus ITS-2 secondary structure was created from all these structures using the program 4SALE version 1.7 [47] and then manually modified using Adobe Illustrator. Considering these structures, ITS-1 and ITS-2 were separately aligned manually and with the program MARNA ([48]; http://rna.informatik.uni-freiburg.de/MARNA/Input.jsp) to avoid any bias in the alignments. The resulting alignments were included in a concatenated dataset (2671 bp) of SSU (1784 bp), ITS-1 (402 bp), 5.8S (158 bp), ITS-2 (308 bp) and LSU (19 bp) rDNA sequences. The alignments for phylogenetic and secondary structure analyses are available as NEXUS and FASTA files in the Supplemental Material, respectively (S1 Datasets).

**Fig 2:**
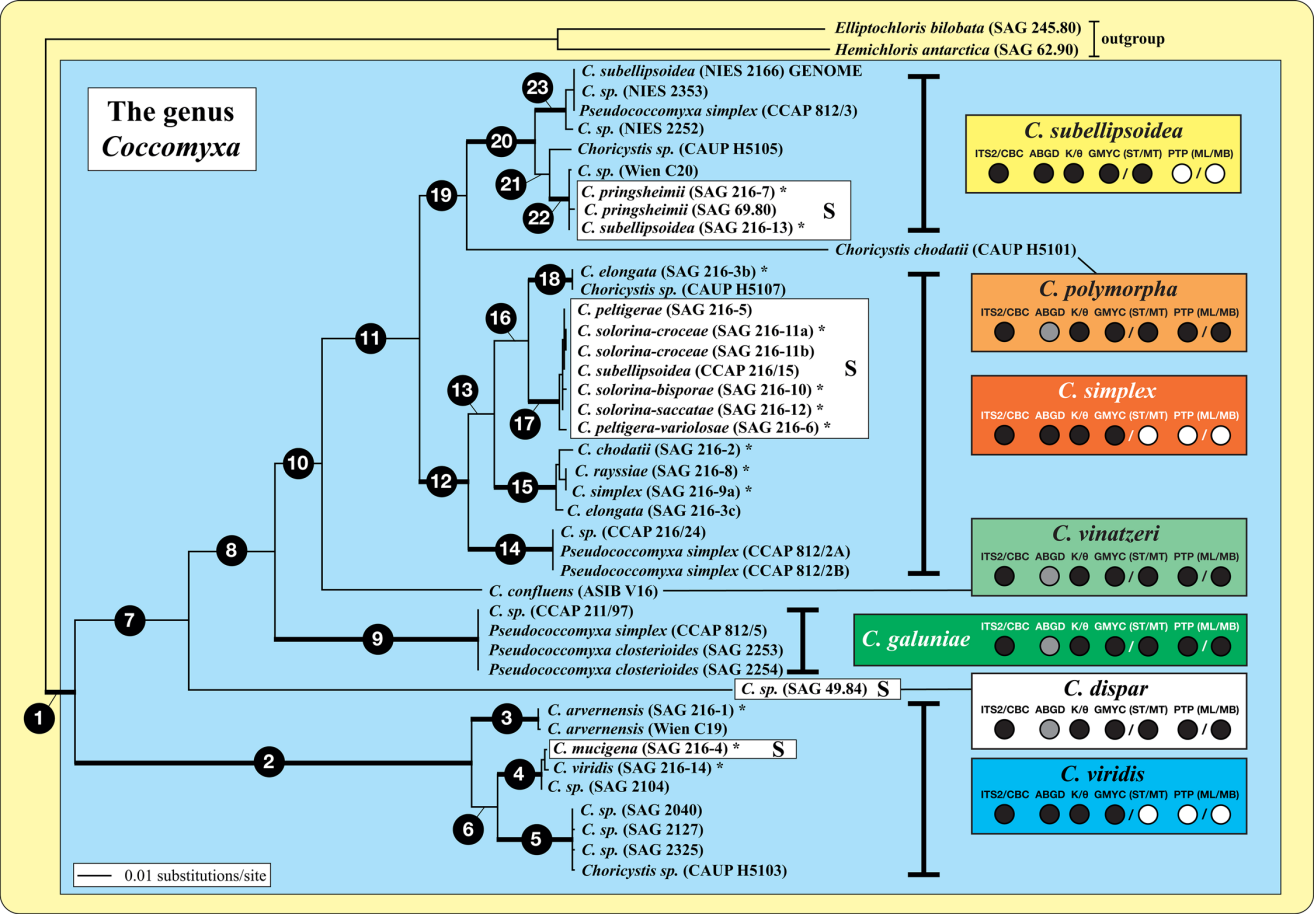
Molecular phylogeny of the *Elliptochloris*-clade (Trebouxiophyceae) based on SSU and ITS rDNA sequence comparisons. The phylogenetic tree shown was inferred using the maximum likelihood method based on a concatenated data set of 2671 aligned positions of 43 taxa using PAUP 4.0b10. For the analysis, the GTR+I+G model (base frequencies: A 0.2372, C 0.2575, G 0.2781, T 0.2272; rate matrix: A-C 1.3929, A-G 1.8871, A-T 1.0278, C-G 0.6574, C-T 3.7034, G-T 1.0000) with the proportion of invariable sites (I = 0.5745) and gamma distribution shape parameter (G = 0.3685) was chosen, which was calculated as the best model by Modeltest 3.7. Bayesian and bootstrap values of the branches 1–23 were calculated from the concatenated data set as well as from both genes separately using different methods, as described in Material and Methods, and these values are shown in S2 Table. The branches in bold are highly supported (Bayesian values > 0.95; bootstrap values > 70%) in all analyses. The sister group of *Coccomyxa* (*Elliptochloris bilobata* and *Hemichloris antarctica*) was chosen as an outgroup. Names at the branch are the original designation of the strains; names in the boxes were assigned after revision using the integrative approach. The circles in the boxes showed the results of different species delimitation approaches (distance-based methods: ABGD and K/θ; phylogeny-based methods: GMYC (ST = single threshold, MT = multi threshold) and PTP (ML = maximum likelihood, MB = Bayesian); see details in Material and Methods) compared to the ITS2/CBC approach presented in this study (black circles = species delimitation supported, grey circles = single taxa needed to be excluded before analyzing with ABGD, white circles = species delimitation not resolved). Authentic strains (‘type cultures’) are marked with an asterisk. S highlighted the photobionts of lichens.

To determine the evolutionary model that fit the dataset best (concatenated and separated according to their genes) the program Modeltest 3.7 [49] was used. Considering the results of these tests, the best models were selected by the Akaike Information Criterion [50]. For datasets, concatenated or partitioned according to their genes, the GTR model with a proportion of invariable sites (I) and a gamma shape parameter (G) was used for the phylogenetic analyses. The phylogenetic tree (Fig 2) was inferred by distance (neighbor-joining [NJ] using the GTR+I+G model), maximum parsimony (MP), and maximum likelihood (ML; using GTR+I+G) criteria using PAUP version 4.0b10 [51], by randomized accelerated maximum likelihood using RAxML version 7.0.3 [52], and by Bayesian inference (BI) using MrBayes version 3.1 [53, 54] and the PHASE package 2.0 [55–59]. The RAxML analyses of the concatenated dataset were performed partitioned according to their genes. For PHASE analyses, four models were used according to their secondary structures and to their genes; the REV model [60] was used for the unpaired regions and the RNA7D model [61] was used for the paired regions. The Bayesian and bootstrap support for each branch in Fig 2 are summarized in S2 Table. The tree topology presented in Fig 2 was compared with user-defined trees that were manually generated with the program TreeView 1.6.6 [62]. The log-likelihood values of these tree topologies was calculated using PAUP and compared via approximately unbiased (AU), Kishino-Hasegawa (KH), and Shimodaira-Hasegawa (SH) tests using CONSEL version 0.1i [63–64]. The results of these tests are summarized in S3 Table.

### ITS2/CBC approach and species delimitation methods

**Fig 3:**
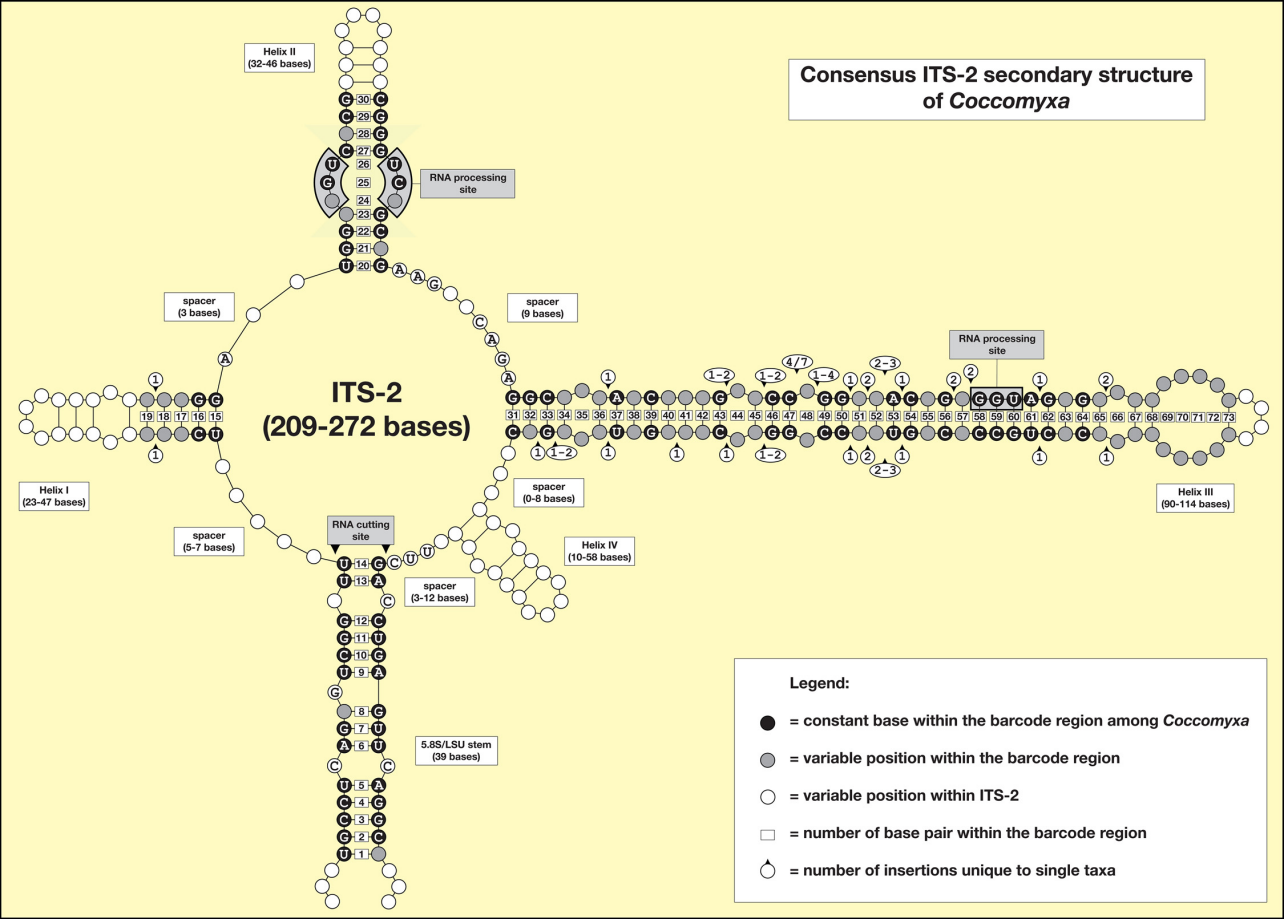
Consensus ITS-2 secondary structure of *Coccomyxa* based on comparison of the structures of each strain (see S2 Fig). The 73 base-pairs used for the ITS-2 DNA Barcode are numbered and marked in black and grey circles.

**Fig 4:**
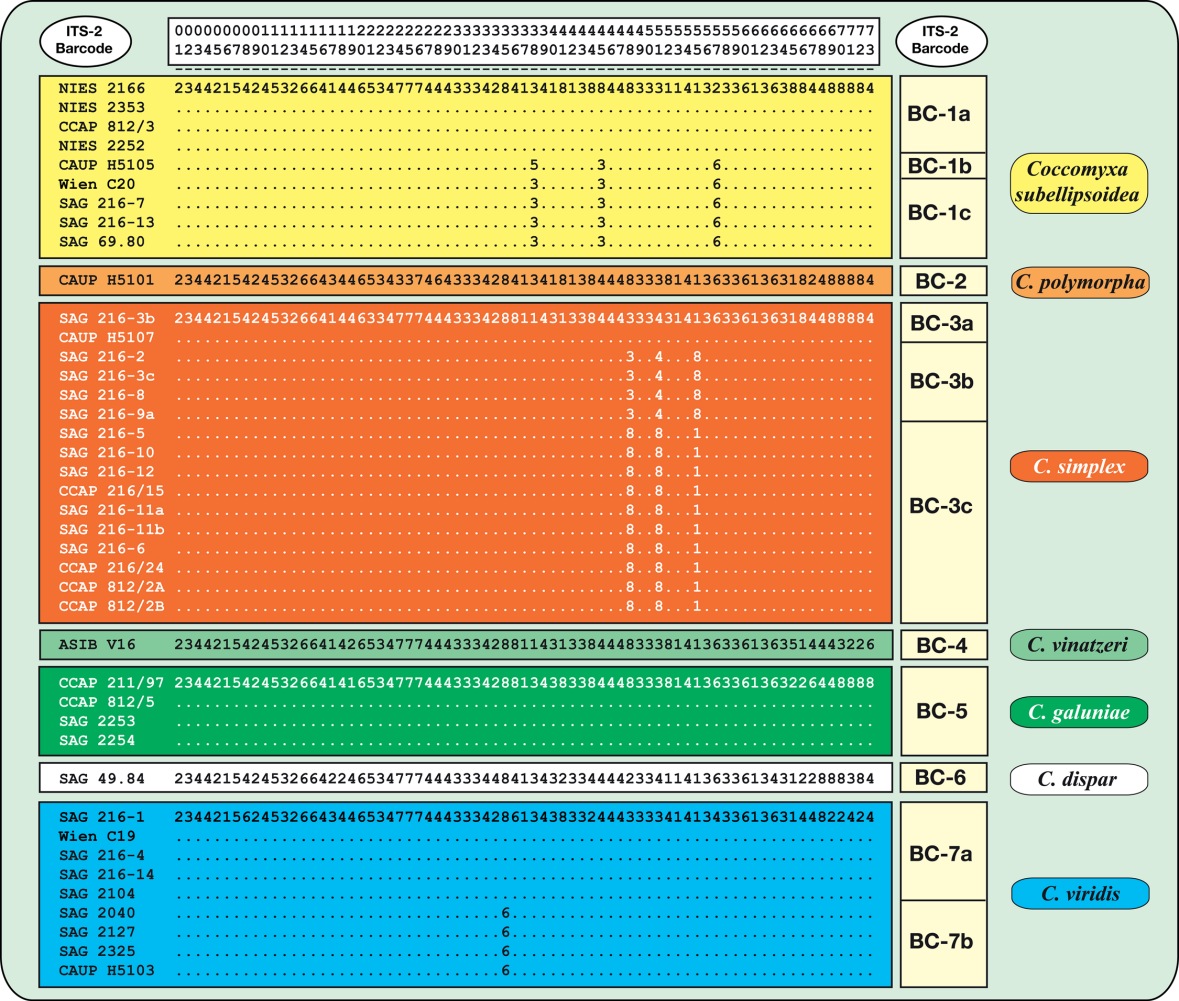
ITS-2 DNA Barcode of the *Coccomyxa* species. The aligned conserved region of ITS-2 was translated into a base-pair alignment using a number coding for each base-pair (**1** = A-U, **2** = U-A, **3** = G-C, **4** = C-G, **5** = G•U, **6** = U•G, **7** = mismatch, **8** = deletion, unpaired or single bases) and is given for each strain in S2 Fig. Equal base-pairs within species are marked with a dot. The ITS-2 barcodes were designated by numbers (variants within species were named by lowercase letters) for each species.

To find molecular signatures that can be used as DNA barcodes for the identification of *Coccomyxa* species, the conserved region of ITS-2 was extracted manually from the alignment created by MARNA. According to the recommendation of Coleman [15], the conserved region (highlighted in numbered boxes in Fig 3; nos. 1–73), using the 14 bp of the 5.8S-LSU stem, the first 5 bp of Helix I, the first 11 bp of Helix II (including the pyrimidine-pyrimidine mismatch), and all base-pairs of Helix III were selected for the DNA barcode (highlighted in blue for each strain in S2 Fig). The resulting sequences were manually aligned again and proven automatically using MARNA. This alignment was used for finding compensatory base changes (CBCs; see S4 Table) using the program 4SALE version 1.7 [47]. This sequence alignment was translated into the base-pair alignment by replacing each base-pair by a number (A–U = 1; U–A = 2, G–C = 3, C–G = 4, G•U = 5, U•G = 6, mismatch = 7, deletion or unpaired or single bases = 8; Fig 4). In addition, a reduced SSU and ITS dataset (the taxa were represented by only one to four sequences that were removed from the complete dataset) was analyzed using the program ABGD [65] to identify barcoding gaps among the species. For another distance-based analysis (K/θ; [66]), the uncorrected p-distances were calculated by PAUP and compared following the instruction provided in Birky et al. 2010 [66]. The results of these analyses were compared with those of phylogeny-based methods, which were proposed in the guideline of Fontaneto et al. 2015 [67]. For these analyses, the SSU and ITS data set was modified according to the instructions for each method. Identical sequences and sequences of both outgroup taxa were excluded for GMYC and PTP analyses. For GMYC [68], an ultrametric tree was calculated with BEAST 1.8.0 [69] using the HKY model and a strict clock (MCMC: 10,000,000 generations; burnin 1,000,000). The consensus of the sampled trees was built with TreeAnnotator 1.8.0 of the BEAST package. The consensus tree was used as input file for the GMYC analyses (http://species.h-its.org/gmyc/) using both methods (single and multi threshold). For PTP [70], a maximum likelihood of the reduced GMYC data set was calculated by RAxML version 7.0.3 [52] and the resulting best tree was used as input file for analysis (http://species.h-its.org/gmyc/) with 500,000 MCMC generations. The results of all these analyses are summarized in Fig 2.

### Salinity dependent growth response of *Coccomyxa*

To investigate and evaluate the physiological variability within *Coccomyxa*, we compared the growth rates of *Coccomyxa* species as affected by salinity. The cultures were inoculated in 3N-BBM+V adjusted to different salinities by the addition of various amounts NaCl to achieve final concentrations of 0.2 (3N-BBM+V), 0.6, 1.2, 1.8, 2.5 and 3.3%. For SAG 49.84, 1% (w/v) glucose was added for each salinity.

These experiments were performed in two steps: (i) Pre-cultures were inoculated in 6-well culturing microplates (Greiner Bio-One, Frickenhausen, Germany) for up to 10 days under the respective culture conditions to ensure complete adaptation of the algal cultures. These cultures were investigated by light microscopy; the length and width of ~ 100 cells of each strain was measured, and the length: width ratio was calculated (S3 Fig). (ii) The pre-cultures served as the inocula for the growth experiments, which were performed in 24-well culturing microplates (Greiner Bio-One, Frickenhausen, Germany) with 4 replicates per approach. Fresh medium (1 ml) was mixed with 100 μl of pre-culture to achieve a cell concentration of 0.1–0.2*10^6^ cells ml^-1^. The microplates were sealed with parafilm, and measurements were performed every 24 hrs for up to 10 days. All growth experiments were conducted under standard conditions of 20°C, 50 μmol m^-2^s^-1^ with a light:dark cycle of 16:8 hrs.

Growth measurements were performed using the Spectra Max M2^e^ microplate reader (Molecular Devices, Sunnyvale, CA, USA) by monitoring the increase of Chlorophyll *a* (chl-a) fluorescence over time as an indicator of microalgal growth according to Kitzing et al. [71]. Chl-a was excited at 440 nm, and the fluorescence emission was detected at 680 nm with 21 points per well from the bottom-up. To avoid a false positive detection at 680 nm, a cut-off wavelength of 665 nm was set for the measurements. Chlorophyll fluorescence was measured every 24 hrs for up to 10 days. To evaluate the measuring range of the applied technique, a geometric serial dilution with six steps was prepared from a log-phase culture of *C*. *subellipsoidea* NIES 2166 in three replicates. In addition to chl-a fluorescence, we measured the traditional biomass markers cell count (by a haemocytometer) and chl-a concentration (via ethanolic extraction and photometric quantification; modified after Gustavs et al. [72]) for every dilution step. The correlation between fluorescence and the traditional biomass markers was linear for cell counts from 0.2 to 3.1*10^6^ cells ml^-1^ and chl-a concentrations from 0.06 to 0.53 mg l^-1^, respectively (data not shown). Maximum growth rates in the exponential phase were calculated for each of the four replicates independently by fitting the exponential growth curve with an iterative optimization procedure [72].

Salinity-dependent growth rates were calculated as median values of four replicates and the factor ‘salinity sensitivity’ results from the decline of maximal growth rate from 0.2 and 0.6% NaCl, respectively, to the pessimal growth condition at 1.8, 2.5 and 3.3%, respectively. The dataset was referenced to the most sensitive strain CAUP H5101, resulting in the highest sensitivity value for the respective strain and low sensitivity values for robust strains, e.g., SAG 2104 (S5 Table).

### Comparison of different barcode markers using BLAST N search algorithm for identification of *Coccomyxa* species in environmental sequencing

To evaluate whether the strains sequenced in this study represented the biodiversity of *Coccomyxa* in nature, we extracted three barcode regions from our sequences and compared those with the entries in GenBank using the BLAST N search algorithm (default settings; [73]). We used the V4 (Pos. 616–845 in our alignment) and V9 (Pos. 1631–1737) regions of the SSU rDNA sequences as well as the first three helices including the spacer regions of ITS-2 for this comparison the flanking 5.8S/LSU stem and Helix IV of ITS-2 were excluded for the BLAST search because many GenBank entries were incomplete and would result in fewer *Coccomyxa* records in GenBank). To detect the variability within the V4 and V9 regions, we analyzed these regions according to their secondary structures (the V4 and V9 structure are demonstrated in S4 Fig for NIES 2166 *Coccomyxa subellipsoidea*) and detected the CBCs and Hemi-CBCs (HCBCs) among the species of *Coccomyxa* (see S4 Table).

The BLAST N search was conducted using the following criteria: the extracted V4, V9, and ITS-2 regions of our strains (designated as the population identifier V4-A1-G, V9-A-F2, and ITS2-A1-G5) were used for the BLAST search. The resulting hits with 100% coverage and 97–100% identity are summarized in S6 Table. Sequences identical to those generated in this study are marked with the corresponding population identifier (marked in red) of the species in S6 Table; entries different in at least one or more bases to ours are highlighted with a number in brackets in S6 Table and have been checked according to the secondary structures. The results of these comparisons are summarized in S7 Table. To demonstrate the distribution pattern of the *Coccomyxa* species, the metadata of all GenBank entries and its related publications if available found with our BLAST search were analyzed and summarized in S7 Table.

### Nomenclature

The electronic version of this article in Portable Document Format (PDF) in a work with an ISSN or ISBN will represent a published work according to the International Code of Nomenclature for algae, fungi, and plants; hence, the new names contained in the electronic publication of a PLOS ONE article are effectively published under that Code from the electronic edition alone, so there is no longer any need to provide printed copies.

## Results

### Molecular phylogeny of *Coccomyxa*

Together with *Elliptochloris* and *Hemichloris*, the genus *Coccomyxa* forms a monophyletic group within the green algal class Trebouxiophyceae (*Elliptochloris*-clade) and is the sister group to the former two genera ([21, 19]; see also S5 Fig). Phylogenetic analyses of a concatenated dataset (SSU and ITS rDNA sequences) showed that 41 strains previously assigned as *Coccomyxa*, *Pseudococcomyxa*, and *Choricystis* belong to the *Elliptochloris*-clade (Fig 2). The strains investigated are subdivided into seven lineages within *Coccomyxa*, designated as *C*. *subellipsoidea*, *C*. *simplex*, *C*. *dispar*, *C*. *viridis*, and three new lineages called *C*. *polymorpha*, *C*. *vinatzeri*, and *C*. *galuniae* (newly described as species, below). Many of those strains are authentic strains (= ‘type cultures’; marked with an asterisk in Fig 2) of described species (see [36]). The photobionts of lichens (highlighted in boxes with **S** in Fig 2) are not monophyletic within *Coccomyxa* and are related to free-living isolates of *C*. *subellipsoidea*, *C*. *simplex*, and *C*. *viridis*. The only available strain identified as *C*. *dispar* (SAG 49.84) was isolated from the basidiolichen *Multiclavula vernalis* and represented a lineage of its own. The branches highlighted in bold in Fig 2 are highly supported in all Bayesian (PHASE and MrBayes) and bootstrap analyses (likelihood, distance and parsimony) using the concatenated dataset; however; the support is lower if SSU and ITS datasets are analyzed separately. The support for all the numbered branches in Fig 2 (1–23) is summarized in S2 Table.

### ITS-2/CBC approach to define species in *Coccomyxa*

To decide which branches in Fig 2 should be considered species, we analyzed the conserved region of ITS-2 for discriminating species using the approach previously described by Demchenko et al. [17]. This approach used the conserved region of ITS-2 (5.8S/LSU stem, the stems of Helix I and II, and all paired bases of Helix III). The procedure is described in detail in Material and Methods and demonstrated as a consensus in Fig 3 and S2 Fig for each strain. Considering these analyses twelve different haplotypes (BC1a-7b) were discovered among *Coccomyxa* (Fig 4). The CBC/HCBC analysis (S4 Table) revealed that each of the seven species is supported by several CBCs and HCBCs. Strains belonging to *C*. *subellipsoidea*, *C*. *simplex*, and *C*. *viridis* represented different haplotypes, but those do not differ in CBCs and show differences only because of missing base pairs (Fig 4). Summarizing, the seven lineages representing species in Fig 2 could be clearly differentiated using the ITS-2 approach demonstrated here, but the subdivisions within *C*. *subellipsoidea* (branches 21–23), *C*. *simplex* (14–18) and *C*. *viridis* (3–5) are not supported by any CBCs/HCBCs. The genetic variability among the ITS-2 sequences of these species were as follows: *C*. *subellipsoidea*: 3.6% (= 13 variable bases), *C*. *simplex*: 11.4% (= 41), and *C*. *viridis*: 13.0% (= 54). To decide the location of the species boundaries, the evolutionary distances within these three species were calculated and compared using the program ABGD. No gap within the species could be observed, but the species are clearly separated by a gap (see Fig 2). The species delimitation was confirmed by the K/θ and GMYC (single threshold), but *C*. *simplex*, *C*. *subellipsoidea*, and *C*. *viridis* could not be resolved using GMYC (multi threshold) and PTP (Fig 2). In addition, the monophyly of these three species demonstrated using the high Bayesian and bootstrap support of the branches 2, 12, and 20 in Fig 2 was confirmed by testing user-defined tree topologies (collapsed branches of those branches) with several statistical methods by CONSEL. All user-defined trees were significantly worse (P<0.05) and therefore rejected by approximately unbiased test (S3 Table).

### Morphological identification and plasticity

**Fig 5:**
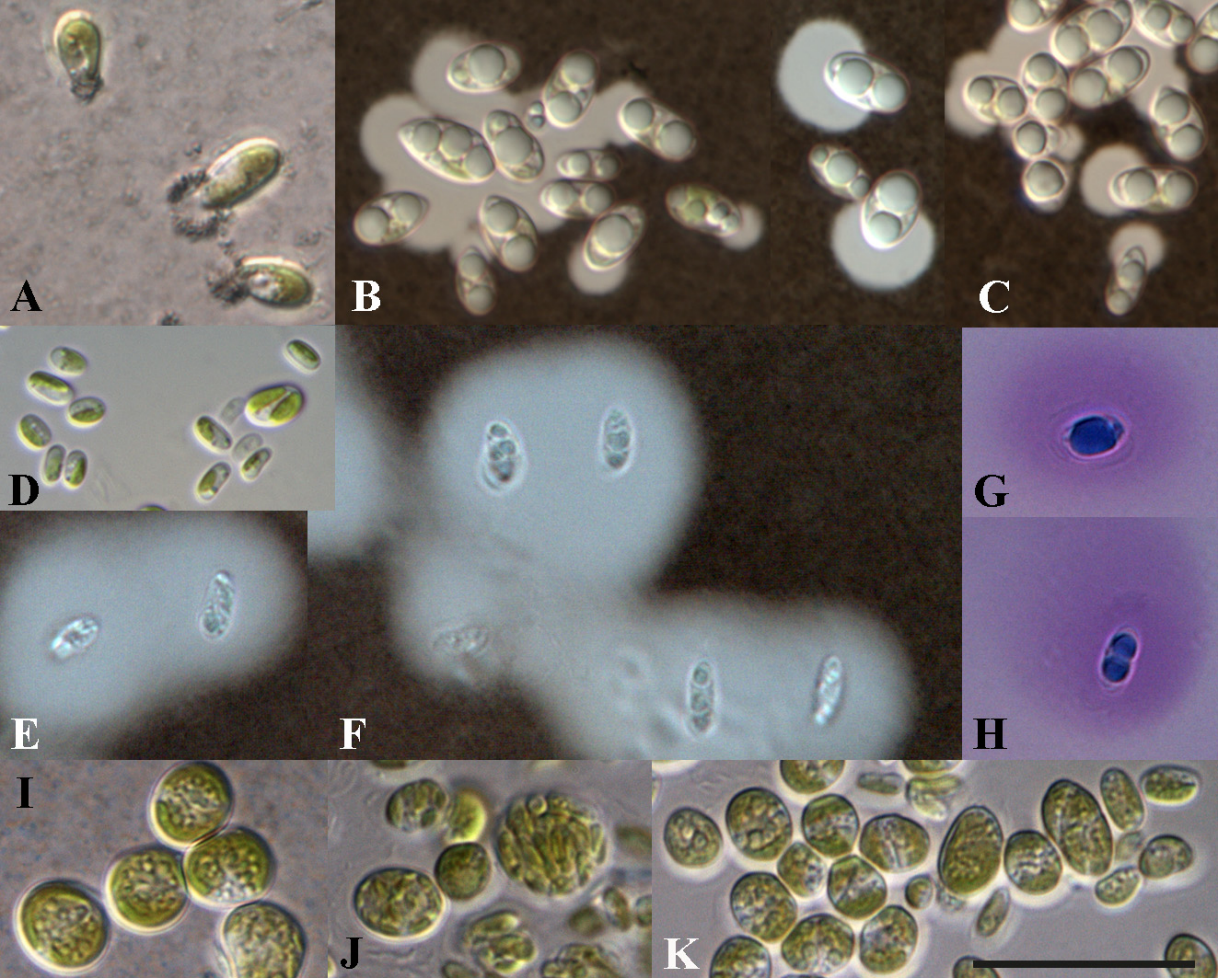
Mucilage production of *Coccomyxa dispar* and *C*. *viridis* cultivated in 1:20 diluted 3N-BBM+V containing 1% glucose. A-C, *C*. *dispar* SAG 49.84; D-K, *C*. *viridis* SAG 216–4; B, C, E and F ink staining; G, H staining with methylene blue. Scale bar = 10 μm.

The 41 strains originally designated as *Coccomyxa*, *Pseudococcomyxa*, and *Choricystis* have been cultivated under standard conditions as described above and investigated after two weeks of growth using light microscopy. All strains could be clearly identified as members of the genus *Coccomyxa* (origin of the strains are summarized in S1 Table). The morphology of 13 authentic strains representing species previously described by Jaag [36] corresponded with their original diagnoses. The other remaining strains could be either identified as *C*. *subellipsoidea*, *C*. *simplex*, or *C*. *arvernensis* using traditional identification keys. However, the authentic strain of *C*. *arvernensis* is genetically identical to *C*. *viridis* according to the phylogeny (see Fig 2). Interestingly four strains previously assigned to *Choricystis* showed the same characteristics (one-sided mucilage cap of the cell, oblique cell division) as the other *Coccomyxa* strains, and were therefore incorrectly identified by the isolator. As shown in Fig 2, species assigned to *Coccomyxa* and *Pseudococcomyxa* are mixed in our phylogeny, which poses a question about the morphological differences between both genera. As mentioned above, both genera differ only in the production of mucilage. Whereas *Pseudococcomyxa* is characterized by a one-sided mucilage cap at one end of the cell, the cells of *Coccomyxa* are surrounded by mucilage [22, 36]. To prove whether mucilage production is a stable morphological character of *Coccomyxa*/*Pseudococcomyxa* species, we investigated nine strains (NIES 2166, CAUP H5101, SAG 216-9a, ASIB V16, SAG 2254, SAG 49.84, SAG 216–1, SAG 216–4, SAG 2325) representing all the phylogenetic lineages presented in Fig 2 under starvation conditions. The strains were investigated after 6 weeks of cultivation in 1:20 diluted medium containing 1% glucose. Seven of the nine investigated strains showed no morphological changes during the starvation experiment. Only the strains SAG 49.84 (*C*. *dispar*) and SAG 216–4 (*C*. *viridis*) showed a noteworthy reaction. The cells of the strain SAG 216–4 started to produce individual mucilage caps, which were at least four times bigger than under standard conditions (Fig 5A–5C). The chloroplast appeared bleached and poorly visible because most of the cells were strongly vacuolized. Due to the large mucilage caps, cells adhered in huge colonies forming thick layers. The strain SAG 49.84 completely changed its typical morphology during the starvation experiment (Fig 5D–5K). The cells were surrounded by a vast mucilage layer and showed similarities with the type description of *Coccomyxa dispar* [22, 36]. The cells had very pale chloroplasts and many vacuoles in the cytoplasm. After staining with methylen blue the mucilage showed a light structure, an intensively stained part surrounding each cell and a more fine common mucilage surrounding all the cells. Algae grown on agarized medium produced a thick mucilagenous crust, whereas a softer type of mucilage occurred in liquid medium. After the documentation of their morphology, cells were transferred to the nutrient-rich 3NBBM+V medium and checked again after two weeks. The cells had completely lost their mucilage. The mature vegetative cells became spherical. Many small vacuoles were observed in the cytoplasm. The young cells looked *Coccomyxa*-like, but some mature vegetative cells produced some atypical autosporangia with 16–32 daughter cells. However, the typical reproduction with two to four cells formed by oblique division was also observed. Under these conditions the algae showed similarities to *Elliptochloris*. The starvation experiment was repeated three times with identical results.

**Fig 6:**
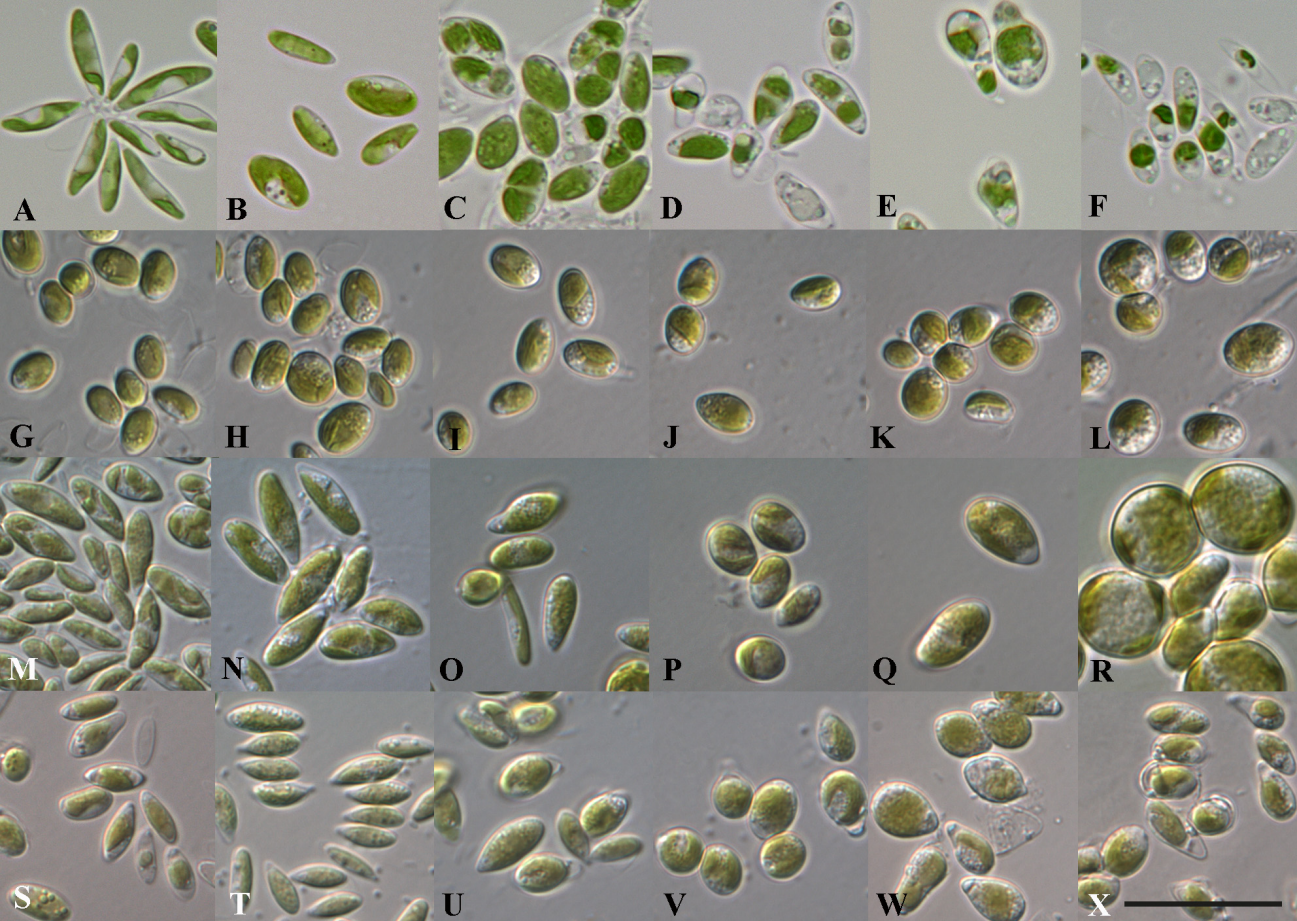
Phenotypic plasticity of various *Coccomyxa* strains under different salinities. A-F *C*. *polymorpha* (CAUP H5101), G-L *C*. *subellipsoidea* (CCAP 812/3), M-R *C*. *simplex* (CCAP 216/24), and S-X *C*. *vinatzeri* (ASIB V16). A, G, M and S 3BBM+V (0.2% NaCl; control); B, H, N, and T 3N-BBM+V with 0.6% NaCl; C, I, O, and U with 1.2% NaCl, D, J, P, and V with 1.8% NaCl, E, K, Q, and W with 2.5% NaCl, F, L, R, and X with 3.3% NaCl. Scale bar = 20 μm.

**Fig 7:**
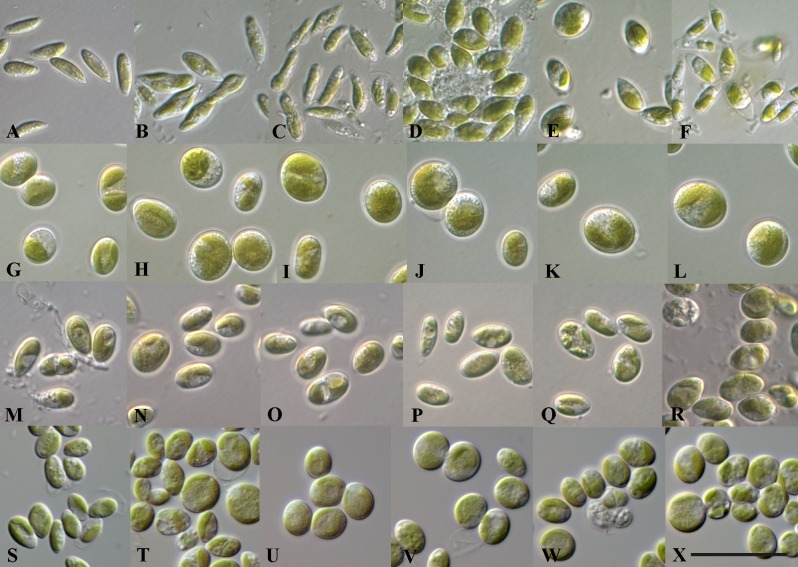
Phenotypic plasticity of various *Coccomyxa* strains under different salinities. A-F *C*. *galuniae* (CCAP 211/97), G-L *C*. *viridis* (SAG 216–1), M-R *C*. *viridis* (SAG 216–4), and S-X *C*. *viridis* (SAG 2325). A, G, M and S 3N-BBM+V (0.2% NaCl; control); B, H, N, and T 3N-BBM+V with 0.6% NaCl; C, I, O, and U with 1.2% NaCl, D, J, P, and V with 1.8% NaCl, E, K, Q, and W with 2.5% NaCl, F, L, R, and X with 3.3% NaCl. Scale bar = 20 μm.

To investigate the phenotypic plasticity of *Coccomyxa*, we studied the morphological changes of 34 strains at different salinities. Figs 6 and 7 illustrate the morphological plasticity of eight strains at different salinities representing six of the seven defined *Coccomyxa* species. Generally the following reactions resulting from higher salinity have been observed: (i) the cell wall became thicker under salinity stress (ii) the cell size increased under 0.6 and 1.2% salinity (iii) the cell number of sporangia containing 4 to 8 daughter cells increased, which is not typical for natural and standard culturing conditions (3N-BBM+V medium), respectively; (iv) in saline media the lysis of the mother cell wall was inhibited, whereas it is usually very quickly dissolved under standard culturing conditions, but the typical mucilage caps, common for *Coccomyxa* under standard conditions, have not been observed during salinity stress, and (v) the cells became more vacuolized with increasing salinities.

Summarizing the reaction of all investigated strains to different salinities, it could be classified into three types: *sensitive*, *intermediately sensitive* and *robust*. To document the response to salt stress the cell size and shape were compared to those under standard conditions as control (Figs 6–7, and S3 Fig). The three types of reactions are documented in few examples for each types as follows: 1. *Sensitive*: CAUP H5101 (*C*. *polymorpha*; Fig 6A–6F), 2. *Intermediately sensitive*: CCAP 812/3 (*C*. *subellipsoidea*, Fig 6G–6L), CCAP 216/24 (*C*. *simplex*, Fig 6M–6R), ASIB V16 (*C*. *vinatzeri*, Fig 6S–6X), CCAP 211/97 (*C*. *galuniae*, Fig 7A–7F), and 3. *robust*: SAG 216–1 (Fig 7G–7L), SAG 216–4 (Fig 7M–7R) and SAG 2325 (all *C*. *viridis*; Fig 7S–7X). These reactions were also confirmed via the measurement of length and width and the resulting calculation of the length:width ratio as shown in S3 Fig. The cells of the most sensitive strain CAUP H5101 (*C*. *polymorpha*) became increasingly spherical starting at 0.6% salinity. This effect could also be also observed in other strains belonging to intermediately sensitive group. Only the robust group showed only minimal changes in their length:width ratio.

### Salinity dependent growth response of *Coccomyxa*

**Fig 8:**
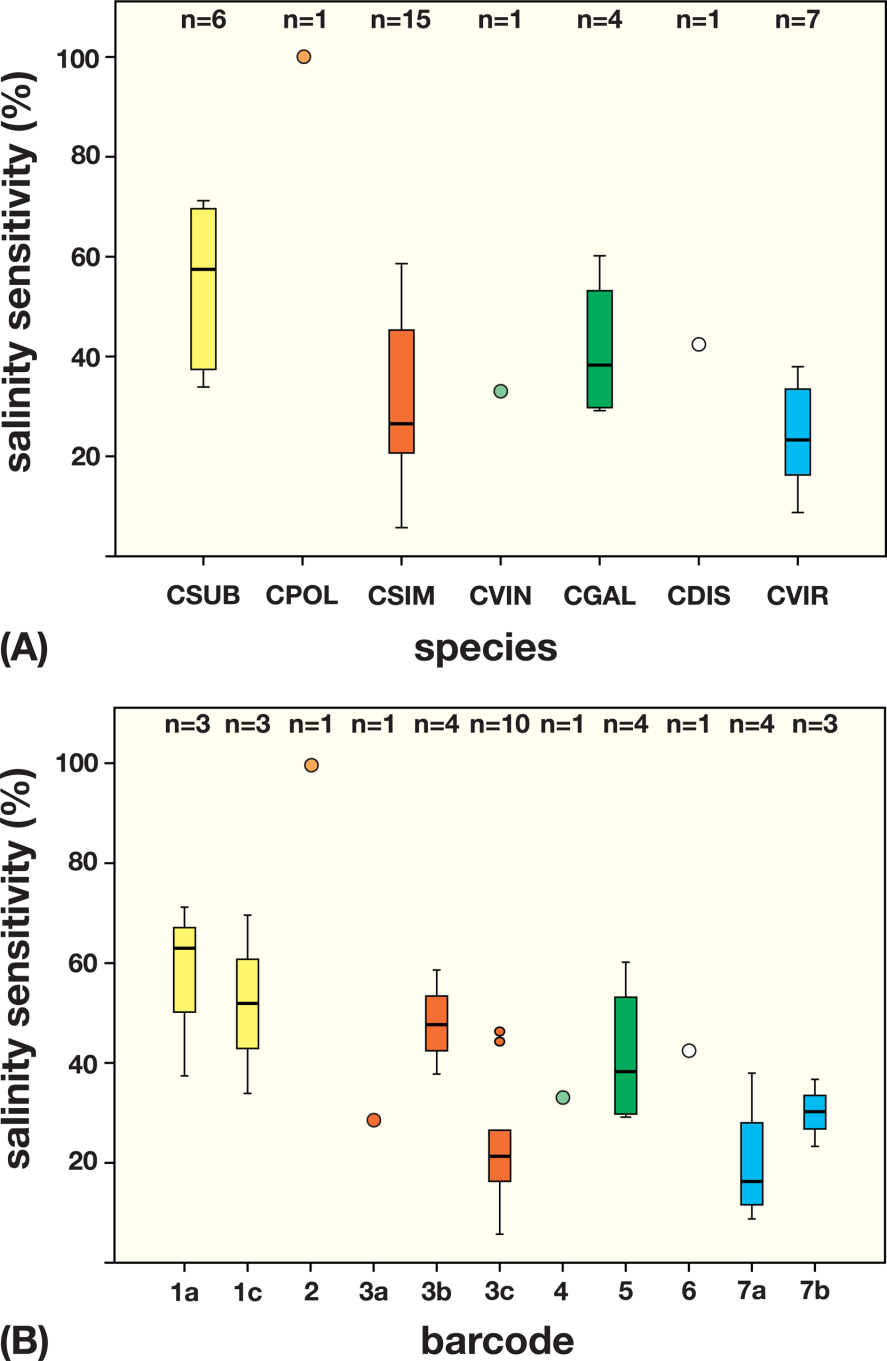
Salinity dependent sensitivity in percentage among (A) *Coccomyxa* species and (B) ITS-2 DNA barcodes presented in box-and-whisker diagrams. The most sensitive species *C*. *polymorpha* (CAUP H5101) is set as reference, value 100%. Abbreviations: CSUB = *C*. *subellipsoidea*, CPOL = *C*. *polymorpha*, CSIM = *C*. *simplex*, CVIN = *C*. *vinatzeri*, CGAL = *C*. *galuniae*, CDIS = *C*. *dispar*, CVIR = *C*. *viridis*.

As shown in the Figs 6 and 7, the strains showed different morphological responses to elevated salinity. To test the influence of changing salinity on growth, the growth rates of 34 strains were measured using the methods described above. In Fig 8, the relative sensitivity is summarized as percentages with the most sensitive reaction set as 100%. The growth of strain CAUP H5101 (*C*. *polymorpha*) was mostly inhibited already at 0.6% NaCl. A similar reaction was observed in *C*. *subellipsoidea* especially in strains that belong to the haplotype BC-1a. However, different strains of this species showed a wide range from sensitive to almost robust. In contrast, strains of *C*. *simplex* were more robust, but some (BC-3b) showed more inhibition of growth under higher salt concentrations. The growth of the other species *C*. *vinatzeri* (BC-4), *C*. *galuniae* (BC-5), *C*. *dispar* (BC-6), and *C*. *viridis* (BC-7a and BC-7b) was little or unaffected by elevated salinity, respectively. Interestingly, the photobionts of lichens belonging to *C*. *subellipsoidea* (BC-1c), *C*. *simplex* (BC-3c), and *C*. *viridis* (BC-7a) are more robust than their free-living relatives (BC-1a, BC-3a/b, BC-7b, respectively).

### New species of *Coccomyxa*

As demonstrated above, three lineages represented new species of *Coccomyxa*, which we formally describe here as follows.

#### *Coccomyxa polymorpha* Darienko et Pröschold sp. nov. (Fig 9A)

**Diagnosis:** Mature vegetative cells solitary or gathered in star-like structures, elongated-oval, asymmetric, very often curved, cell size (8.0)-9.0–11.0-(13.0) μm x (2.0)-2.5–3.0-(3.5) μm. Chloroplast lateral, trough-shaped, thin with small concavity in the middle where the nucleus is located. Pyrenoid is absent. Cell wall is thin. Reproduction by 2–4 autospores. Protoplast division is oblique. Liberation of autospores through the rupture of mother cell wall at one end. Rests of cell wall could occasionally be observed in the culture. The released autospores have the same morphology as mature vegetative cells.

**Fig 9:**
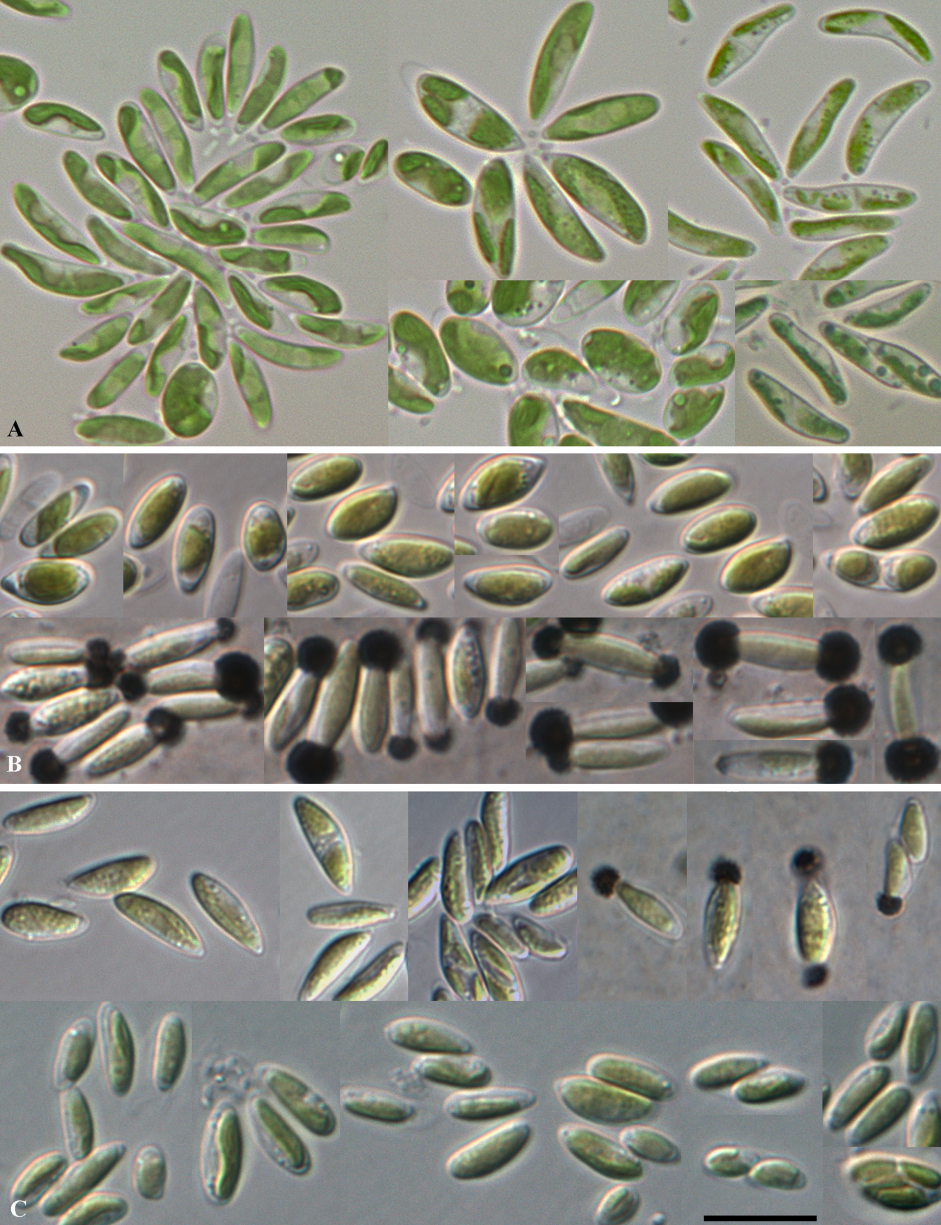
Morphology and its plasticity of the new species of *Coccomyxa*. **A**. *Coccomyxa polymorpha*, **B**. *Coccomyxa vinatzeri* (vegetative cells in the bottom row were stained with India Ink), and **C.** *Coccomyxa galuniae* (vegetative cells in the upper right corner were stained with India Ink). Scale bar = 10 μm.

Presence in culture of star-like structures may indicate possible production of a one side mucilage cap, but the staining with India ink was negative. In three-four week-old cultures, oval cells were observed, 6.9–8.6 x 4.2–5.0 μm in size. Occasionally, autospores stayed in the ruptured mother cell wall and produced the new autospores of second generation and connected in such way in small chain-like structures.

Morphologically similar to *Coccomyxa simplex*. Exact identification possible only using phylogenetic markers.

**Habitat:** unknown.

Type locality: unknown.

**Holotype** (designated here): Authentic strain CAUP H5101 permanently cryopreserved in a metabolically inactive state at CAUP.

**Iconotype** (designated here in support of the holotype): Fig 9A.

**Etymology:** The species name was chosen to describe variable cell shape of this alga.

#### *Coccomyxa vinatzeri* Darienko et Pröschold sp. nov. (Fig 9B)

**Diagnosis:** Mature vegetative cells are solitary, elongated oval, occasionally slightly pointed on one side, (4.0)-4.7–5.5-(6.2) x (1.9)-2.0–2.5-(3.5) μm. Chloroplast is lateral trough-shaped, massive, covering more than half of the cell volume. Pyrenoid is absent. Cell wall is thin. Reproduction by 2–4 autospores. Protoplast division is oblique and rarely traverse. Liberation of autospores through rupture of mother cell wall. Rest of autosporangia cell wall relative quick dissolved in the culture.

Morphologically similar to *Coccomyxa simplex*. Exact identification possible only using phylogenetic markers.

Habitat: soil.

**Type locality:** Pitschberg (2300 m a.s.l.), South Tyrol, Italy.

**Holotype (designated here):** Authentic strain SAG 2465 (= ASIB V16) permanently cryopreserved in a metabolically inactive state at SAG.

**Iconotype** (designated here in support of the holotype): Fig 9B.

**Etymology:** The species was named in honor of Dr. G. Vinatzer, who isolated it.

According to the description of G. Vinatzer, the cell size was 8.0–10.0 x 2.0–4.5 μm, but vegetative cells were smaller in our investigations.

*Coccomyxa galuniae* Darienko et Pröschold sp. nov. (Fig 9C)

**Diagnosis:** Vegetative cells are solitary and occasionally assembled in star-like structures. Cells are elongated-ovoid, straight or slightly dorsiventral, 7.0–10.5 μm × 2.3–4.0 μm, with the mucilage cap on the narrow end of the cell (or sometimes on both). The cell wall is thin and without any visible structures. Chloroplast parietal, trough-shaped without incisions, without pyrenoid. The nucleus is located in the middle part of cell and invisible without special staining. Asexual reproduction by 2–4 autospores. The reproduction is always by oblique division of protoplast. The autospores are liberated from sporangia by rupture of the cell wall on the broad end of the cell. Morphologically similar to *Coccomyxa simplex* and *Coccomyxa subellipsoidea*. Exact identification possible only using phylogenetic markers.

**Habitat:** freshwater and soil.

**Type locality:** Lake Stechlin, Brandenburg, Germany.

**Holotype:** The authentic strain CCAP 211/97 is cryopreserved in a metabolically inactive state at CCAP.

**Iconotype** (designated here in support of the holotype): Fig 9C.

**Etymology:** The species was named in honor of Prof. Dr. Margaret Galun for her contribution to investigations of different aspects of lichen symbiosis.

Comment: The four investigated strains of *Coccomyxa galuniae* have some morphological differences. Particularly, strains SAG 2253 and SAG 2254 have smaller cells– 6.9 × 2.5 μm in comparison with CCAP 211/97 (8.8 × 3.0 μm) and CCAP 812/5.

### The usage of different DNA Barcodes to discover the ecological preference, habitat specificity and biogeographical distribution pattern among *Coccomyxa* species

As demonstrated in this study, *Coccomyxa* species can clearly be distinguished using the ITS-2/CBC approach. However, the number codes presented in Fig 4 for each species cannot be directly used for the BLAST searching approach described above. In addition, this approach is solely based on ITS-2 sequences, which were available in GenBank. Unfortunately, complete ITS sequences were not available from all the *Coccomyxa* entries in GenBank. Therefore, besides ITS-2, we used the two common barcode regions V4 and V9 of SSU rDNA for BLAST searching. At first we discovered the haplotypes of the V4 and V9 regions for each species, which could be used as population identifiers. Comparison of the V4 and V9 regions (SSU) among the investigated strains demonstrated ten and seven variants of V4 (V4-A1-G in S6–S7 Tables) and V9 (V9-A-F2) within *Coccomyxa*, respectively. To determine whether these variants differ in paired or unpaired bases in their sequences, the secondary structures of both regions were analyzed. All *Coccomyxa* strains have three CBCs and two HCBCs in the V4 and V9 region compared to those of *Elliptochloris* and *Hemichloris* (highlighted in white boxes in S4 Fig). Most of the seven lineages within *Coccomyxa* (see Fig 2) were not supported by CBCs/HCBCs in the secondary structures (S4 Table), showing that the variants mostly differ in only unpaired bases of V4; only *C*. *polymorpha* and *C*. *viridis* could be differentiated by CBCs and HCBCs. In contrast, the V9 region showed CBCs/HCBCs, which could be used to discriminate the species of *Coccomyxa*.

As demonstrated in Fig 2, the three species of *Coccomyxa* (*C*. *subellipsoidea*, *C*. *simplex*, and *C*. *viridis*) are represented by more than five strains in contrast to the others, which have been found only once or a few times. To determine whether this distribution pattern of common and rare species could also be detected in nature, we searched for GenBank entries for the seven *Coccomyxa* species with the BLAST N algorithm described above. Using the variable regions (V4 and V9) of the SSU as well as the first three helices of ITS-2, 149 records were found in GenBank (summary in S6–S7 Tables). These entries showed 97–100% identity to our seven species: 49.0% (73 entries) were absolutely identical with one of our sequenced strains, and the remaining entries (marked with a number after the accession number in S6 Table) showed at least one base difference in their sequences compared to those. The occurrence of these base differences in the secondary structures was checked to determine whether those changes are real differences or results of sequencing mistakes. Most of the entries could also be identified at the species level. Only five entries (marked in pink in S7 Table) found using the V4 approach showed 97% similarity to *Coccomyxa*, but they have several CBCs and HCBCs in the secondary structure compared to our investigated strains. The phylogenetic analysis of the complete SSU rDNA sequences clearly demonstrated that these strains belong to another genus not closely related to *Coccomyxa* (data not shown). Interestingly, ten entries found with V4/V9 search also contained the ITS sequences, which were not recognized by the ITS2 search using the 97% threshold. Therefore, these ITS sequences have been aligned to ours according to the secondary structures and have been validated using the same ITS2 search algorithm used to determine their distribution. Nine additional records to our dataset were found. A comparison of all these sequences showed more than ten base differences from ours indicating that they might represent new lineages within *Coccomyxa*. To identify the phylogenetic position of these GenBank entries, the complete ITS rDNA sequences of all database entries found with ITS-2 search were aligned with those from this study. The phylogenetic analysis of this dataset is presented in S6 Fig and confirmed the distribution pattern described above. In addition, the secondary structure of each additional ITS-2 sequence found via our BLAST search has been folded and the ITS-2 barcode was extracted and translated into a number code as described above (see S2 Fig). The number codes for these entries were included into an alignment of the other *Coccomyxa* number codes. The distance phylogeny of this data set is also presented in S6 Fig. A comparison of both tree topologies showed a similar pattern at the species level but differed in the relationship among the lineages. However, the additional ITS sequences (highlighted in different grey colors) are possible new lineages and supported by new barcodes (BC-8-12) and CBCs/HCBCs (S4 Table) in the barcode region.

**Fig 10:**
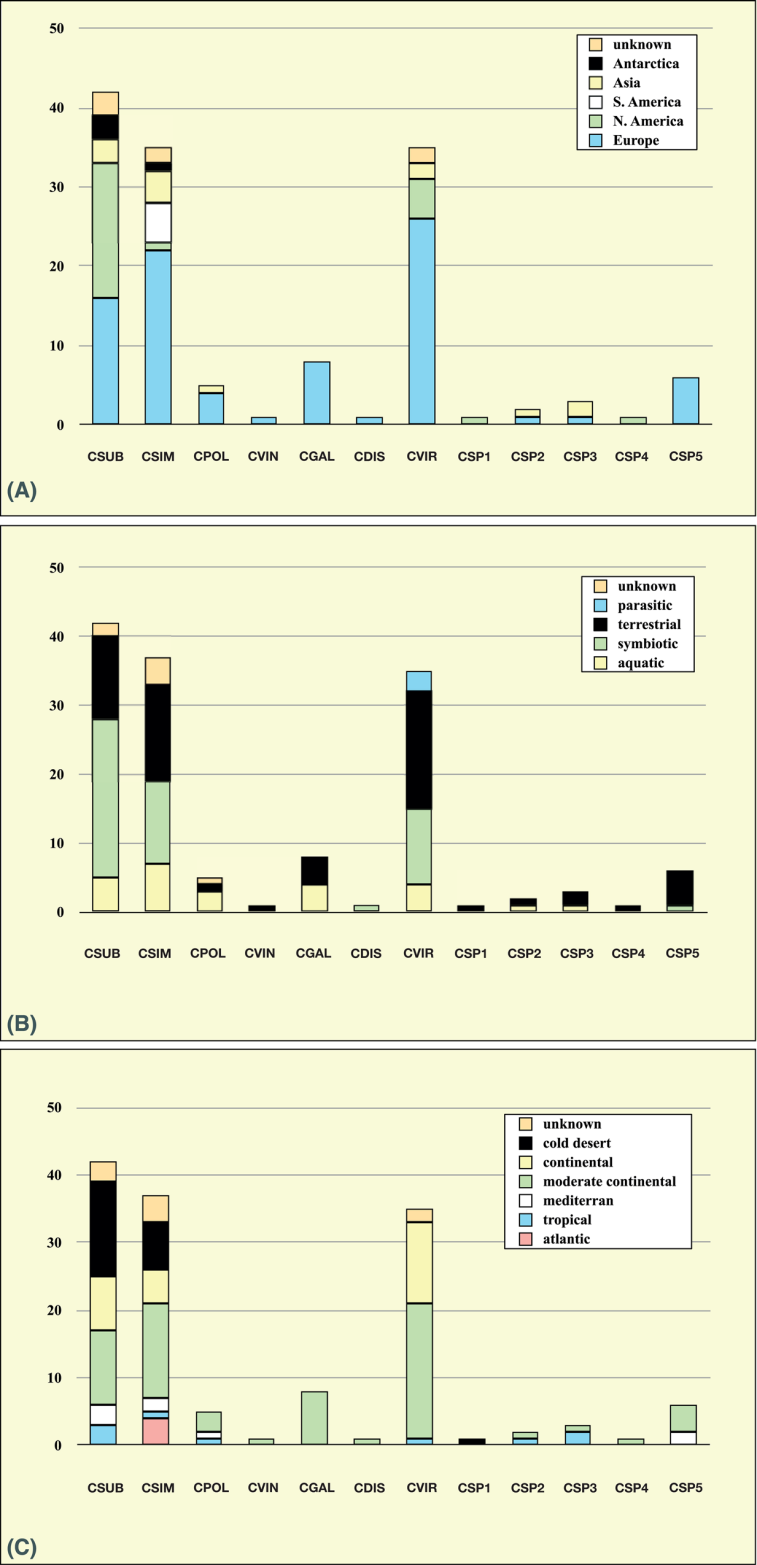
Distribution of species detected with ITS-2, V4 and V9 barcodes at the different continents (A), habitats (B), and climate zones (C). Detailed BLAST search results are summarized in S6–S7 Tables. Abbreviations: CSUB = *C*. *subellipsoidea*, CPOL = *C*. *polymorpha*, CSIM = *C*. *simplex*, CVIN = *C*. *vinatzeri*, CGAL = *C*. *galuniae*, CDIS = *C*. *dispar*, CVIR = *C*. *viridis*.

Summarizing, the comparison with GenBank entries clearly demonstrated that three species represented by multiple strains in our study are distributed worldwide and common in nature. In contrast, records for the other four species could be found only a few times (S6 Table). No records in databases were discovered for *C*. *dispar* (SAG 49.84) or *C*. *vinatzeri* (ASIB V16). Interestingly, several sequences represented additional new lineages within *Coccomyxa* and were confirmed via phylogenetic analyses and a BLAST search of the two outgroup taxa (only *Elliptochloris* or *Hemichloris* entries were detected with this search). For a better understanding of the distribution pattern the metadata of all GenBank entries (and its related publications, if available) were analyzed. For this purpose, the geographic origin, ecological preferences, and climate conditions have been summarized from those data (S7 Table, and Fig 10A–10C). Most of the records are from environmental studies in Europe and North America, and no records from Australia and Africa were found, most likely caused by a lack of investigations. The three common species (*C*. *subellipsoidea*, *C*. *simplex*, and *C*. *viridis*) seem to have a worldwide distribution (Fig 10A). The other species (*C*. *dispar*, *C*. *polymorpha*, *C*. *vinatzeri*, and *C*. *galuniae*) are rarely found in these investigations (Europe, North America, and Asia). All species can have different lifestyles and show no preferences to any climate. They occur free-living in aquatic and terrestrial habitats or as photobionts in lichens (Asco- and Basidiolichens); *C*. *viridis* is even parasitic in marine mussels (Fig 10B) and can be found in all climatic zones from cold deserts to tropical rain forests (Fig 10C). However, using only the results of the ITS-2 search and applying the ITS-2/CBC approach demonstrated above, some preferences could be discovered despite the lack of comprehensive environmental studies (S7 Fig). For example, the limited data from Antarctic and Asian samples showed that all records belong to *C*. *subellipsoidea* (BC-1a) and *C*. *simplex* (BC-3c), respectively. The photobionts of *C*. *simplex* and *C*. *viridis* belong to the barcode groups BC-3c or BC-7a, respectively (symbiont in BC-7b is a *Coccomyxa* isolate from *Gingko biloba*), and those of *C*. *subellipsoidea* mostly belong to BC-1c (*Omphalina* photobionts). The newly found lineages show no special distribution pattern, ecological preferences, or climatic conditions and can be found in the same habitats similar to the others in our study. Only the strains belonging to *C*. *sp*. 3 (BC-10) seem to have a preference for acidic habitats, but these strains should be investigated in detail to evaluate this hypothesis.

## Discussion

### Variability in *Coccomyxa*

Since the first description of the genus *Coccomyxa* by Schmidle [22], more than 50 species have been described according to morphology and lifestyle (free-living aquatic and terrestrial, symbiotic); however, a taxonomic revision using an integrative approach is still lacking. Considering only the morphology, delimitation at a generic or species level is difficult because many descriptions are based on studies of field samples without evaluation of these characteristics in culture experiments or are lacking distinct morphological features. Consequently, we investigated all strains originally assigned as *Coccomyxa* and *Pseudococcomyxa* that were available in public culture collections. Using an integrative approach (morphology and molecular phylogeny), the 41 strains could be clearly identified as *Coccomyxa*/*Pseudococcomyxa* (Fig 2). The molecular phylogeny of the strains belonging to *Coccomyxa*/*Pseudococcomyxa* showed a subdivision into seven lineages representing species. As shown in Fig 2, the strains originally designated with different species names of both genera are mixed together within the seven lineages indicating the difficulties of identification at the generic and species levels. Traditionally both genera differ in only their mucilage formation. However, as our study has clearly demonstrated, this feature cannot be used for differentiation at the generic level because the mucilage production is strongly dependent on the culture conditions (see Fig 5). The rare production of mucilage in natural samples explains that the type species of *Coccomyxa*, *C*. *dispar* (= *C*. *confluens*), has been found only occasionally [22, 36, 25]. According to our phylogenetic analyses, both genera belong to one monophyletic lineage. Consequently, the name *Coccomyxa* is proposed for all species as regulated by the principle of priority of the ICN. Because three lineages shown in Fig 2 contain authentic strains (= ‘type cultures’) of described species, we designated them according to the oldest valid species name. Authentic strains of other species originally described by Chodat [41] and Jaag [36] showed only few genetic differences to these species and therefore belong to *C*. *subellipsoidea*, *C*. *simplex*, and *C*. *viridis*, respectively (see Fig 2). The lack of distinct morphological characteristics and their variability under different conditions as well as their simplicity resulted in a high number of described species. This fact was already recognized by Jaag [36], who tested the phenotypic plasticity under different culture conditions. For the symbiotic isolates he proposed the usage of the lichen name for designating new *Coccomyxa* species. In our study, all strains from Jaag (except one) isolated from the ascolichen genera *Peltigera* and *Solorina* are very similar and belong to *C*. *simplex*. Only strain SAG 216–4, a photobiont of *Peltigera aphtosa*, represented *C*. *viridis*. In contrast, the strains isolated from the basidiolichens *Omphalina* and *Multiclavula* could be identified as *C*. *subellipsoidea* and *C*. *dispar*, respectively, indicating some species specificity to certain lichen genera. However, this must be verified by further studies especially because of the single record (SAG 49.84) for *Multiclavula* photobionts and general uncertainty whether the isolate is a photobiont or an epiphyte from a lichen [5]. All strains investigated in this study showed a high genetic variability (Fig 2) and phenotypic plasticity (Figs 5–8). Especially elevated salinity has an influence on cell morphology (Figs 5–7) and the growth rate of the investigated strains (Fig 8). Considering these results, species of *Coccomyxa* are highly adaptable to changing environments. Only high salt concentrations such as seawater have strong effects on the morphology and inhibit the growth rate of most species. This could explain why no strain isolated from marine habitats is available in public culture collection. The strain CCAP 216/18 described as *C*. *parasitica* [74] grows in marine media. However, despite that the original description most likely represented a *Coccomyxa* species, the strain CCAP 216/18 does not belong to *Coccomyxa*, which was demonstrated by Rodriguez et al. [32] based on partial SSU rDNA sequences. In addition, the morphology of the strain CCAP 216/18 does not correspond with the original diagnosis of *C*. *parasitica* described by Stevenson and South [74]. This example clearly demonstrates that it is necessary to identify *Coccomyxa* species using an integrative approach to avoid inaccurate records in culture collections and environmental studies.

### DNA barcoding and its implications for environmental studies

As described above, the taxonomic revision of microalgal genera and species using an integrative approach is very time consuming and requires scientific expertise. This is almost impossible for large scale environmental studies. For those investigations, a faster and accurate identification tool is necessary to identify these organisms at the generic and species levels. DNA barcoding has been introduced to solve this problem [7]. Although certain molecular barcode markers were proposed for animals, fungi, and higher plants (see above), the situation for microorganism (especially microalgae) is more complex because these organisms are genetically much more diverse. Phylogenetic analyses of microalgae are mostly based on SSU rDNA sequences; therefore two variable regions (V4 and V9) have been proposed as so-called pre-barcode markers [11–13]. For green algae, Hall et al. 2010 [75] have assessed several markers for DNA barcoding (SSU, UPA, *rbcL*, *tufA*, and *coxI*), and concluded that *rbcL*, ITS-2 and *tufA* are most promising for the usage as barcodes. In addition, the conserved region of the ITS-2 rDNA sequences often used for phylogenetic studies at species level has been proposed as barcode marker by Demchenko et al. [17]. In this study, we evaluated three markers of nuclear ribosomal operon mentioned above for species identification of *Coccomyxa*. The comparison of V4, V9, and ITS-2 showed that all markers can separate the seven species and therefore can be used for species identification as shown in S7 Table. Only the V9 region of *C*. *subellipsoidea* and *C*. *simplex* are identical and cannot differentiate the species if used alone. Along with a simple sequence comparison, we checked the secondary structures of all barcode regions to detect unique CBCs and HCBCs for species characterization. The seven species are supported by only CBCs/HCBCs using the ITS-2 barcode and the CBC concept as proposed by Coleman [16] can be used for species identification of *Coccomyxa*. The CBC concept needs to be critically evaluated in phylogenetic context before using for species delimitation [76, 77]. The ITS-2/CBC approach used in this study is based on the highly supported phylogeny and therefore the detected CBCs among the strains support the split into species. Using V4 and V9, CBCs/HCBCs differentiate only *Coccomyxa* from the outgroup taxa and are therefore useful for the delimitation at the generic level (see S4 Table). All these data confirmed that DNA barcoding using the proposed barcode markers can identify the genus *Coccomyxa* and its species and even populations (e.g. lichen photobionts) below species level. However, to identify the species boundaries, it is necessary to investigate species using an integrative approach as described in this study. Otherwise, the evidence of *Coccomyxa* can only be recorded as OTU level without any species information.

To determine whether these barcode approaches are applicable for species identification of *Coccomyxa* in environmental samples, we searched for the seven species using V4, V9, and ITS-2 by a BLAST algorithm (100% coverage, 97–100% sequence identity) in GenBank. We found approximately 150 entries, often not designated as *Coccomyxa* and sometimes even designated as genera of other algal classes (e.g. *Monodus* or *Ellipsoidion*, Eustigmatophyceae, Stramenopiles) or as unspecific, uncultured Trebouxiophyceae. Most entries could be assigned to a particular species, but to further validate the accuracy of the sequences, the secondary structure of the entries was folded; the results are summarized in S6 and S7 Tables. The range of sequence identity had to be adjusted depending on the barcode region. For example, with V4 only, sequences with 99–100% identity should be taken into account for species identification. A threshold of 97% can lead to a false identification even at the generic level as shown for five entries belonging to the genus *Xerochlorella*, which is not closely related to *Coccomyxa*. In contrast, this threshold is too high for the ITS-2 search. A combined search using V4/V9 and ITS-2 as previously described could identify only 13 records. However, these new records are found only if the threshold is set at the 80% level, but the use of this threshold for the ITS-2 search is problematic because entries belonging to other genera were also detected. To decide whether an entry belonged to *Coccomyxa* required a comprehensive investigation of the secondary structure. This is necessary if no sequence data of SSU rDNA are available from these entries. In conclusion, we provided a consensus secondary structure of ITS-2 (Fig 3) for easier analyses of *Coccomyxa* sequences derived from environmental studies.

Summarizing, the three barcode regions used in this study are suitable for identification of *Coccomyxa* species, but the evaluation of these regions using secondary structure is absolutely necessary to avoid ambiguous results. A comparison of all records identified with V4 showed only base differences in the helices E23_1 and E23_2 among the 229 base pairs (S4 Fig). Using the 97% threshold only seven base differences (including ambiguities or sequencing mistakes) were enough to find ‘new’ species or OTUs as recorded. In our study, we demonstrated that V4 is a good barcode marker for delimitation at the generic and sometimes at the species level, but the sequences must be accurate. The base differences detected in V4 mostly occurred in unpaired regions of the helices E23_1 and E23_2, which makes evaluation difficult. In contrast, the few differences observed in V9 mostly represented CBCs/HCBCs, even though this region is almost half as long as V4 (106 bp). Using this short region, no misidentifications were observed, in contrast to the V4 region, for which records of other genera have been erroneously detected. With exclusive use of SSU rDNA sequences, species of *Coccomyxa* can be identified only by combining V4 and V9. For the detection of possible new species of *Coccomyxa*, a combination of V9 and ITS-2 is the appropriate solution as demonstrated in S6 and S7 Tables and S6 Fig.

The records found in GenBank using these approaches (V4/V9/ITS-2) have clearly demonstrated that the biodiversity of *Coccomyxa* in nature is slightly higher, but dominated by the three abundant species (*C*. *subellipsoidea*, *C*. *simplex*, and *C*. *viridis*) presented in our phylogeny (Fig 2). The five new lineages found in environmental samples could represent new species, as indicated by the phylogenetic analyses (S6 Fig) and CBCs in the ITS-2 barcode regions (BC-8 to BC-12), but this must be verified with an integrative approach using cultured material. Most *Coccomyxa* strains available in public culture collections are easy to cultivate; therefore the integrative approach can be used. Some studies have even shown that *Coccomyxa* can occur as contaminant in stock solutions of culture media or distilled water [34] or in cooling water of nuclear plants [35], which confirmed that *Coccomyxa* has no special requirements for certain nutrients for growth and is rather undemanding. *C*. *subellipsoidea*, *C*. *simplex*, and *C*. *viridis* are the common species in environmental samples; the rare species represented by only one or few strains in our study also remain as rare species in nature. Only less than ten records of *C*. *polymorpha* and *C*. *galuniae* have been detected in GenBank without an entry of *C*. *dispar* and *C*. *vinatzeri*. The analysis of the metadata clearly demonstrated that the *Coccomyxa* species are distributed worldwide and confirmed their occurrence, especially of the three common species, in different habitats (Fig 10 and S7 Fig). Whether the rare species occupy a special ecological niche or are distributed only locally cannot be decided at this stage. For example, the strain ASIB V16 (*C*. *vinatzeri*) has been isolated from soil collected from the Dolomites (South Tyrol, Italy), which is the only information provided by Vinatzer [23]. The ecological preferences and distribution pattern of this and other rare species cannot be derived from the published data or the entries in GenBank. Despite the evidence of *Coccomyxa* in natural samples, very little is known about its ecological preferences, even for the common species. Several species names of *Coccomyxa* that are invalid according to the ICN (lack of designation of a type; *nomina nuda*) have been published because of their occurrence in specific habitats. *C*. *actinabiotis* (CCAP 216/25), found in cooling water of a nuclear plant [35], and *C*. *onubensis* (ACCV1), found in acidic water [78] represent new lineages of *Coccomyxa* (*C*. *sp*. 2, BC-9a/b and *C*. *sp*. 3, BC-10, respectively in S2 Fig). Falagan et al. [79] isolated two *Coccomyxa* strains from two acidic pit lakes (Spain) that have identical sequences according to the authors. The V4 regions of both strains showed three differences compared to *C*. *subellipsoidea* and *C*. *polymorpha*, which demonstrated the difficulties of species identification if V4 is used alone. Unfortunately, V9 and ITS-2 is not available for both strains, but using the entire 1500 bases provided from both strains and comparing them with ACCV1 showed that they are identical with *C*. *sp*. 3, which indicates that this lineage possibly prefers acidic habitats. However, Verma et al. [27] found an acid-tolerant isolate from Boomerang Lake (Ontario, Canada), which could be clearly identified as *C*. *viridis* using V4/V9 in our study (S6 and S7 Tables).

Almost all habitats can be occupied by *Coccomyxa* species. Only free-living, marine species are unknown so far, which is confirmed by the lack of GenBank entries from environmental studies of marine phytoplankton. Rodriguez et al. [32], Vázquez et al. [80], and Syasina et al. [33] found *Coccomyxa* as parasite of three mussels, *Mytilus edulis*, *Panopea abbreviata*, and *Modiolus modiolus*, which all belong to *C*. *viridis*, the most salinity-robust species in our study (see Fig 8). However, the intracellular parasitic lifestyle in the mussel muscle does not mean that the algal cells are exposed to seawater, but the question remains open how their dispersal among the different hosts occurs.

In conclusion, *Coccomyxa* is distributed worldwide and has a highly flexible lifestyle as shown in this study. Its occurrence in environmental samples of all types is very likely; therefore a clear molecular identification at the species level is necessary to understand its role in those ecosystems.

### Beyond Coccomyxa

Integrative taxonomy and its applications for environmental studies as demonstrated in this study for *Coccomyxa*, is a powerful tool for describing the biodiversity of microorganisms in different habitats. As we have shown, the combination of analyzing cultured material and DNA barcoding of environmental samples can provide insights into distribution patterns and ecological preferences.

Despite the common usage of V4 as barcode marker, we recommend a combined analysis using V9 and ITS for species identification of *Coccomyxa* in environmental samples as follows: (i) the region of V9 and ITS (~ 900 bp) should be sequenced double stranded and assembled without ambiguities; (ii) the secondary structures of V9 and ITS-2 should be folded using different folding programs; (iii) based on the foldings the sequences should be checked for accuracy; (iv) the V9 and the conserved part of ITS-2 can be searched using the BLAST algorithm as previously described; and (v) entries with 100% identity by 100% coverage are clearly identified at species level, and the others can be analyzed using phylogenetic and/or ITS-2 barcode approaches as described in this study. With these methods, the hidden diversity can be recognized and new strains of *Coccomyxa* can be isolated for further investigations using the integrative taxonomy demonstrated here.

This guideline is applicable not only for *Coccomyxa*, but also other groups of microalgae and even protists. The method of analyzing ITS sequences presented in this study cannot only be used for describing interspecific variability, but also for evaluating intraspecific differences (multiple copies within one organism) as demonstrated here for the entries highlighted in blue in S7 Table. A comparison of these sequences showed that the differences are located outside of the barcode region (conserved region of ITS-2; grey and black circled in the consensus structure; see Fig 4) or represent sequencing errors. The analyses of ITS secondary structures can help evaluate data from environmental studies and provide additional information about species boundaries and delimitation for algae, protists and fungi. For the latter group of organisms, ITS is the official barcode marker [9], and still under controversial discussion [81, 82]. It is noteworthy that the usage of ITS-2 as barcode marker, as demonstrated in this study, facilitates the identification of organisms at the species or OTU level.

## Supporting Information

S1 Fig SSU rRNA secondary structure model of *Coccomyxa subellipsoidea* (NIES 2166) with the two barcode regions V4 and V9 highlighted. The numbering of the helices follows according to Wuyts et al. [83].
(PDF)

S2 Fig ITS-2 secondary structures of each *Coccomyxa* strain investigated in this study (in green) and the newly found GenBank entries (in orange). The barcode region is numbered in white boxes and marked in blue for each helices. The line structure of the ITS-2 was drawn with PseudoViewer. Incomplete entries marked with an asterisk have been completed to fold the secondary structure. The added bases marked in lowercase letters.
(PDF)

S3 Fig The variability of length:width ratio depending on different salinities among selected *Coccomyxa* species in box-and-whisker diagrams. (PDF)

S4 Fig Secondary structure models of V4 and V9 (SSU) of *Coccomyxa subellipsoidea* (NIES 2166). The numbering of the helices follows according to Wuyts et al. [83]. The CBCs/HCBCs characterizing *Coccomyxa* species in comparison to *Elliptochloris bilobata* and *Hemichloris antarctica* are highlighted in white boxes.
(PDF)

S5 Fig Molecular phylogeny of representatives belonging to the Trebouxiophyceae based on SSU rDNA sequence comparisons. The phylogenetic tree shown was inferred using the maximum likelihood method based on a concatenated data set of 1780 aligned positions of 59 taxa using PAUP 4.0b10. For the analysis, the GTR+I+G model (base frequencies: A 0.2460, C 0.2225, G 0.2748, T 0.2567; rate matrix: A-C 1.0276, A-G 2.3668, A-T 0.9015, C-G 1.3952, C-T 5.5154, G-T 1.0000) with the proportion of invariable sites (I = 0.6279) and gamma distribution shape parameter (G = 0.5246) was chosen, which was calculated as the best model by Modeltest 3.7. The branches in bold are highly supported (Bayesian values > 0.95; bootstrap values > 70%) in all analyses.
(PDF)

S6 Fig Comparison of the molecular phylogeny of the *Elliptochloris*-clade (Trebouxiophyceae) based on ITS rDNA sequences (left) and the ITS-2 DNA Barcodes (right). The phylogenetic tree shown on the left was inferred using the maximum likelihood method based on a data set of 895 aligned positions of 120 taxa using PAUP 4.0b10. The data set contains the sequences of the strains investigated in this study and the GenBank entries found with a BLAST search as described in the text. The number of records for each species is given in brackets after the color-coded species designation. The distance phylogeny of the number-coded ITS-2 DNA Barcodes is shown on the right.
(PDF)

S7 Fig Distribution of the detected ITS-2 DNA barcodes on different continents (A), habitats (B), and climate zones (C). Detailed BLAST search results are summarized in the S6–S7 Tables.
(PDF)

S1 Table Strains used in the present study. Accession numbers new in this study are marked in bold.
(PDF)

S2 Table Bootstrap (>50%) and Bayesian support (>0.95) of branches 1–23 highlighted in Fig 2 using different phylogenetic methods described in details in Material and Methods for the concatenated data set of SSU and ITS rDNA sequences as well as of both genes separately. (PDF)

S3 Table Topology tests for eight user-defined trees derived from Fig 2 (= best tree), with a focus on the species boundaries within the genus *Coccomyxa*. The user-defined trees were generated manually in TreeView. The log-likelihood values of each tree were calculated in PAUP. The observed difference in-lnL (Obs) and p-values of the approximately unbiased test (AU), unweighted (KH) and weighted (wKH) Kishino-Hasegawa test, and unweighted (SH) and weighted (wSH) Shimodaira-Hasegawa test were calculated in CONSEL. P-values < 0.05 indicate a significant rejection at 5% level and marked with an asterisk.
(PDF)

S4 Table Pairwise comparison of the different barcode regions among the *Coccomyxa* species. Upper right corner = number of CBCs; lower left corner = number of HCBCs. (A) V4 region of SSU rDNA, (B) V9 region of SSU rDNA, and (C) ITS-2 DNA barcode (including the newly found barcodes of the BLAST search).
(PDF)

S5 Table Salinity dependent sensitivity in percentage among *Coccomyxa* species calculated by the decline of the growth rate from optimal (0.2 or 0.6% NaCl) to pessimal conditions (1.8, 2.5, or 3.3% NaCl, respectively). (PDF)

S6 Table BLAST N search results of the different barcode regions among the *Coccomyxa* species (100% coverage; 97–100% identity). (A) V4 region of SSU rDNA, (B) V9 region of SSU rDNA, and (C) ITS-2 DNA barcode. The grade of identities from 97–100% are color-coded. The designation after the accession number showed 100% identity to one of the strain in our study, the number in brackets recognized base differences in the sequences compared to the *Coccomyxa* species in this study, which are explained in S7 Table. The designations of the different haplotypes and ITS-2 Barcode followed those of S7 Table.
(PDF)

S7 Table (A) ITS2-Barcode and population identifier of the evaluated *Coccomyxa* strains. (B) Meta data of the GenBank entries found by the BLAST search of the different barcode regions and its evaluation. The ITS-2 of the accession numbers highlighted in orange represented new lineages and the secondary structure is shown in S2 Fig. The entries under the column ‘culture/environment’ (C/E) in blue represent multiple copies of ITS-2. The uncertain species identification of some entries are marked with an asterisk. The entries with a number in brackets under the columns ‘V4’ ‘V9’ and ‘ITS-2’ are evaluated using the secondary structures of these regions. The entries in pink represent taxa, which do not belong to *Coccomyxa*. Details are written in the text.
(PDF)

S1 Datasets Alignments used in this study. The NEXUS files (Coccomyxa_SSU_ITS.nex, Coccomyxa_SSU.nex, and Coccomyxa_ITS.nex) were used as input files for PAUP and MrBayes analyses, the rna files (Coccomyxa_SSU_ITS.rna, Coccomyxa_SSU.rna, and Coccomyxa_ITS.rna) for the analyses using PHASE. To detect compensatory base changes in the secondary structures of ITS-1 and ITS-2, the aligned FASTA files (Coccomyxa_ITS1_struct.fas and Coccomyxa_ITS2_struct.fas) were used as input files for 4SALE.
(ZIP)
