# Supplementary material for: Evaluating the Species Boundaries of Green Microalgae (Coccomyxa, Trebouxiophyceae, Chlorophyta) Using Integrative Taxonomy and DNA Barcoding with Further Implications for the Species Identification in Environmental Samples
Source: PLoS One. 2015 Jun 16;10(6):e0127838. doi: 10.1371/journal.pone.0127838 (PMC4469705; doi:10.1371/journal.pone.0127838)

SSU rRNA secondary structure model of *Coccomyxa subellipsoidea* strain NIES 2166 accession number: AGSI00000000

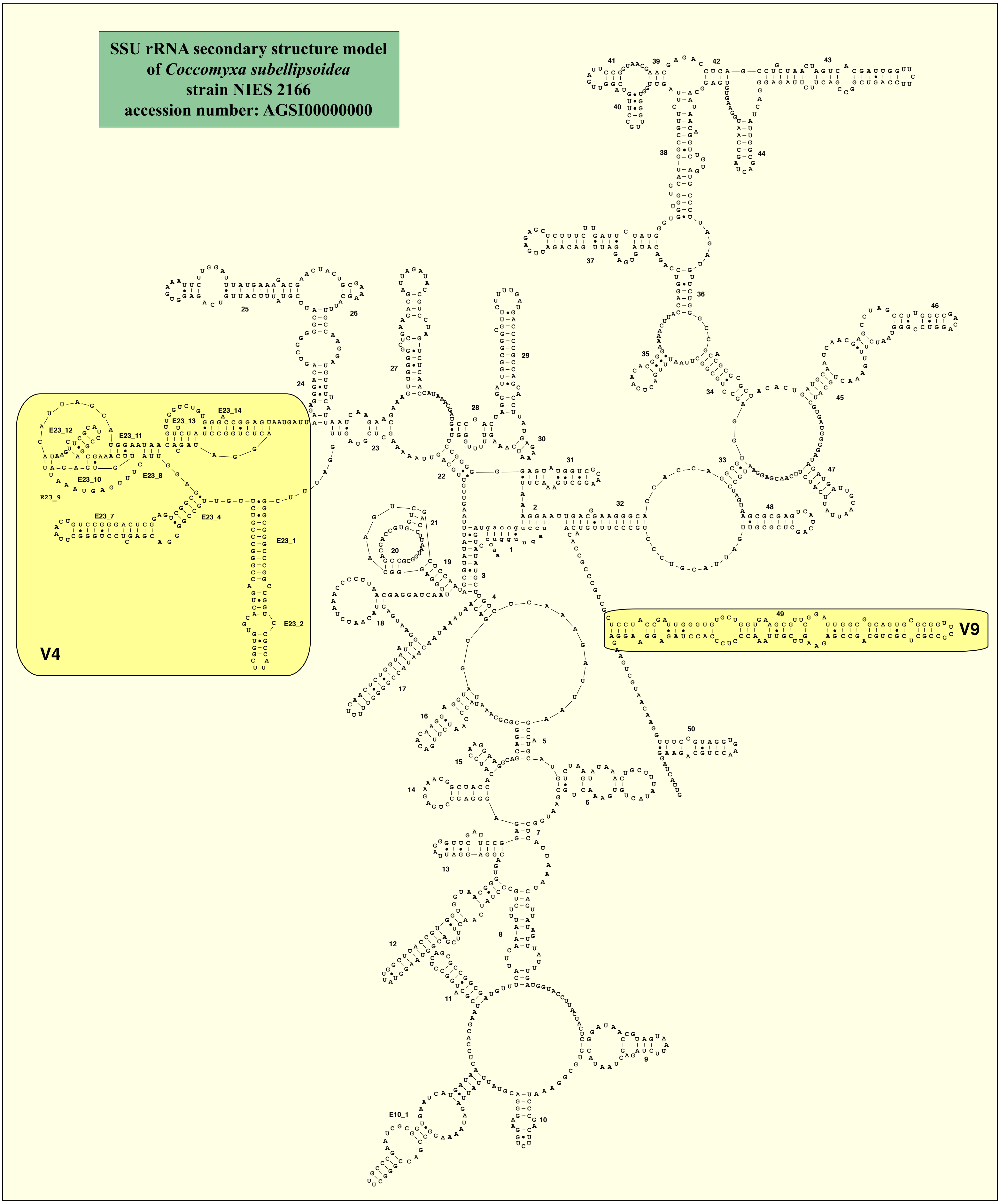

Supplement: S1 Fig — The numbering of the helices follows according to Wuyts et al. [83]. (PDF) [file pone.0127838.s001.pdf]
