# Supplementary material for: Evaluating the Species Boundaries of Green Microalgae (Coccomyxa, Trebouxiophyceae, Chlorophyta) Using Integrative Taxonomy and DNA Barcoding with Further Implications for the Species Identification in Environmental Samples
Source: PLoS One. 2015 Jun 16;10(6):e0127838. doi: 10.1371/journal.pone.0127838 (PMC4469705; doi:10.1371/journal.pone.0127838)
Supplement: S2 Fig — The barcode region is numbered in white boxes and marked in blue for each helices. The line structure of the ITS-2 was drawn with PseudoViewer. Incomplete entries marked with an asterisk have been completed to fold the secondary structure. The added bases marked in lowercase letters. (PDF) [file pone.0127838.s002.pdf]

Barcode OG-1

ITS-2 rRNA secondary structure model of *Elliptochloris bilobata* strain SAG 245.80 (OG-1) HG972969

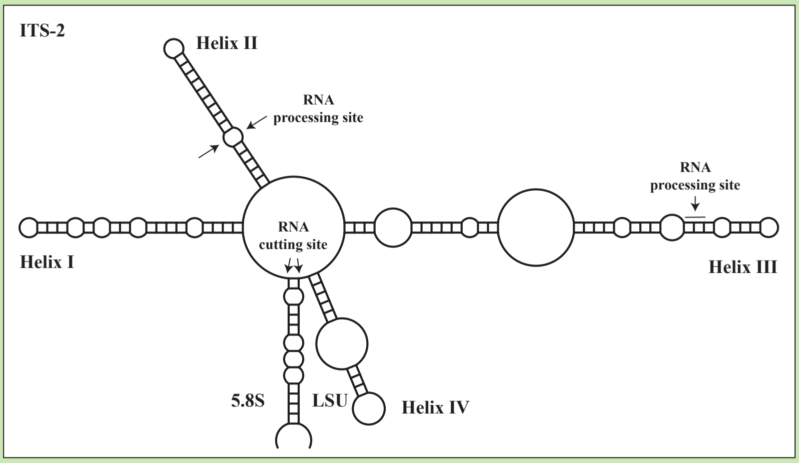

5.8S/LSU stem

```
---00000-000-0111-11
---12345-678-9012-34
---23442-184-2453-26
```

BC

```
GCC      C G G     C
   UGCCU A C UCGG UU->
   ||||| |  | ||•| |•
   ACGGA U G AGUC AG<-
AGA      C A -     C
------------------------
->CACCCC
------------------------
```

Helix I

```
11111
56789
64414
```

BC

```
     -     C   U  -    U
UCCAC UCCCU UGC UG GUCU C
•|||| •|||| •|| || |||| |
GGGUG GGGGA GCG AC CAGA U
     C     A   U  A    C
------------------------
AUC
------------------------
```

Helix II

```
22222222223
01234567890
63363374641
```

BC

```
      U          C
UGGUGG CUCAGCUUUC U
•||•|| |•||||•||| |
GCCGCC GGGUCGGAAG C
      U          G
------------------------
AAGCACAGA
-------------------------------------------------------
```

Helix III

```
333333----333444444444---------45555-5555--5566-666666667777
123456----789012345678---------90123-4567--8901-234567890123
334884----134383384448---------33341-4136--3361-343188888888
```

BC

```
      CG--      -  A   AUCACUCGAU     A    CU     G    U
GGC--C    AGCG-GG CCC          GGGCA CAGU  GGUA GCGA U
|||  |    |||| || |||          ||||| |||•  ||•| |||| |
CCG--G    UCGC-CC GGG          CCCGU GUCG  CCGU CGCU C
      CACG      -  A   C---------     G    --     A    G
-------------------------------------------------------
CGCA
---------------------------
```

Helix IV

```
      ------    UCC
GGAAGU      UGGC   A
|•||||      ||||   |
CUUUCA      ACCG   U
      AACUAA    CUG
---------------------------
->
---------------------------
```

Barcode legend:

1 = A-U
2 = U-A
3 = G-C
4 = C-G
5 = G•U
6 = U•G
7 = mismatch
8 = deletion,single or unpaired bases

Barcode OG-2

ITS-2 rRNA secondary structure model of *Hemichloris antarctica* strain SAG 62.90 (OG-2) HG972970

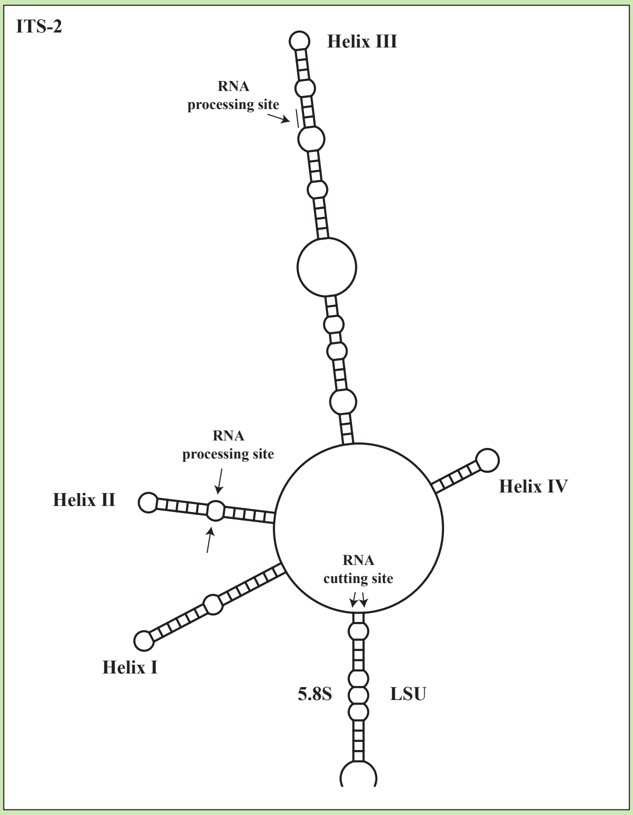

5.8S/LSU stem

```
---00000-000-0111-11
---12345-678-9012-34
---23442-184-2453-26   BC

GCC      C G G     C
   UGCCU A C UCGG UU->
   |||||  |  |  ||•|  |•
   ACGGA U G AGUC AG<-
AGA      C A -     C
------------------------
->CACCCCC
------------------------
```

Helix I

```
11111
56789
64424   BC

        C       U
UCCUCUCC UUUUUUG U
•||||||| ||||||| |
GGGAGAGG AAAAAAC C
        -       G
------------------------
ACC
------------------------
```

Helix II

```
22222222223
01234567890
63363374643   BC

      U      U
UGGUGG CUCGGC C
•||•|| |•|||| |
GCCGCC GGGCCG C
      U      G
------------------------
AAGAGCAGA
-------------------------------------------------------
```

Helix III

```
333333333444444444------45555-5555--5566-666666667777
123456789012345678------90123-4567--8901-234567890123
334688344384284448------33341-4136--3361-343188888888   BC

    CA     -  -   UUCAUAC     A    CU     G           C
GGCU  GCCG CU CCC        GGGCA CAGU  GGUA GCGA------ U
|||•  |||| || |||        ||||| |||•  ||•| ||||       |
CCGG  CGGC GA GGG        CCCGU GUCG  CCGU CGCU------ C
    --     U  C   C------     G    --     A           G
-------------------------------------------------------
GGCAGGAA
---------------------------
```

Helix IV

```
      UU
GUGUGC  \
||||||   G
CACACG  /
      AC
---------------------------
CAACACUCAACAUUC->
---------------------------
```

Barcode legend:

```
1 = A-U
2 = U-A
3 = G-C
4 = C-G
5 = G•U
6 = U•G
7 = mismatch
8 = deletion,single or unpaired bases
```

ITS-2 rRNA secondary structure model of *Coccomyxa subellipsoidea* strain NIES 2166 (BC-1a) AGSI00000000

Barcode 1a ITS2-A1

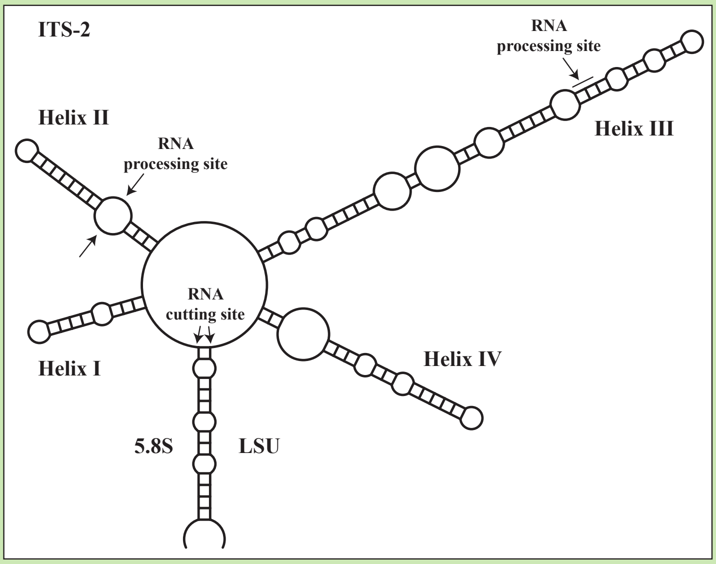

Barcode legend:

1 = A-U
2 = U-A
3 = G-C
4 = C-G
5 = G•U
6 = U•G
7 = mismatch
8 = deletion,single or unpaired bases

5.8S/LSU stem

```
---00000-000-0111-11
---12345-678-9012-34
---23442-154-2453-26   BC

GUC     C   G    C
   UGCCU AGC UCGG UU->
   ||||| |•| ||•| |•
   ACGGA UUG AGUC AG<-
AGA     C   -    C
------------------------
->UACCC
------------------------
```

Helix I

```
1111-1
5678-9
6414-4   BC

    C     A
UCAC CCCCC A
•||| ||||| |
GGUG GGGGG U
    C     C
------------------------
ACC
------------------------
```

Helix II

```
22222222223
01234567890
65347774443   BC

    AGU       G
UGGC   CCCGGUCG C
••||   |||||||| |
GUCG   GGGCCAGC A
    CCU       A
------------------------
AAGACCAGA
-------------------------------------------------------
```

Helix III

```
333-333-333444444-444---455--555555--5566-666666667777
123-456-789012345-678---901--234567--8901-234567890123
334-284-134181388-448---333--114132--3361-363884488884   BC

   -   G       GAC  ACAU   AA       CU    G   A        G
GGC U-C AGCA-AG   CC    GGG  AACAGU  GGUA GUG -CC----C C
||| | | |||| ||   ||    |||  ||||||  ||•| |•|  ||    | |
CCG A-G UCGU-UC   GG    CCC  UUGUCA  CCGU CGC -GG----G G
   A   G       AGA  ----   GC       --    A   -        A
-------------------------------------------------------
GGC
---------------------------
```

Helix IV

```
   AA---    -   U      U
AGG       UCGC CGG CGCCCU A
|||       |||| ||| |||||• |
UCC       AGCG GCC GCGGGG G
   CACAC    U   C      C
---------------------------
UUUC->
---------------------------
```

ITS-2 rRNA secondary structure model of *Coccomyxa subellipsoidea* strain NIES 2353 (BC-1a) HG972971

Barcode 1a
ITS2-A1

5.8S/LSU stem

```
---00000-000-0111-11
---12345-678-9012-34
---23442-154-2453-26
```

BC

```
GUC     C   G    C
   UGCCU AGC UCGG UU->
   ||||| |•| ||•| |•
   ACGGA UUG AGUC AG<-
AGA     C   -    C
------------------------
->UACCC
------------------------
```

Helix I

```
1111-1
5678-9
6414-4
```

BC

```
    C     A
UCAC CCCCC A
•||| ||||| |
GGUG GGGGG U
    C     C
------------------------
ACC
------------------------
```

Helix II

```
22222222223
01234567890
65347774443
```

BC

```
    AGU        G
UGGC   CCCGGUCG C
••||   |||||||| |
GUCG   GGGCCAGC A
    CCU        A
------------------------
AAGACCAGA
-------------------------------------------------------
```

Helix III

```
333-333-333444444-444---455--555555--5566-666666667777
123-456-789012345-678---901--234567--8901-234567890123
334-284-134181388-448---333--114132--3361-363884488884
```

BC

```
   -   G       GAC  ACAU   AA      CU    G   A        G
GGC U-C AGCA-AG   CC    GGG  AACAGU  GGUA GUG -CC----C C
||| | | |||| ||   ||    |||  ||||||  ||•| |•|  ||    | |
CCG A-G UCGU-UC   GG    CCC  UUGUCA  CCGU CGC -GG----G G
   A   G       AGA  ----   GC      --    A   -        A
-------------------------------------------------------
GGC
---------------------------
```

Helix IV

```
   AA---    -   U      U
AGG     UCGC CGG CGCCCU A
|||     |||| ||| |||||• |
UCC     AGCG GCC GCGGGG G
   CACAC    U   C      C
---------------------------
UUUC->
---------------------------
```

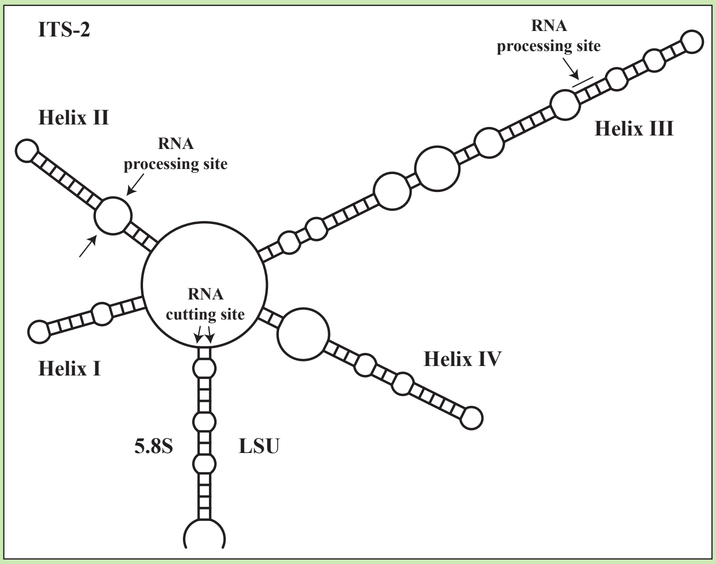

Barcode legend:

```
1 = A-U
2 = U-A
3 = G-C
4 = C-G
5 = G•U
6 = U•G
7 = mismatch
8 = deletion,single or unpaired bases
```

ITS-2 rRNA secondary structure model of *Coccomyxa subellipsoidea* strain CCAP 812/3 (BC-1a) HG972972

Barcode 1a
ITS2-A1

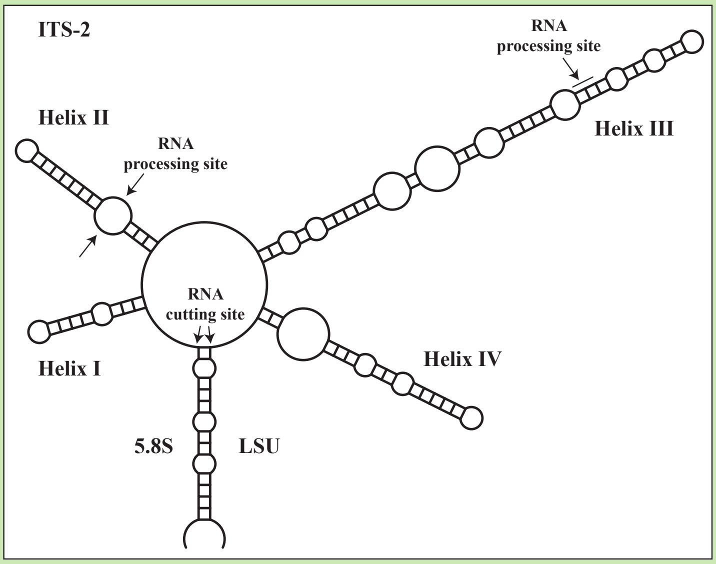

5.8S/LSU stem

```
---00000-000-0111-11
---12345-678-9012-34
```

```
---23442-154-2453-26
```

BC

```
GUC     C   G    C
   UGCCU AGC UCGG UU->
   ||||| |•| ||•| |•
   ACGGA UUG AGUC AG<-
AGA     C   -    C
------------------------
->UACCC
------------------------
```

Helix I

```
1111-1
5678-9
```

```
6414-4
```

BC

```
    C     A
UCAC CCCCC A
•||| ||||| |
GGUG GGGGG U
    C     C
------------------------
ACC
------------------------
```

Helix II

```
22222222223
01234567890
```

```
65347774443
```

BC

```
    AGU       G
UGGC   CCCGGUCG C
••||   |||||||| |
GUCG   GGGCCAGC A
    CCU       A
------------------------
AAGACCAGA
-------------------------------------------------------
```

Helix III

```
333-333-333444444-444---455--555555--5566-666666667777
123-456-789012345-678---901--234567--8901-234567890123
```

```
334-284-134181388-448---333--114132--3361-363884488884
```

BC

```
   -   G       GAC  ACAU   AA      CU    G   A        G
GGC U-C AGCA-AG   CC    GGG  AACAGU  GGUA GUG -CC----C C
||| | | |||| ||   ||    |||  ||||||  ||•| |•|  ||    | |
CCG A-G UCGU-UC   GG    CCC  UUGUCA  CCGU CGC -GG----G G
   A   G       AGA  ----   GC      --    A   -        A
-------------------------------------------------------
GGC
---------------------------
```

Helix IV

```
   AA---    -   U      U
AGG     UCGC CGG CGCCCU A
|||     |||| ||| |||||• |
UCC     AGCG GCC GCGGGG G
   CACAC    U   C      C
---------------------------
UUUC->
---------------------------
```

Barcode legend:

```
1 = A-U
2 = U-A
3 = G-C
4 = C-G
5 = G•U
6 = U•G
7 = mismatch
8 = deletion,single or unpaired bases
```

ITS-2 rRNA secondary structure model of *Coccomyxa subellipsoidea* strain NIES 2252 (BC-1a) HG972973

Barcode 1a ITS2-A2

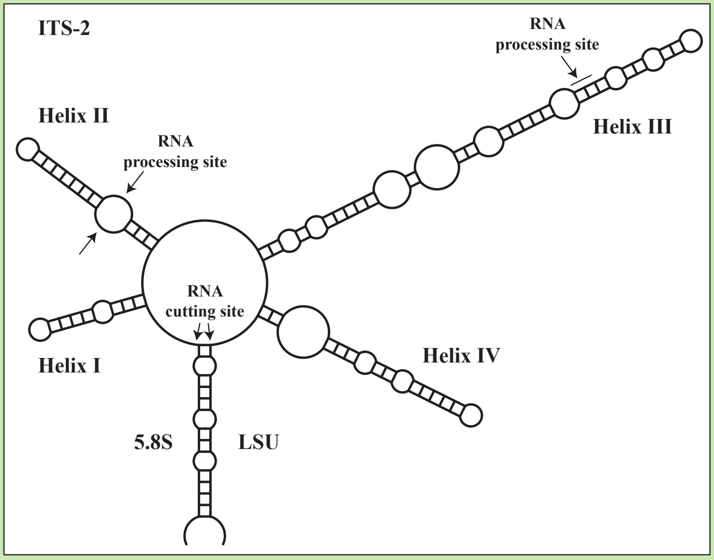


```
Barcode legend:

1 = A-U
2 = U-A
3 = G-C
4 = C-G
5 = G•U
6 = U•G
7 = mismatch
8 = deletion,single or unpaired bases
```

5.8S/LSU stem

```
---00000-000-0111-11
---12345-678-9012-34
---23442-154-2453-26   BC

GUC     C   G    C
   UGCCU AGC UCGG UU->
   ||||| |•| ||•| |•
   ACGGA UUG AGUC AG<-
AGA     C   -    C
------------------------
->UACCC
------------------------
```

Helix I

```
1111-1
5678-9
6414-4   BC

    C     A
UCAC CCCCC A
•||| ||||| |
GGUG GGGGG U
    C     C
------------------------
ACC
------------------------
```

Helix II

```
22222222223
01234567890
65347774443   BC

    AGU        G
UGGC   CCCGGUCG C
••||   |||||||| |
GUCG   GGGCCAGC A
    CCU        A
------------------------
AAGACCAGA
-------------------------------------------------------
```

Helix III

```
333-333-333444444-444---455--555555--5566-666666667777
123-456-789012345-678---901--234567--8901-234567890123
334-284-134181388-448---333--114132--3361-363884488884   BC

   -   G       GAC  ACAU   AA      CU    G   A        G
GGC U-C AGCA-AG   CC    GGG  AACAGU  GGUA GUG -CC----C C
||| | | |||| ||   ||    |||  ||||||  ||•| |•|  ||    | |
CCG A-G UCGU-UC   GG    CCC  UUGUCA  CCGU CGC -GG----G G
   A   G       AGA  ----   GC      --    A   -        A
-------------------------------------------------------
GGC
---------------------------
```

Helix IV

```
   AA---    -   U      U
AGG     UCGU CGG CGCCCU A
|||     |||• ||| |||||• |
UCC     AGCG GCC GCGGGG G
   CACAC    U   C      C
---------------------------
UUUC->
---------------------------
```

ITS-2 rRNA secondary structure model of *Coccomyxa subellipsoidea* strain CAUP H5105 (BC-1b) HG972974

Barcode 1b ITS2-A3

5.8S/LSU stem

```
---00000-000-0111--11
---12345-678-9012--34
---23442-154-2453--26   BC

GUC     C   G    CC
   UGCCU AGC UCGG  UU->
   ||||| |•| ||•|  |•
   ACGGA UUG AGUC  AG<-
AGA     C   -    C-
------------------------
->ACCC
------------------------
```

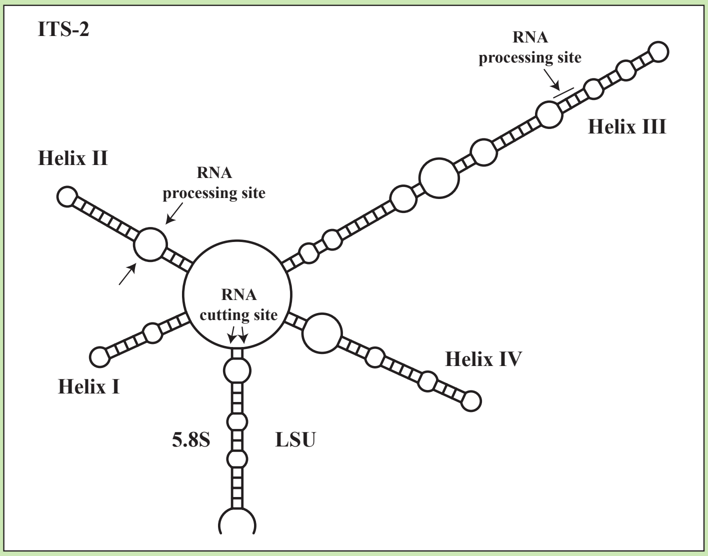

Helix I

```
1111-1
5678-9
6414-4   BC

    C     A
UCAC CCCCC A
•||| ||||| |
GGUG GGGGG U
    C     C
------------------------
ACC
------------------------
```

Barcode legend:

1 = A-U
2 = U-A
3 = G-C
4 = C-G
5 = G•U
6 = U•G
7 = mismatch
8 = deletion,single or unpaired bases

Helix II

```
22222222223
01234567890
65347774443   BC

    AGU        G
UGGC   CCCGGUCG C
••||   |||||||| |
GUCG   GGGCCAGC A
    CCU        A
------------------------
AAGACCAGA
-------------------------------------------------------
```

Helix III

```
333-333-333444444--444---455--555555--5566-666666667777
123-456-789012345--678---901--234567--8901-234567890123
334-284-154181383--448---333--114136--3361-363884488884   BC

   -   G         AC  ACAU   AA      CU    G   A        G
GGC U-C AGCA-AG-G  CC    GGG  AACAGU  GGUA GUG -CC----C A
||| | | |•|| || |  ||    |||  |||||•  ||•| |•|  ||    | |
CCG A-G UUGU-UC-C  GG    CCC  UUGUCG  CCGU CGC -GG----G A
   A   G         AA  ----   GC      --    A   -        A
-------------------------------------------------------
GGC
---------------------------
```

Helix IV

```
   AA--    -     G    U
AGG    UCGU CGGUC CCCU A
|||    |||• |||•| |||• |
UCC    AGCG GCCGG GGGG G
   CACA    U     A    C
---------------------------
UUUC->
---------------------------
```

Barcode 1c
ITS2-A4

ITS-2 rRNA secondary structure model of *Coccomyxa subellipsoidea* strain Wien C20 (BC-1c) HG972975

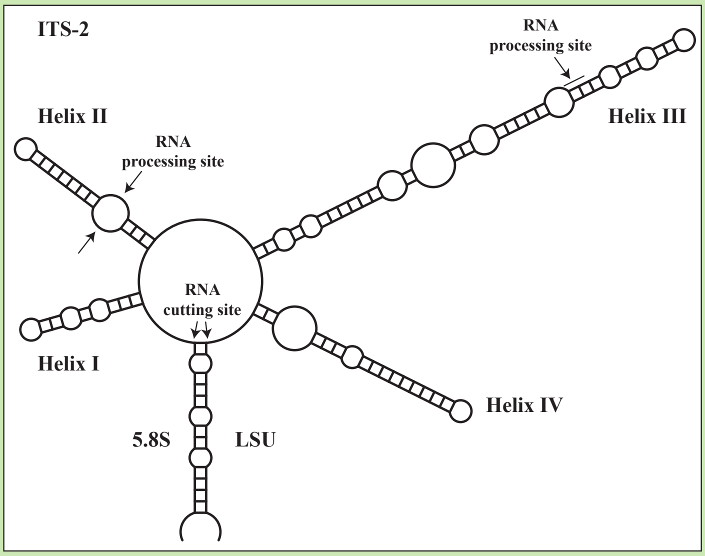

Barcode legend:

1 = A-U
2 = U-A
3 = G-C
4 = C-G
5 = G•U
6 = U•G
7 = mismatch
8 = deletion,single or unpaired bases

5.8S/LSU stem

```
---00000-000-0111-11
---12345-678-9012-34
---23442-154-2453-26
```

BC

```
GUC     C   G    C
   UGCCU AGC UCGG UU->
   ||||| |•| ||•| |•
   ACGGA UUG AGUC AG<-
AGA     C   -    C
------------------------
->UACCC
------------------------
```

Helix I

```
1111-1
5678-9
6414-4
```

BC

```
    -  -   A
UCAC CC CCC A
•||| || ||| |
GGUG GG GGG C
    C  A   C
------------------------
ACC
------------------------
```

Helix II

```
22222222223
01234567890
65347774443
```

BC

```
    AGU        G
UGGC   CCCGGUCG C
••||   |||||||| |
GUCG   GGGCCAGC A
    CCU        A
------------------------
AAGACCAGA
-------------------------------------------------------
```

Helix III

```
333-333-333444444--444---455--555555--5566-666666667777
123-456-789012345--678---901--234567--8901-234567890123
334-284-134181383--448---333--114136--3361-363884488884
```

BC

```
   -   G         AC  GCAU   AA      CU    G   A        G
GGC U-C AGCA-AG-G  CC    GGG  AACAGU  GGUA GUG -CC----C A
||| | | |||| || |  ||    |||  |||||•  ||•| |•|  ||    | |
CCG A-G UCGU-UC-C  GG    CCC  UUGUCG  CCGU CGC -GG----G A
   A   G         GA  ----   GC      --    A   -        A
-------------------------------------------------------
GGC
---------------------------
```

Helix IV

```
   AA--    -          U
AGG    UCGU CGGUCGCCCU A
|||    |||• |||•|||||• |
UCC    AGCG GCCGGCGGGG G
   CACA    U          C
---------------------------
UUUC->
---------------------------
```

ITS-2 rRNA secondary structure model of *Coccomyxa subellipsoidea* strain SAG 216-7 (BC-1c) HG972976

Barcode 1c
ITS2-A4

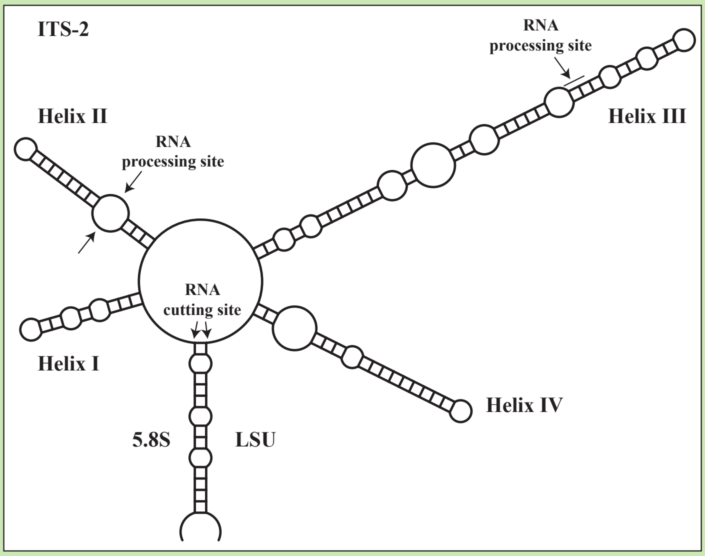

Barcode legend:

1 = A-U
2 = U-A
3 = G-C
4 = C-G
5 = G•U
6 = U•G
7 = mismatch
8 = deletion,single or unpaired bases

5.8S/LSU stem

```
---00000-000-0111-11
---12345-678-9012-34
```

BC

```
---23442-154-2453-26
```

```
GUC     C   G    C
   UGCCU AGC UCGG UU->
   ||||| |•| ||•| |•
   ACGGA UUG AGUC AG<-
AGA     C   -    C
------------------------
->UACCC
------------------------
```

Helix I

```
1111-1
5678-9
```

BC

```
6414-4
```

```
    -  -   A
UCAC CC CCC A
•||| || ||| |
GGUG GG GGG C
    C  A   C
------------------------
ACC
------------------------
```

Helix II

```
22222222223
01234567890
```

BC

```
65347774443
```

```
    AGU        G
UGGC   CCCGGUCG C
••||   |||||||| |
GUCG   GGGCCAGC A
    CCU        A
------------------------
AAGACCAGA
-------------------------------------------------------
```

Helix III

```
333-333-333444444--444---455--555555--5566-666666667777
123-456-789012345--678---901--234567--8901-234567890123
```

BC

```
334-284-134181383--448---333--114136--3361-363884488884
```

```
   -   G         AC  GCAU   AA      CU    G   A        G
GGC U-C AGCA-AG-G  CC     GGG  AACAGU  GGUA GUG -CC----C A
||| | | |||| || |  ||     |||  |||||•  ||•| |•|  ||    | |
CCG A-G UCGU-UC-C  GG     CCC  UUGUCG  CCGU CGC -GG----G A
   A   G         GA  ----   GC      --    A   -        A
-------------------------------------------------------
GGC
---------------------------
```

Helix IV

```
   AA--    -          U
AGG    UCGU CGGUCGCCCU A
|||    |||• |||•|||||• |
UCC    AGCG GCCGGCGGGG G
   CACA    U          C
---------------------------
UUUC->
---------------------------
```

ITS-2 rRNA secondary structure model of *Coccomyxa subellipsoidea* strain SAG 216-13 (BC-1c) HG972978

Barcode 1c ITS2-A4

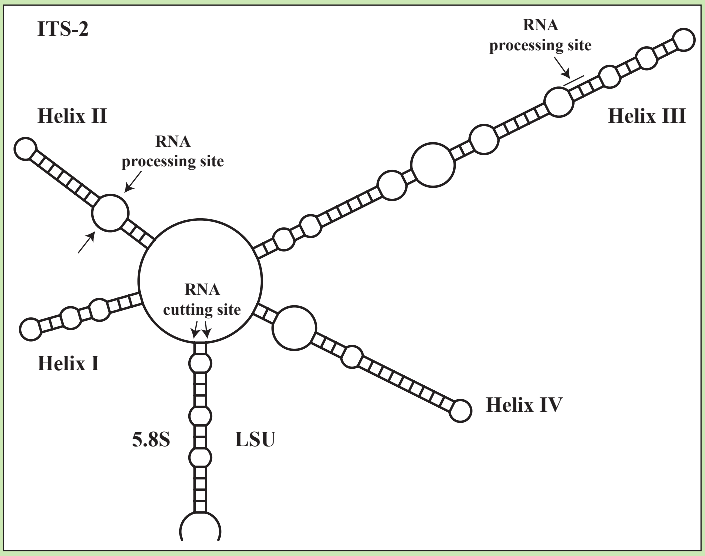

5.8S/LSU stem

```
---00000-000-0111-11
---12345-678-9012-34
---23442-154-2453-26   BC

GUC     C   G    C
   UGCCU AGC UCGG UU->
   ||||| |•| ||•| |•
   ACGGA UUG AGUC AG<-
AGA     C   -    C
------------------------
->UACCC
------------------------
```

Helix I

```
1111-1
5678-9
6414-4   BC

    -  -   A
UCAC CC CCC A
•||| || ||| |
GGUG GG GGG C
    C  A   C
------------------------
ACC
------------------------
```

Helix II

```
22222222223
01234567890
65347774443   BC

    AGU        G
UGGC   CCCGGUCG C
••||   |||||||| |
GUCG   GGGCCAGC A
    CCU        A
------------------------
AAGACCAGA
-------------------------------------------------------
```

Helix III

```
333-333-333444444--444---455--555555--5566-666666667777
123-456-789012345--678---901--234567--8901-234567890123
334-284-134181383--448---333--114136--3361-363884488884   BC

   -   G         AC  GCAU   AA      CU    G   A        G
GGC U-C AGCA-AG-G  CC    GGG  AACAGU  GGUA GUG -CC----C A
||| | | |||| || |  ||    |||  |||||•  ||•| |•|  ||     | |
CCG A-G UCGU-UC-C  GG    CCC  UUGUCG  CCGU CGC -GG----G A
   A   G         GA  ----   GC      --    A   -        A
-------------------------------------------------------
GGC
---------------------------
```

Helix IV

```
   AA--    -          U
AGG    UCGU CGGUCGCCCU A
|||    |||• |||•|||||• |
UCC    AGCG GCCGGCGGGG G
   CACA    U          C
---------------------------
UUUC->
---------------------------
```

Barcode legend:

```
1 = A-U
2 = U-A
3 = G-C
4 = C-G
5 = G•U
6 = U•G
7 = mismatch
8 = deletion,single or unpaired bases
```

ITS-2 rRNA secondary structure model of *Coccomyxa subellipsoidea* strain SAG 69.80 (BC-1c) HG972977

Barcode 1c
ITS2-A5

5.8S/LSU stem

```
---00000-000-0111-11
---12345-678-9012-34
---23442-154-2453-26
```

BC

```
GUC      C   G    C
   UGCCU AGC UCGG UU->
   ||||| |•| ||•| |•
   ACGGA UUG AGUC AG<-
AGA      C   -    C
------------------------
->UACCC
------------------------
```

Helix I

```
1111-1
5678-9
6414-4
```

BC

```
    -  -   A
UCAC CC CCC A
•||| || ||| |
GGUG GG GGG A
    C  A   C
------------------------
ACC
------------------------
```

Helix II

```
22222222223
01234567890
65347774443
```

BC

```
    AGU        G
UGGC   CCCGGUCG C
••||   |||||||| |
GUCG   GGGCCAGC A
    CCU        A
------------------------
AAGACCAGA
-------------------------------------------------------
```

Helix III

```
333-333-333444444--444---455--555555--5566-666666667777
123-456-789012345--678---901--234567--8901-234567890123
334-284-134181383--448---333--114136--3361-363884488884
```

BC

```
   -   G         AC  GCAU   AA       CU    G   A        G
GGC U-C AGCA-AG-G  CC    GGG  AACAGU  GGUA GUG -CC----C A
||| | | |||| || |  ||    |||  |||||•  ||•| |•|  ||     | |
CCG A-G UCGU-UC-C  GG    CCC  UUGUCG  CCGU CGC -GG----G A
   A   G         GA  ----   GC       --    A   -        A
-------------------------------------------------------
GGC
---------------------------
```

Helix IV

```
   AA--    -          U
AGG    UCGU CGGUCGCCCU A
|||    |||• |||•|||||• |
UCC    AGCG GCCGGCGGGG G
   CACA    U          C
---------------------------
UUUC->
---------------------------
```

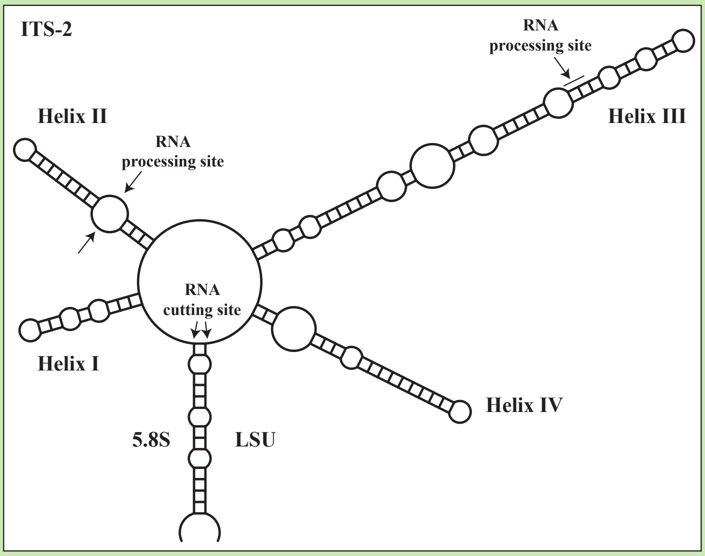

Barcode legend:

1 = A-U
2 = U-A
3 = G-C
4 = C-G
5 = G•U
6 = U•G
7 = mismatch
8 = deletion,single or unpaired bases

ITS-2 rRNA secondary structure model of *Coccomyxa polymorpha* strain CAUP H5101 (BC-2) HG972979

Barcode 2
ITS2-B

5.8S/LSU stem

```
---00000-000-0111-11
---12345-678-9012-34
---23442-154-2453-26   BC

GUC     C   G    C
   UGCCU AGC UCGG UU->
   ||||| |•| ||•| |•
   ACGGA UUG AGUC AG<-
GGA     C   -    C
------------------------
->CACCCC
------------------------
```

Helix I

```
11111
56789
64344   BC

      C      UU
UCGCCU CUUUCU  U
•||||| |||||•  |
GGCGGA GAAAGG  G
      C      CU
------------------------
ACG
------------------------
```

Helix II

```
22222222223
01234567890
65343374643   BC

      U        UG
UGGCGG CUCGGUCG  C
••|||| |•||||||  |
GUCGCC GGGCCAGC  A
      U        UC
------------------------
AAGACCAGA
-------------------------------------------------------
```

Helix III

```
33-3333-33344444--4444---4555--55555--5566-666666667777
12-3456-78901234--5678---9012--34567--8901-234567890123
33-4284-13418138--4448---3338--14136--3361-363182488884   BC

  -    G       GC-   AAUC   ACA     CU    G    C       U
GG CU-C AGCA-AG   CCC    GGG   ACAGU  GGUA GUGA UC----C U
|| || | |||| ||   |||    |||   ||||•  ||•| |•|| ||     | |
CC GA-G UCGU-UC   GGG    CCC   UGUCG  CCGU CGCU AG----G C
  G    G       GCA   ----   GGC     --    A    -       G
-------------------------------------------------------
-
---------------------------
```

Helix IV

```
    G  G  C--   AU
GGAA GC CG   CCG  \
|•|| || ||   |||  U
CUUU CG GC   GGC  /
    G  A  CAA   CA
---------------------------
UCACCACAUUUC->
---------------------------
```

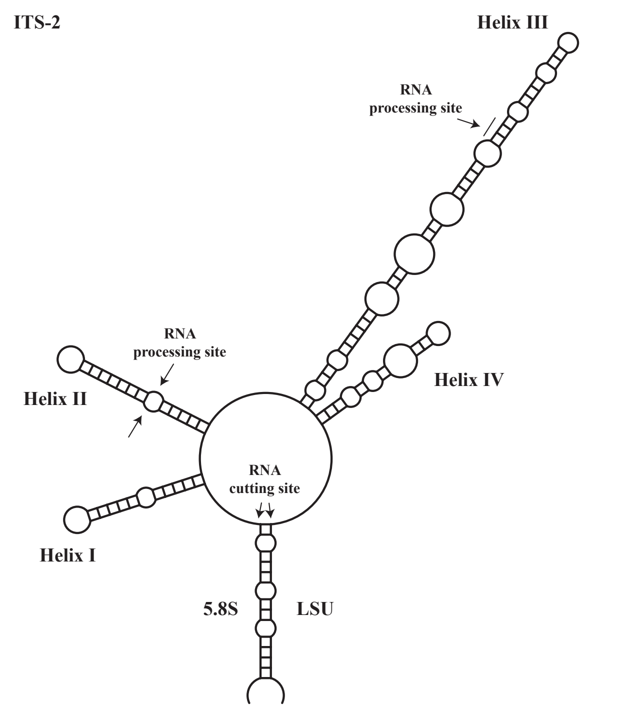

Barcode legend:

```
1 = A-U
2 = U-A
3 = G-C
4 = C-G
5 = G•U
6 = U•G
7 = mismatch
8 = deletion,single or unpaired bases
```

ITS-2 rRNA secondary structure model of *Coccomyxa simplex* strain SAG 216-3b (BC-3a) HG972980

Barcode 3a ITS2-C1

5.8S/LSU stem

```
---00000-000-0111-11
---12345-678-9012-34
---23442-154-2453-26
```

BC

```
GUC      C   G    C
   UGCCU AGC UCGG UU->
   ||||| |•| ||•| |•
   ACGGA UUG AGUC AG<-
AGA      C   -    C
------------------------
->CACCC
------------------------
```

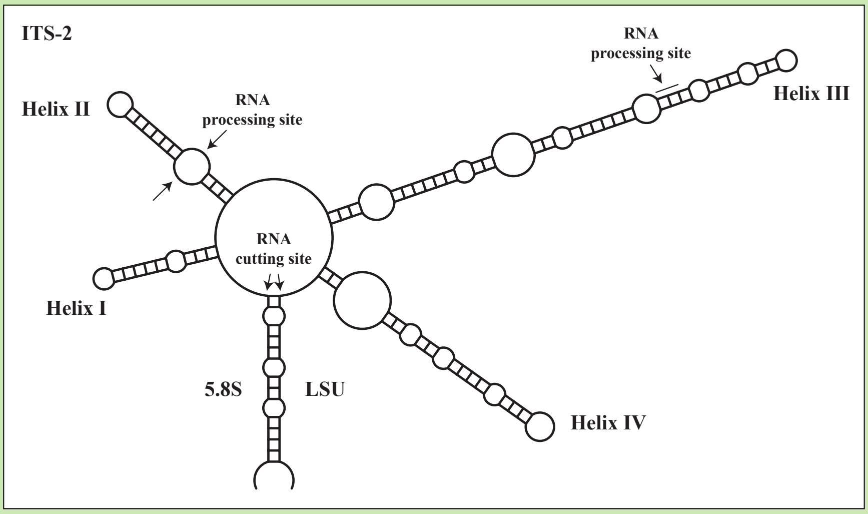

Helix I

```
1111-1
5678-9
6414-4
```

BC

```
    C      C
UCAC CCUCUC A
•||| |||||| |
GGUG GGAGAG U
    C      C
------------------------
ACC
------------------------
```

Barcode legend:

```
1 = A-U
2 = U-A
3 = G-C
4 = C-G
5 = G•U
6 = U•G
7 = mismatch
8 = deletion,single or unpaired bases
```

Helix II

```
22222222223
01234567890
63347774443
```

BC

```
    CGU       AA
UGGC   CCCGGUU  \
•|||   |||||||  |
GCCG   GGGCCAA  U
    UCU       CG
------------------------
AAGAACAGA
-------------------------------------------------------
```

Helix III

```
333333-33344444444----445-5555555--5566-666666667777
123456-78901234567----890-1234567--8901-234567890123
334288-11431338444----333-4314136--3361-363184488884
```

BC

```
    CG-       A   AUUC   A       CU     G    -       G
GGCU   AACGAGG CCC    GGG CGACAGU  GGUA GUGA CC----C U
||||   ||||||| |||    ||| ||||||•  ||•| |•|| ||    | |
CCGA   UUGCUCC GGG    CCC GCUGUCG  CCGU CGCU GG----G C
    AUG       A   ----   G       --     A    A       A
-------------------------------------------------------
GGC
---------------------------
```

Helix IV

```
   AA----   -   U     U    GU
AGG      UCG GCG UCGCU CUUU  A
|||      ||| ||| •|||| ||•|  |
UCC      AGC CGC GGCGA GAGA  A
   CCACAG   G   C     U    AA
---------------------------
UUC->
---------------------------
```

ITS-2 rRNA secondary structure model of *Coccomyxa simplex* strain CAUP H5107 (BC-3a) HG972981

Barcode 3a ITS2-C1

5.8S/LSU stem

```
---00000-000-0111-11
---12345-678-9012-34
```

BC

```
---23442-154-2453-26
```

```
GUC     C   G    C
   UGCCU AGC UCGG UU->
   ||||| |•| ||•| |•
   ACGGA UUG AGUC AG<-
AGA     C   -    C
------------------------
->CACCC
------------------------
```

Helix I

```
1111-1
5678-9
```

BC

```
6414-4
```

```
    C      C
UCAC CCUCUC A
•||| |||||| |
GGUG GGAGAG U
    C      C
------------------------
ACC
------------------------
```

Helix II

```
22222222223
01234567890
```

BC

```
63347774443
```

```
    CGU       AA
UGGC   CCCGGUU  \
•|||   |||||||  |
GCCG   GGGCCAA  U
    UCU       CG
------------------------
AAGAACAGA
-------------------------------------------------------
```

Helix III

```
333333-33344444444----445-5555555--5566-666666667777
123456-78901234567----890-1234567--8901-234567890123
```

BC

```
334288-11431338444----333-4314136--3361-363184488884
```

```
    CG-       A   AUUC   A       CU    G    -       G
GGCU   AACGAGG CCC    GGG CGACAGU  GGUA GUGA CC----C U
||||   ||||||| |||    ||| ||||||•  ||•| |•|| ||     | |
CCGA   UUGCUCC GGG    CCC GCUGUCG  CCGU CGCU GG----G C
    AUG       A   ----   G       --    A    A       A
-------------------------------------------------------
GGC
---------------------------
```

Helix IV

```
   AA----   -   U     U    GU
AGG      UCG GCG UCGCU CUUU  A
|||      ||| ||| •|||| ||•|  |
UCC      AGC CGC GGCGA GAGA  A
   CCACAG   G   C     U    AA
---------------------------
UUC->
---------------------------
```

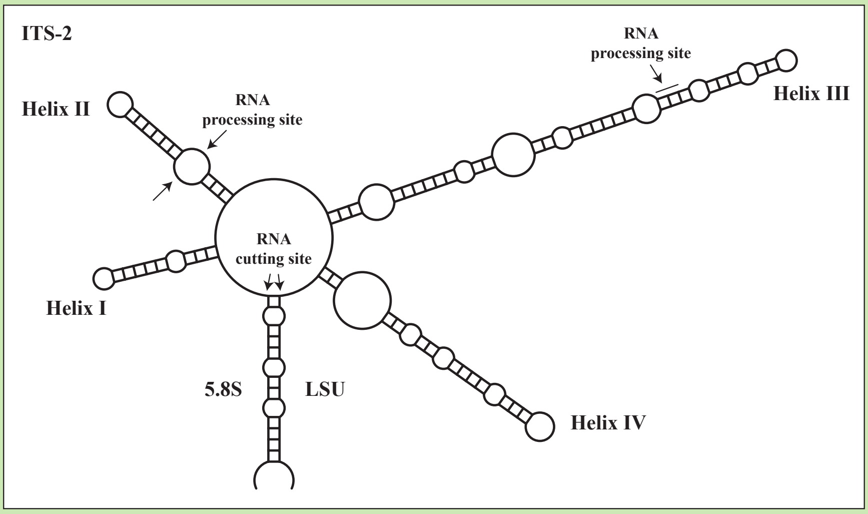


```
Barcode legend:

1 = A-U
2 = U-A
3 = G-C
4 = C-G
5 = G•U
6 = U•G
7 = mismatch
8 = deletion,single or unpaired bases
```

Barcode 3b
ITS2-C2

ITS-2 rRNA secondary structure model of *Coccomyxa simplex* strain SAG 216-2 (BC-3b) HG972989

5.8S/LSU stem

```
---00000-000-0111-11
---12345-678-9012-34
---23442-154-2453-26
```

BC

```
GUC     C   G    C
   UGCCU AGC UCGG UU->
   ||||| |•| ||•| |•
   ACGGA UUG AGUC AG<-
AGA     C   -    C
------------------------
->UACCC
------------------------
```

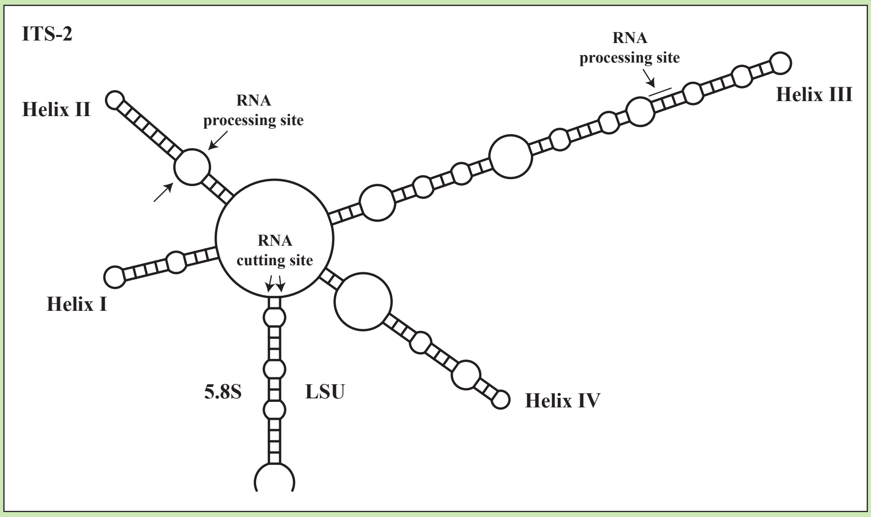

Helix I

```
1111-1
5678-9
6414-4
```

BC

```
    C     U
UCAC CCUCU U
•||| ||||| |
GGUG GGAGA C
    C     A
------------------------
ACC
------------------------
```

Barcode legend:

1 = A-U
2 = U-A
3 = G-C
4 = C-G
5 = G•U
6 = U•G
7 = mismatch
8 = deletion,single or unpaired bases

Helix II

```
22222222223
01234567890
63347774443
```

BC

```
    CGU        A
UGGC   CCCGGUUG \
•|||   |||||||• C
GCCG   GGGCCAAU /
    UCU        U
------------------------
AAGAACAGA
-------------------------------------------------------
```

Helix III

```
333333-33344444444----445-5555555--5566-666666667777
123456-78901234567----890-1234567--8901-234567890123
334288-11431338444----333-4314836--3361-363184488884
```

BC

```
    CG-       A   AAUC   A    A  CU    G    -       G
GGCU   AACGAGG CCC    GGG CGAC GU  GGUA GUGA CC----C C
||||   ||||||| |||    ||| |||| |•  ||•| |•|| ||    | |
CCGA   UUGCUCC GGG    CCC GCUG CG  CCGU CGCU GG----G C
    AUG       A   ----   G    C  --    A    A       A
-------------------------------------------------------
GGC
---------------------------
```

Helix IV

```
   AA----    -    CG   U
AGG      UCGU CGGC  CUC \
|||      |||• ||||  ||| C
UCC      AGCG GCCG  GAG /
   CCACAC    U    AA   C
---------------------------
UUC->
---------------------------
```

Barcode 3b
ITS2-C3

ITS-2 rRNA secondary structure model of *Coccomyxa simplex* strain SAG 216-3c (BC-3b) HG972990

5.8S/LSU stem

```
---00000-000-0111-11
---12345-678-9012-34
```

```
---23442-154-2453-26
```

BC

```
GUC    C   G    C
   UGCCU AGC UCGG UU->
   ||||| |•| ||•| |•
   ACGGA UUG AGUC AG<-
AGA    C   -    C
------------------------
->UACCC
------------------------
```

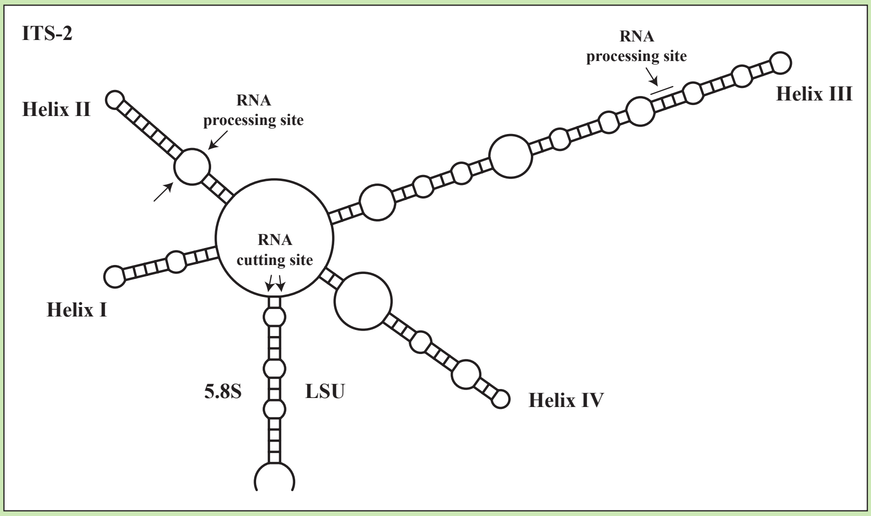

Helix I

```
1111-1
5678-9
```

```
6414-4
```

BC

```
    C     U
UCAC CCUCU U
•||| ||||| |
GGUG GGAGA C
    C     A
------------------------
ACC
------------------------
```

Helix II

```
22222222223
01234567890
```

```
63347774443
```

BC

```
    CGU         A
UGGC   CCCGGUUG \
•|||   |||||||• U
GCCG   GGGCCAAU /
    UCU         U
------------------------
AAAAACAGA
-------------------------------------------------------
```

Barcode legend:

```
1 = A-U
2 = U-A
3 = G-C
4 = C-G
5 = G•U
6 = U•G
7 = mismatch
8 = deletion,single or unpaired bases
```

Helix III

```
333333-33344444444----445-5555555--5566-666666667777
123456-78901234567----890-1234567--8901-234567890123
```

```
334288-11431338444----333-4314836--3361-363184488884
```

BC

```
    CG-       A   AAUC   A    A  CU    G    -       G
GGCU   AACGAGG CCC    GGG CGAC GU  GGUA GUGA CC----C C
||||   ||||||| |||    ||| |||| |•  ||•| |•|| ||     | |
CCGA   UUGCUCC GGG    CCC GCUG CG  CCGU CGCU GG----G C
    AUG       A   ----   G    C  --    A    A       A
-------------------------------------------------------
GGC
---------------------------
```

Helix IV

```
   AA----    -    CG   U
AGG      UCGU CGGC  CUC \
|||      |||• ||||  ||| C
UCC      AGCG GCCG  GAG /
   CCACAC    U    AA   C
---------------------------
UUC->
---------------------------
```

ITS-2 rRNA secondary structure model of *Coccomyxa simplex* strain SAG 216-8 (BC-3b) HG972991

Barcode 3b ITS2-C4

5.8S/LSU stem

```
---00000-000-0111-11
---12345-678-9012-34
---23442-154-2453-26
```

BC

```
GUC     C   G    C
   UGCCU AGC UCGG UU->
   ||||| |•| ||•| |•
   ACGGA UUG AGUC AG<-
AGA     C   -    C
-----------------------
->UACCC
-----------------------
```

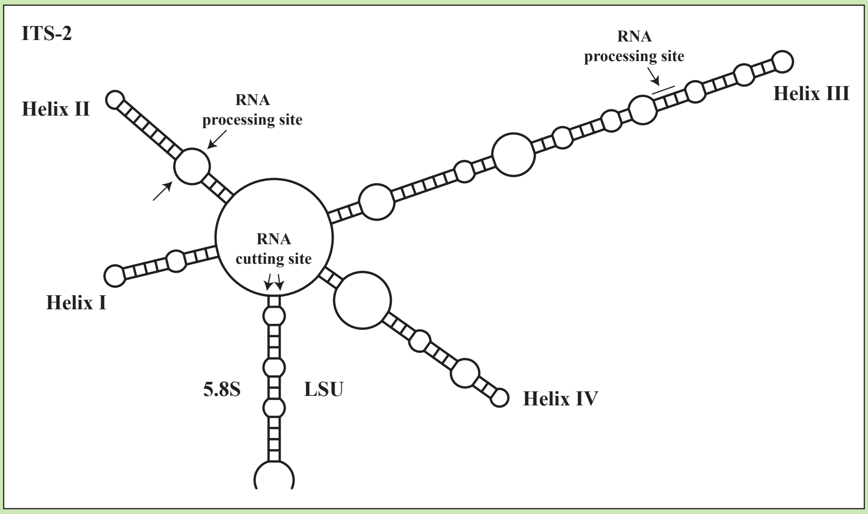

Helix I

```
1111-1
5678-9
6414-4
```

BC

```
    -     C
UCAC CCUCU U
•||| ||||| |
GGUG GGAGA C
    C     A
-----------------------
ACC
-----------------------
```

Barcode legend:

```
1 = A-U
2 = U-A
3 = G-C
4 = C-G
5 = G•U
6 = U•G
7 = mismatch
8 = deletion,single or unpaired bases
```

Helix II

```
22222222223
01234567890
63347774443
```

BC

```
    CGU        A
UGGC   CCCGGUUA \
•|||   |||||||| U
GCCG   GGGCCAAU /
    UCU        U
-----------------------
AAGAACAGA
-----------------------------------------------------
```

Helix III

```
333333-33344444444----445-5555555--5566-666666667777
123456-78901234567----890-1234567--8901-234567890123
334288-11431338444----333-4314836--3361-363184488884
```

BC

```
    CG-       A   AAUC   A    A  CU    G    -       G
GGCU   AACGAGG CCC    GGG CGAC GU  GGUA GUGA CC----C C
||||   ||||||| |||    ||| |||| |•  ||•| |•|| ||    | |
CCGA   UUGCUCC GGG    CCC GCUG CG  CCGU CGCU GG----G C
    AUG       A   ----   G    C  --    A    A       A
-----------------------------------------------------
GGC
--------------------------
```

Helix IV

```
   AA----    -    CG   U
AGG      UCGU CGGC  CUC \
|||      |||• ||||  ||| C
UCC      AGCG GCCG  GAG /
   CCACAC    U    AA   C
--------------------------
UUC->
--------------------------
```

ITS-2 rRNA secondary structure model of *Coccomyxa simplex* strain SAG 216-9a (BC-3b) FN298926

Barcode 3b ITS2-C4

5.8S/LSU stem

```
---00000-000-0111-11
---12345-678-9012-34
---23442-154-2453-26
```

BC

```
GUC     C   G    C
   UGCCU AGC UCGG UU->
   ||||| |•| ||•| |•
   ACGGA UUG AGUC AG<-
AGA     C   -    C
------------------------
->UACCC
------------------------
```

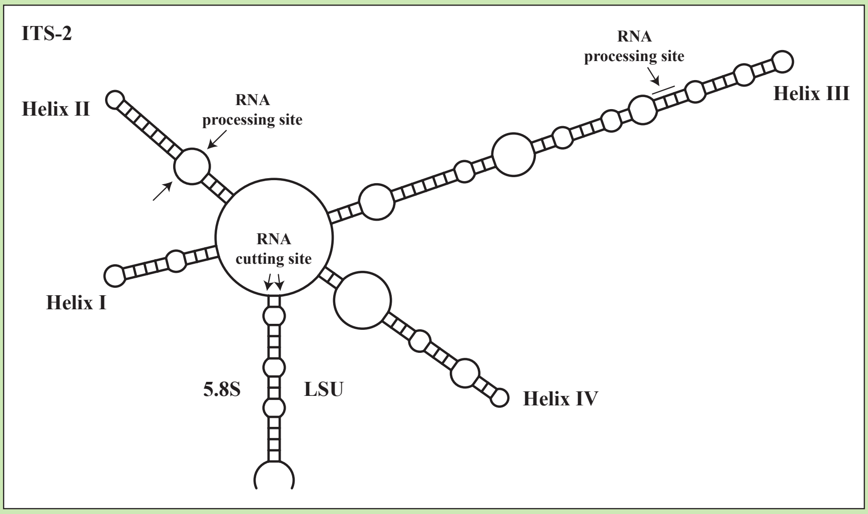

Helix I

```
1111-1
5678-9
6414-4
```

BC

```
    -     C
UCAC CCUCU U
•||| ||||| |
GGUG GGAGA C
    C     A
------------------------
ACC
------------------------
```

Barcode legend:

1 = A-U
2 = U-A
3 = G-C
4 = C-G
5 = G•U
6 = U•G
7 = mismatch
8 = deletion,single or unpaired bases

Helix II

```
22222222223
01234567890
63347774443
```

BC

```
    CGU        A
UGGC   CCCGGUUA \
•|||   |||||||| U
GCCG   GGGCCAAU /
    UCU        U
------------------------
AAGAACAGA
-------------------------------------------------------
```

Helix III

```
333333-33344444444----445-5555555--5566-666666667777
123456-78901234567----890-1234567--8901-234567890123
334288-11431338444----333-4314836--3361-363184488884
```

BC

```
    CG-       A   AAUC   A    A  CU    G    -       G
GGCU   AACGAGG CCC    GGG CGAC GU  GGUA GUGA CC----C C
||||   ||||||| |||    ||| |||| |•  ||•| |•|| ||    | |
CCGA   UUGCUCC GGG    CCC GCUG CG  CCGU CGCU GG----G C
    AUG       A   ----   G    C  --    A    A       A
-------------------------------------------------------
GGC
---------------------------
```

Helix IV

```
   AA----    -    CG   U
AGG      UCGU CGGC  CUC \
|||      |||• ||||  ||| C
UCC      AGCG GCCG  GAG /
   CCACAC    U    AA   C
---------------------------
UUC->
---------------------------
```

Barcode 1b
ITS2-C5

ITS-2 rRNA secondary structure model of *Coccomyxa simplex* strain SAG 216-5 (BC-3c) HG972982

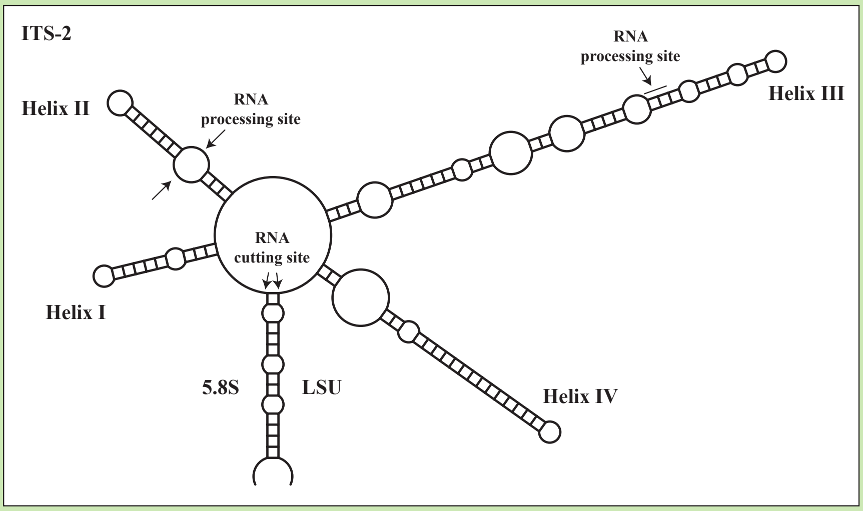

5.8S/LSU stem

```
---00000-000-0111-11
---12345-678-9012-34
---23442-154-2453-26
```

BC

```
GUC      C    G     C
   UGCCU AGC UCGG UU->
   ||||| |•| ||•| |•
   ACGGA UUG AGUC AG<-
AGA      C    -     C
------------------------
->CACCC
------------------------
```

Helix I

```
1111-1
5678-9
6414-4
```

BC

```
    C      C
UCAC CCUCUC A
•||| |||||| |
GGUG GGAGAG C
    C      C
------------------------
ACC
------------------------
```

Helix II

```
22222222223
01234567890
63347774443
```

BC

```
    CGU       AA
UGGC   CCCGGUU  \
•|||   |||||||  U
GCCG   GGGCCAA  /
    UCU       CG
------------------------
AAGAACAGA
-------------------------------------------------------
```

Helix III

```
333333-333444444444---4555---55555--5566-666666667777
123456-789012345678---9012---34567--8901-234567890123
334288-114313384448---3383---14136--3361-363184488884
```

BC

```
    CG-       A   AAUC    ACA     CU    G    -      G
GGCU   AACGAGG CCC    GG-G   ACAGU  GGUA GUGA CC----C U
||||   ||||||| |||    || |   ||||•  ||•| |•|| ||    | |
CCGA   UUGCUCC GGG    CC-C   UGUCG  CCGU CGCU GG----G C
    AUG       A   ----    GGC     --    A    A      A
-------------------------------------------------------
GGC
---------------------------
```

Helix IV

```
   AA----   -              U
AGG      UCG GCGGUCGCUUUUUUU A
|•|      ||| ||||•|||||••|||| |
UUC      AGC CGCCGGCGAGGAAAA G
   CCACAC   A              A
---------------------------
UUC->
---------------------------
```

Barcode legend:

1 = A-U
2 = U-A
3 = G-C
4 = C-G
5 = G•U
6 = U•G
7 = mismatch
8 = deletion,single or unpaired bases

ITS-2 rRNA secondary structure model of *Coccomyxa simplex* strain SAG 216-10 (BC-3c) HG972986

Barcode 3c ITS2-C5

5.8S/LSU stem

```
---00000-000-0111-11
---12345-678-9012-34
---23442-154-2453-26
```

BC

```
GUC      C    G     C
   UGCCU  AGC  UCGG  UU->
   |||||  |•|  ||•|  |•
   ACGGA  UUG  AGUC  AG<-
AGA      C    -     C
------------------------
->CACCC
------------------------
```

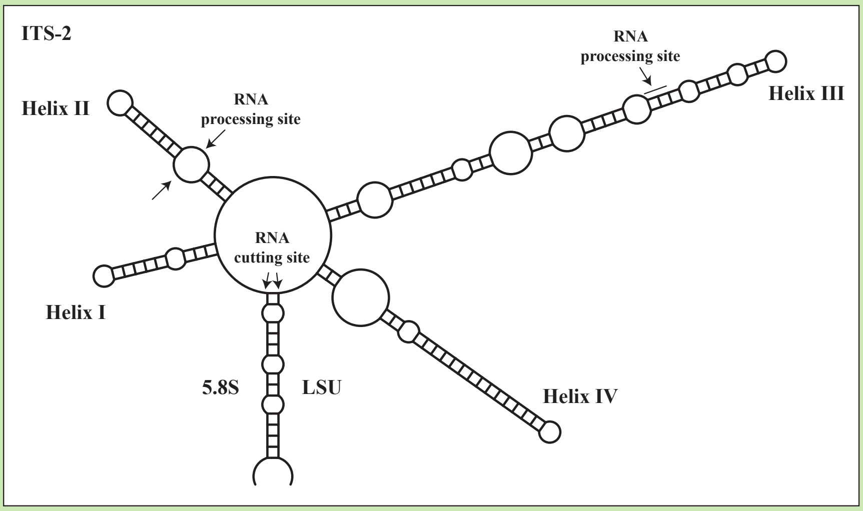

Helix I

```
1111-1
5678-9
6414-4
```

BC

```
    C      C
UCAC CCUCUC A
•||| |||||| |
GGUG GGAGAG C
    C      C
------------------------
ACC
------------------------
```

Helix II

```
22222222223
01234567890
63347774443
```

BC

```
    CGU       AA
UGGC   CCCGGUU  \
•|||   |||||||  U
GCCG   GGGCCAA  /
    UCU       CG
------------------------
AAGAACAGA
-------------------------------------------------------
```

Helix III

```
333333-333444444444---4555---55555--5566-666666667777
123456-789012345678---9012---34567--8901-234567890123
334288-114313384448---3383---14136--3361-363184488884
```

BC

```
    CG-       A   AAUC    ACA     CU    G    -       G
GGCU   AACGAGG CCC    GG-G   ACAGU  GGUA GUGA CC----C U
||||   ||||||| |||    || |   ||||•  ||•| |•|| ||     | |
CCGA   UUGCUCC GGG    CC-C   UGUCG  CCGU CGCU GG----G C
    AUG       A   ----    GGC     --    A    A       A
-------------------------------------------------------
GGC
---------------------------
```

Helix IV

```
   AA----   -              U
AGG      UCG GCGGUCGCUUUUUUU A
|•|      ||| ||||•||||••|||| |
UUC      AGC CGCCGGCGAGGAAAA G
   CCACAC   A              A
---------------------------
UUC->
---------------------------
```

Barcode legend:

1 = A-U
2 = U-A
3 = G-C
4 = C-G
5 = G•U
6 = U•G
7 = mismatch
8 = deletion,single or unpaired bases

ITS-2 rRNA secondary structure model of *Coccomyxa simplex* strain SAG 216-12 (BC-3c) HG972987

Barcode 3c
ITS2-C5

5.8S/LSU stem

```
---00000-000-0111-11
---12345-678-9012-34
---23442-154-2453-26
```

BC

```
GUC      C   G    C
   UGCCU AGC UCGG UU->
   ||||| |•| ||•| |•
   ACGGA UUG AGUC AG<-
AGA      C   -    C
------------------------
->CACCC
------------------------
```

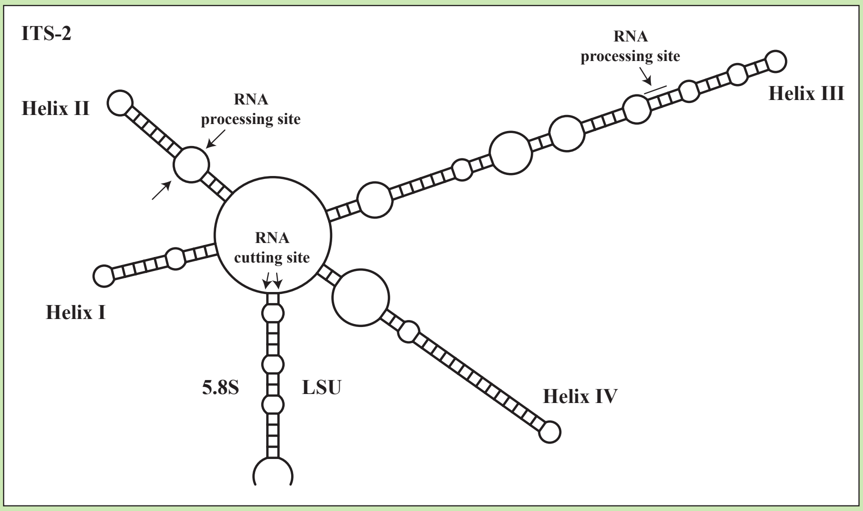

Helix I

```
1111-1
5678-9
6414-4
```

BC

```
    C      C
UCAC CCUCUC A
•||| |||||| |
GGUG GGAGAG C
    C      C
------------------------
ACC
------------------------
```

Helix II

```
22222222223
01234567890
63347774443
```

BC

```
    CGU       AA
UGGC   CCCGGUU  \
•|||   |||||||  U
GCCG   GGGCCAA  /
    UCU       CG
------------------------
AAGAACAGA
-------------------------------------------------------
```

Helix III

```
333333-333444444444---4555---55555--5566-666666667777
123456-789012345678---9012---34567--8901-234567890123
334288-114313384448---3383---14136--3361-363184488884
```

BC

```
    CG-       A   AAUC    ACA     CU    G    -       G
GGCU   AACGAGG CCC    GG-G   ACAGU  GGUA GUGA CC----C U
||||   ||||||| |||    || |   ||||•  ||•| |•|| ||    | |
CCGA   UUGCUCC GGG    CC-C   UGUCG  CCGU CGCU GG----G C
    AUG       A   ----    GGC     --    A    A       A
-------------------------------------------------------
GGC
---------------------------
```

Helix IV

```
   AA----   -               U
AGG      UCG GCGGUCGCUUUUUUU A
|•|      ||| ||||•||||••|||| |
UUC      AGC CGCCGGCGAGGAAAA G
   CCACAC  A               A
---------------------------
UUC->
---------------------------
```

Barcode legend:

```
1 = A-U
2 = U-A
3 = G-C
4 = C-G
5 = G•U
6 = U•G
7 = mismatch
8 = deletion,single or unpaired bases
```

ITS-2 rRNA secondary structure model of *Coccomyxa simplex* strain CCAP 216/15 (BC-3c) HG972985

Barcode 3c
ITS2-C5

5.8S/LSU stem

```
---00000-000-0111-11
---12345-678-9012-34
---23442-154-2453-26
```

BC

```
GUC     C   G    C
   UGCCU AGC UCGG UU->
   ||||| |•| ||•| |•
   ACGGA UUG AGUC AG<-
AGA     C   -    C
------------------------
->CACCC
------------------------
```

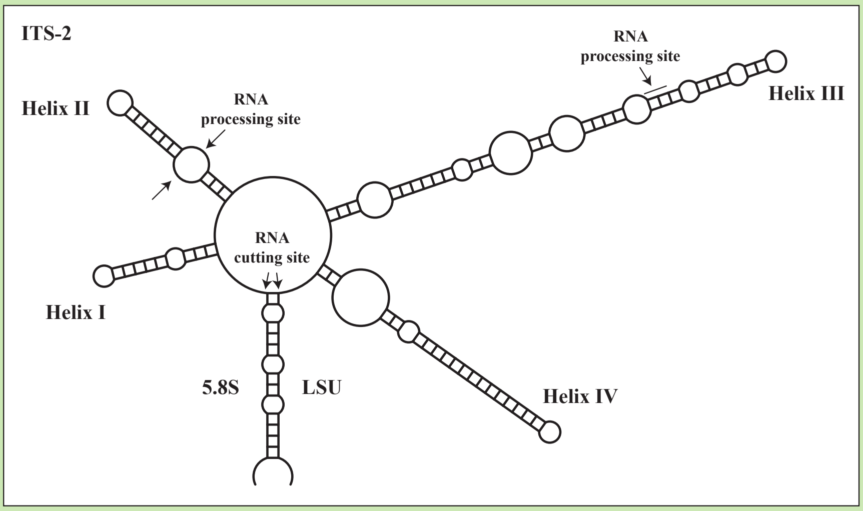

Helix I

```
1111-1
5678-9
6414-4
```

BC

```
    C      C
UCAC CCUCUC A
•||| |||||| |
GGUG GGAGAG C
    C      C
------------------------
ACC
------------------------
```

Helix II

```
22222222223
01234567890
63347774443
```

BC

```
    CGU       AA
UGGC   CCCGGUU  \
•|||   |||||||  U
GCCG   GGGCCAA  /
    UCU       CG
------------------------
AAGAACAGA
-------------------------------------------------------
```

Barcode legend:

1 = A-U
2 = U-A
3 = G-C
4 = C-G
5 = G•U
6 = U•G
7 = mismatch
8 = deletion,single or unpaired bases

Helix III

```
333333-333444444444---4555---55555--5566-666666667777
123456-789012345678---9012---34567--8901-234567890123
334288-114313384448---3383---14136--3361-363184488884
```

BC

```
    CG-       A   AAUC    ACA     CU    G    -       G
GGCU   AACGAGG CCC    GG-G   ACAGU  GGUA GUGA CC----C U
||||   ||||||| |||    || |   ||||•  ||•| |•|| ||     | |
CCGA   UUGCUCC GGG    CC-C   UGUCG  CCGU CGCU GG----G C
    AUG       A   ----    GGC     --    A    A       A
-------------------------------------------------------
GGC
---------------------------
```

Helix IV

```
   AA----   -              U
AGG      UCG GCGGUCGCUUUUUUU A
|•|      ||| ||||•|||||••|||| |
UUC      AGC CGCCGGCGAGGAAAA G
   CCACAC   A              A
---------------------------
UUC->
---------------------------
```

ITS-2 rRNA secondary structure model of *Coccomyxa simplex* strain SAG 216-11a (BC-3c) HG972983

Barcode 3c ITS2-C6

5.8S/LSU stem

```
---00000-000-0111-11
---12345-678-9012-34
---23442-154-2453-26
```

BC

```
GUC     C   G    C
   UGCCU AGC UCGG UU->
   ||||| |•| ||•| |•
   ACGGA UUG AGUC AG<-
AGA     C   -    C
------------------------
->CACCC
------------------------
```

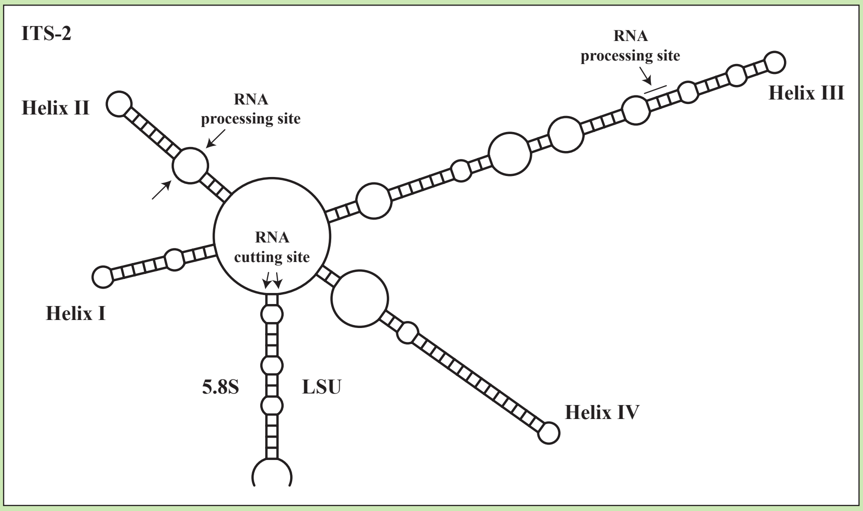

Helix I

```
1111-1
5678-9
6414-4
```

BC

```
    C      C
UCAC CCUCUC A
•||| |||||| |
GGUG GGAGAG C
    C      C
------------------------
ACC
------------------------
```

Barcode legend:

```
1 = A-U
2 = U-A
3 = G-C
4 = C-G
5 = G•U
6 = U•G
7 = mismatch
8 = deletion,single or unpaired bases
```

Helix II

```
22222222223
01234567890
63347774443
```

BC

```
    CGU       AA
UGGC   CCCGGUU  \
•|||   |||||||  U
GCCG   GGGCCAA  /
    UCU       CG
------------------------
AAGAACAGA
-------------------------------------------------------
```

Helix III

```
333333-333444444444---4555---55555--5566-666666667777
123456-789012345678---9012---34567--8901-234567890123
334288-114313384448---3383---14136--3361-363184488884
```

BC

```
    CG-       A   AAUC    ACA     CU    G    -       G
GGCU   AACGAGG CCC    GG-G   ACAGU  GGUA GUGA CC----C U
||||   ||||||| |||    || |   ||||•  ||•| |•|| ||    | |
CCGA   UUGCUCC GGG    CC-C   UGUCG  CCGU CGCU GG----G C
    AUG       A   ----    GGC     --    A    A       A
-------------------------------------------------------
GGC
---------------------------
```

Helix IV

```
   AA----   -               U
AGG      UCG GCGGUCGCUUUUUUU A
|•|      ||| ||||•||||••|||| |
UUC      AGC CGCCGGCGAGGAAAA G
   CCACAC   A               G
---------------------------
UUC->
---------------------------
```

ITS-2 rRNA secondary structure model of *Coccomyxa simplex* strain SAG 216-11b (BC-3c) HG972984

Barcode 3c
ITS2-C6

5.8S/LSU stem

```
---00000-000-0111-11
---12345-678-9012-34
---23442-154-2453-26
```

BC

```
GUC     C   G    C
   UGCCU AGC UCGG UU->
   |||||  |•|  ||•|  |•
   ACGGA UUG AGUC AG<-
AGA     C   -    C
------------------------
->CACCC
------------------------
```

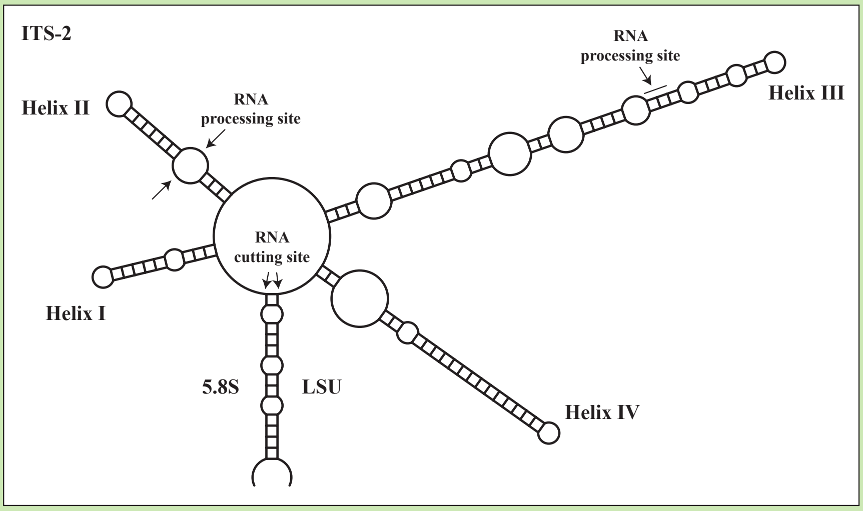

Helix I

```
1111-1
5678-9
6414-4
```

BC

```
    C      C
UCAC CCUCUC A
•||| |||||| |
GGUG GGAGAG C
    C      C
------------------------
ACC
------------------------
```

Barcode legend:

```
1 = A-U
2 = U-A
3 = G-C
4 = C-G
5 = G•U
6 = U•G
7 = mismatch
8 = deletion,single or unpaired bases
```

Helix II

```
22222222223
01234567890
63347774443
```

BC

```
    CGU       AA
UGGC   CCCGGUU  \
•|||   |||||||  U
GCCG   GGGCCAA  /
    UCU       CG
------------------------
AAGAACAGA
-------------------------------------------------------
```

Helix III

```
333333-333444444444---4555---55555--5566-66666666777 7
123456-789012345678---9012---34567--8901-23456789012 3
334288-114313384448---3383---14136--3361-36318448888 4
```

```
    CG-       A   AAUC    ACA     CU    G    -       G
GGCU   AACGAGG CCC    GG-G   ACAGU  GGUA GUGA CC----C U
||||   ||||||| |||    || |   ||||•  ||•| |•|| ||    | |
CCGA   UUGCUCC GGG    CC-C   UGUCG  CCGU CGCU GG----G C
    AUG       A   ----    GGC     --    A    A       A
-------------------------------------------------------
GGC
---------------------------
```

BC

Helix IV

```
   AA----   -               U
AGG      UCG GCGGUCGCUUUUUUU A
|•|      ||| ||||•||||••|||| |
UUC      AGC CGCCGGCGAGGAAAA G
   CCACAC   A               G
---------------------------
UUC->
---------------------------
```

ITS-2 rRNA secondary structure model of *Coccomyxa simplex* strain SAG 216-6 (BC-3c) HG972988

Barcode 3c ITS2-C6

5.8S/LSU stem

```
---00000-000-0111-11
---12345-678-9012-34
---23442-154-2453-26
```

BC

```
GUC      C    G     C
   UGCCU AGC UCGG UU->
   ||||| |•| ||•| |•
   ACGGA UUG AGUC AG<-
AGA      C    -     C
------------------------
->CACCC
------------------------
```

Helix I

```
1111-1
5678-9
6414-4
```

BC

```
    C      C
UCAC CCUCUC A
•||| |||||| |
GGUG GGAGAG C
    C      C
------------------------
ACC
------------------------
```

Helix II

```
22222222223
01234567890
63347774443
```

BC

```
    CGU        AA
UGGC   CCCGGUU  \
•|||   |||||||  U
GCCG   GGGCCAA  /
    UCU        CG
------------------------
AAGAACAGA
-------------------------------------------------------
```

Helix III

```
333333-333444444444---4555---55555--5566-666666667777
123456-789012345678---9012---34567--8901-234567890123
334288-114313384448---3383---14136--3361-363184488884
```

BC

```
    CG-        A   AAUC    ACA     CU    G    -       G
GGCU   AACGAGG CCC     GG-G   ACAGU  GGUA GUGA CC----C U
||||   ||||||| |||     || |   ||||•  ||•| |•|| ||     | |
CCGA   UUGCUCC GGG     CC-C   UGUCG  CCGU CGCU GG----G C
    AUG        A   ----    GGC     --    A    A       A
-------------------------------------------------------
GGC
---------------------------
```

Helix IV

```
   AA----   -                U
AGG      UCG GCGGUCGCUUUUUUU A
|•|      ||| ||||•|||||••||| |
UUC      AGC CGCCGGCGAGGAAAA G
   CCACAC   A                G
---------------------------
UUC->
---------------------------
```

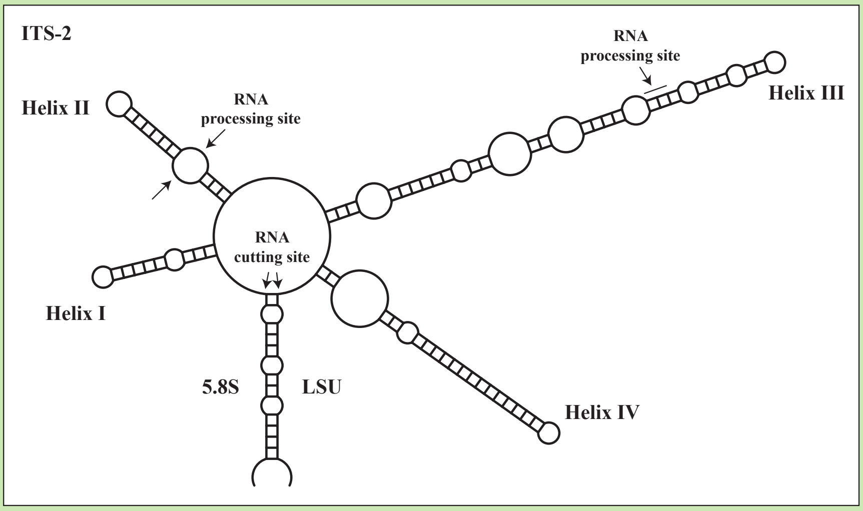


```
Barcode legend:

1 = A-U
2 = U-A
3 = G-C
4 = C-G
5 = G•U
6 = U•G
7 = mismatch
8 = deletion,single or unpaired bases
```

ITS-2 rRNA secondary structure model of *Coccomyxa simplex* strain CCAP 216/24 (BC-3c) FN298927

Barcode 3c
ITS2-C7

5.8S/LSU stem

```
---00000-000-0111-11
---12345-678-9012-34
```

```
---23442-154-2453-26
```

BC

```
GUC     C   G    C
   UGCCU AGC UCGG UU->
   ||||| |•| ||•| |•
   ACGGA UUG AGUC AG<-
AGA     C   -    C
------------------------
->CACCC
------------------------
```

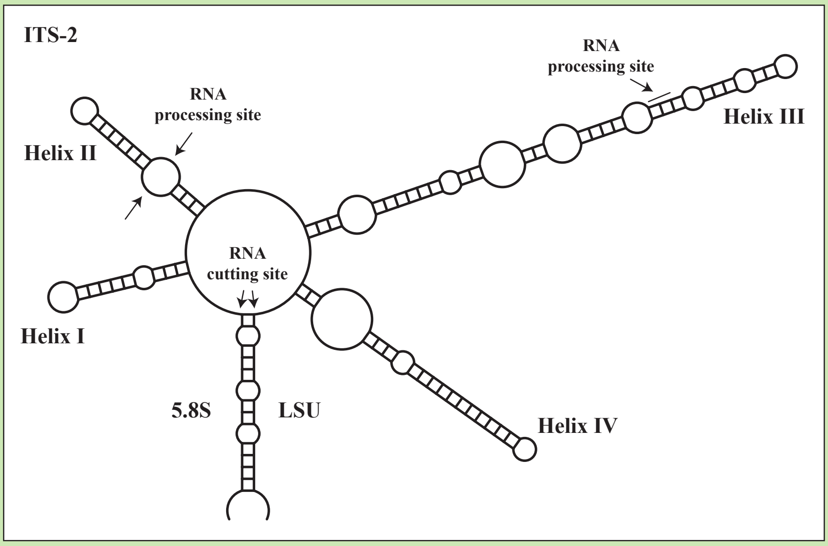

Helix I

```
1111-1
5678-9
```

```
6414-4
```

BC

```
    C      UU
UCAC CCUCCU  A
•||| |||||•  |
GGUG GGAGGG  C
    C      CA
------------------------
ACC
------------------------
```

Barcode legend:

```
1 = A-U
2 = U-A
3 = G-C
4 = C-G
5 = G•U
6 = U•G
7 = mismatch
8 = deletion,single or unpaired bases
```

Helix II

```
22222222223
01234567890
```

```
63347774443
```

BC

```
    CGU       GA
UGGC   CCCGGUU  \
•|||   ||||||•  U
GCCG   GGGCCAG  /
    UCU       UC
------------------------
AAGAACAGA
-------------------------------------------------------
```

Helix III

```
333333-333444444444---4555---55555--5566-666666667777
123456-789012345678---9012---34567--8901-234567890123
```

```
334288-114313384448---3383---14136--3361-363184488884
```

BC

```
    CG-       A   GAUC    ACA     CU    G    -       G
GGCU   AACGAGG CCC    GG-G   ACAGU  GGUA GUGA CC----C U
||||   ||||||| |||    || |   ||||•  ||•| |•|| ||    | |
CCGA   UUGCUCC GGG    CC-C   UGUCG  CCGU CGCU GG----G C
    AUG       A   ----    GGC     --    A    A       A
-------------------------------------------------------
GGC
---------------------------
```

Helix IV

```
   AA----    -            C
AGG      UCGC CGGUCUUUCUCU A
|•|      |||| |||•|||||•|| |
UUC      AGCG GCCGGAAAGGGA A
   CCACAC    C            C
---------------------------
UUC->
---------------------------
```

ITS-2 rRNA secondary structure model of *Coccomyxa simplex* strain CCAP 812/2A (BC-3c) HG972992

Barcode 3c ITS2-C7

5.8S/LSU stem

```
---00000-000-0111-11
---12345-678-9012-34
---23442-154-2453-26   BC

GUC     C   G    C
   UGCCU AGC UCGG UU->
   ||||| |•| ||•| |•
   ACGGA UUG AGUC AG<-
AGA     C   -    C
------------------------
->CACCC
------------------------
```

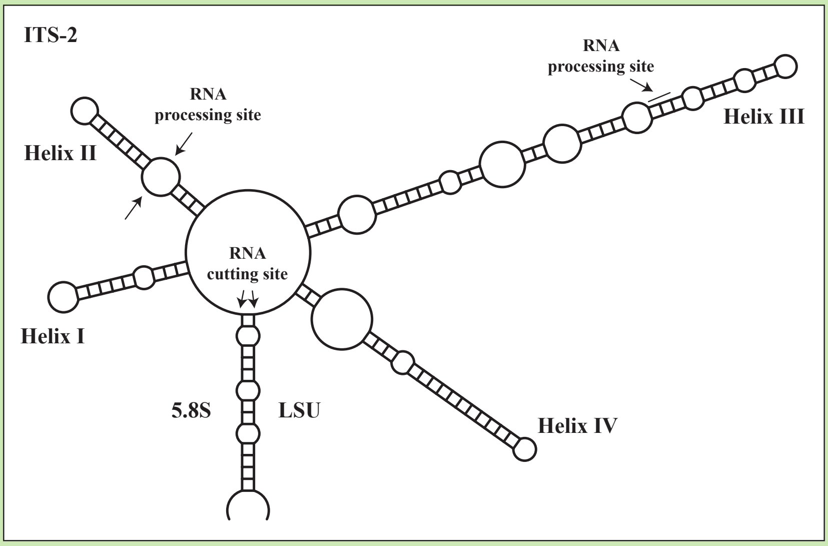

Helix I

```
1111-1
5678-9
6414-4   BC

    C      UU
UCAC CCUCCU  A
•||| |||||•  |
GGUG GGAGGG  C
    C      CA
------------------------
ACC
------------------------
```

Barcode legend:

```
1 = A-U
2 = U-A
3 = G-C
4 = C-G
5 = G•U
6 = U•G
7 = mismatch
8 = deletion,single or unpaired bases
```

Helix II

```
22222222223
01234567890
63347774443   BC

    CGU       GA
UGGC   CCCGGUU  \
•|||   ||||||•  U
GCCG   GGGCCAG  /
    UCU       UC
------------------------
AAGAACAGA
-------------------------------------------------------
```

Helix III

```
333333-333444444444---4555---55555--5566-666666667777
123456-789012345678---9012---34567--8901-234567890123
334288-114313384448---3383---14136--3361-363184488884   BC

    CG-       A   GAUC    ACA     CU    G    -       G
GGCU   AACGAGG CCC    GG-G   ACAGU  GGUA GUGA CC----C U
||||   ||||||| |||    || |   ||||•  ||•| |•|| ||     | |
CCGA   UUGCUCC GGG    CC-C   UGUCG  CCGU CGCU GG----G C
    AUG       A   ----    GGC     --    A    A       A
-------------------------------------------------------
GGC
---------------------------
```

Helix IV

```
   AA----    -            C
AGG      UCGC CGGUCUUUCUCU A
|•|      |||| |||•|||||•|| |
UUC      AGCG GCCGGAAAGGGA A
   CCACAC    C            C
---------------------------
UUC->
---------------------------
```

ITS-2 rRNA secondary structure model of *Coccomyxa simplex* strain CCAP 812/2B (BC-3c) HG972993

Barcode 3c
ITS2-C7

5.8S/LSU stem

```
---00000-000-0111-11
---12345-678-9012-34
---23442-154-2453-26
```

BC

```
GUC     C   G    C
   UGCCU AGC UCGG UU->
   ||||| |•| ||•| |•
   ACGGA UUG AGUC AG<-
AGA     C   -    C
------------------------
->CACCC
------------------------
```

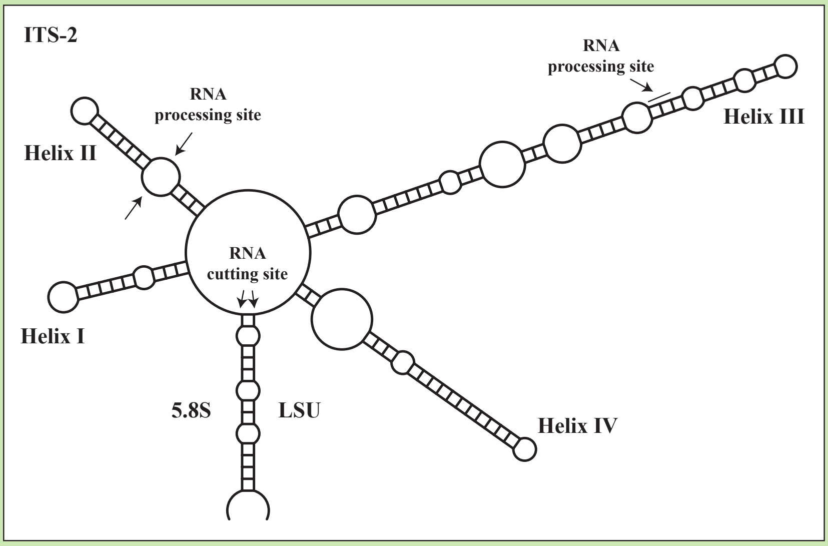

Helix I

```
1111-1
5678-9
6414-4
```

BC

```
    C     UU
UCAC CCUCCU  A
•||| |||||•  |
GGUG GGAGGG  C
    C     CA
------------------------
ACC
------------------------
```

Barcode legend:

1 = A-U
2 = U-A
3 = G-C
4 = C-G
5 = G•U
6 = U•G
7 = mismatch
8 = deletion,single or unpaired bases

Helix II

```
22222222223
01234567890
63347774443
```

BC

```
    CGU       GA
UGGC   CCCGGUU  \
•|||   ||||||•  U
GCCG   GGGCCAG  /
    UCU       UC
------------------------
AAGAACAGA
-------------------------------------------------------
```

Helix III

```
333333-333444444444---4555---55555--5566-666666667777
123456-789012345678---9012---34567--8901-234567890123
334288-114313384448---3383---14136--3361-363184488884
```

BC

```
    CG-       A   GAUC    ACA     CU    G    -       G
GGCU   AACGAGG CCC    GG-G   ACAGU  GGUA GUGA CC----C U
||||   ||||||| |||    || |   ||||•  ||•| |•|| ||    | |
CCGA   UUGCUCC GGG    CC-C   UGUCG  CCGU CGCU GG----G C
    AUG       A   ----    GGC     --    A    A       A
-------------------------------------------------------
GGC
---------------------------
```

Helix IV

```
   AA----    -            C
AGG      UCGC CGGUCUUUCUCU A
|•|      |||| |||•|||||•|| |
UUC      AGCG GCCGGAAAGGGA A
   CCACAC    C            C
---------------------------
UUC->
---------------------------
```

ITS-2 rRNA secondary structure model of *Coccomyxa vinatzeri* strain ASIB V16 (BC-4) HG972994

Barcode 4 ITS2-E

5.8S/LSU stem

```
---00000-000-0111-11
---12345-678-9012-34
---23442-154-2453-26
```

BC

```
GUC     C   G    C
   UGCCU AGC UCGG UU->
   ||||| |•| ||•| |•
   ACGGA UUG AGUC AG<-
AGA     C   -    C
------------------------
->UACCC
------------------------
```

Helix I

```
1111-1
5678-9
6414-2
```

BC

```
    C      -    UU
UCAC UUUCCU AUUC  U
•||| |•|||• ||||  |
GGUG AGAGGG UAAG  G
    -      C    UU
------------------------
AUC
------------------------
```

Helix II

```
22222222223
01234567890
65347774443
```

BC

```
    AGU        G
UGGC   CCCGGUUA G
••||   |||||•|| |
GUCG   GGGCCGAU A
    CCU        A
------------------------
AAGACCAGA
-------------------------------------------------------
```

Helix III

```
333333-333444444444---4555---55555--5566-6666-66667777
123456-789012345678---9012---34567--8901-2345-67890123
334288-114313384448---3383---14136--3361-3635-14443226
```

BC

```
    CG-       A   AAUC    ACA     CU     G    -        GU
GGCU   AACGAGG CCC    GG-G   ACAGU  GGUA GUGG ACCCGUUU  \
||||   ||||||| |||    || |   ||||•  ||•| |•|• |||||||•  U
CCGA   UUGCUCC GGG    CC-C   UGUCG  CCGU CGCU UGGGCAAG  /
    AUG       A   ----    GGC     --     A    G        UA
-------------------------------------------------------
GGCA
----------------------------
```

Helix IV

```
  AA       -   -   U
GG UCGUUUG GUG GUG G
|| ||||||| ||| |•| |
CC AGCAAAC CAC CGC U
  --       A   A   U
----------------------------
AAAUAAUAUUUC->
----------------------------
```

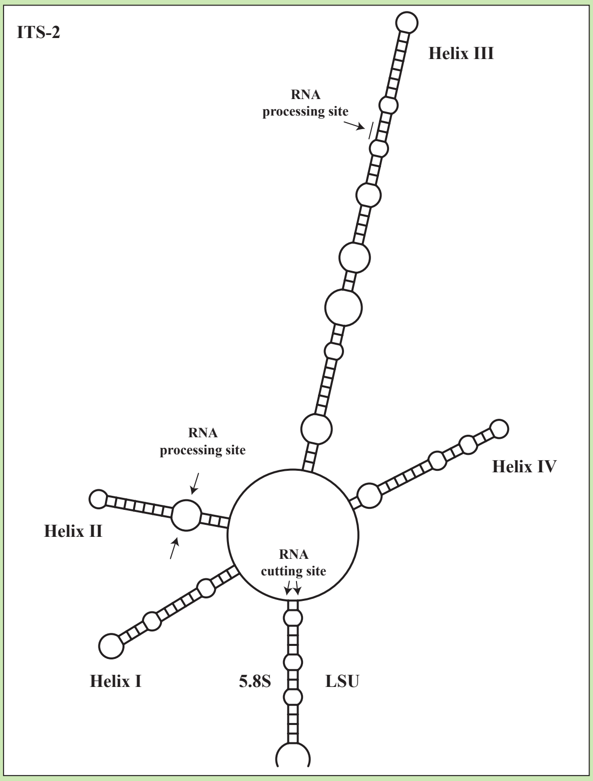

Barcode legend:

1 = A-U
2 = U-A
3 = G-C
4 = C-G
5 = G•U
6 = U•G
7 = mismatch
8 = deletion,single or unpaired bases

ITS-2 rRNA secondary structure model of *Coccomyxa galuniae* strain CCAP 211/97 (BC-5) FN298928

Barcode 5
ITS2-E

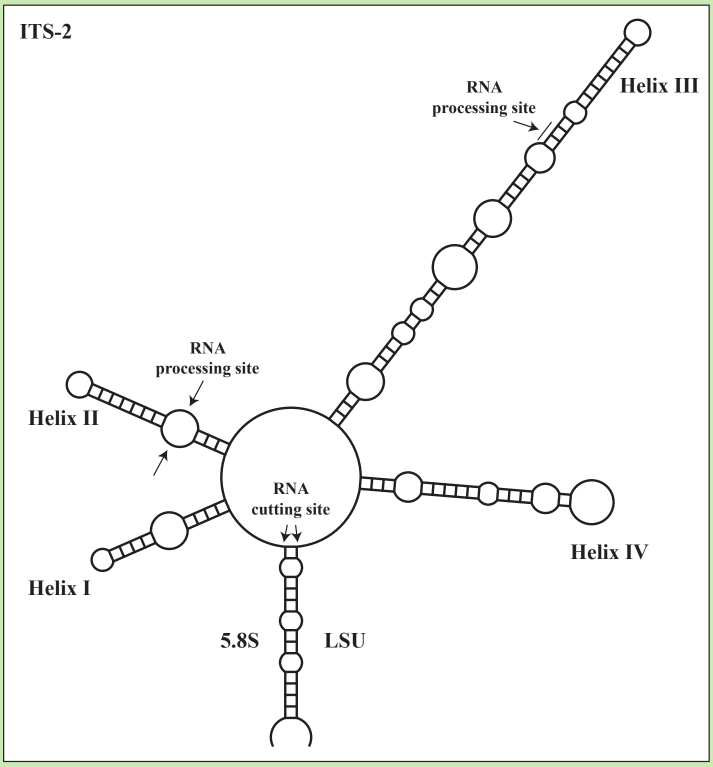

5.8S/LSU stem

```
---00000-000-0111-11
---12345-678-9012-34
---23442-154-2453-26
```
BC

```
GUC     C   G    C
   UGCCU AGC UCGG UU->
   ||||| |•| ||•| |•
   ACGGA UUG AGUC AG<-
AGA     C   -    C
------------------------
->CACCC
------------------------
```

Helix I

```
11111
56789
64141
```
BC

```
     CA-     U
UCACA   CCCGC C
•||||   ||||| |
GGUGU   GGGCG G
     AGC     A
------------------------
AUC
------------------------
```

Helix II

```
22222222223
01234567890
65347774443
```
BC

```
    AGU        CA
UGGC   CCCGGUUC  \
••||   ||||||||  U
GUCG   GGGCCAAG  /
    CCU        AC
------------------------
AAGACCAGA
-------------------------------------------------------
```

```
Barcode legend:

1 = A-U
2 = U-A
3 = G-C
4 = C-G
5 = G•U
6 = U•G
7 = mismatch
8 = deletion,single or unpaired bases
```

Helix III

```
333333-333444444444---4555---55555--5566-666666667777
123456-789012345678---9012---34567--8901-234567890123
334288-134383384448---3383---14136--3361-363226448888
```
BC

```
    CG-    A  A   GAUC    ACA     CU    G        CC
GGCU   AGCG GG CCC    GG-G   ACAGU  GGUA GUGUUUCC  \
||||   |||| || |||    || |   ||||•  ||•| |•|||•||  A
CCGA   UCGC CC GGG    CC-C   UGUCG  CCGU CGCAAGGG  /
    AUG    C  A   ----    GGC     --    A        CC
-------------------------------------------------------
GGCA
---------------------------
```

Helix IV

```
    U-       -   U-  GCCG
GGAA  UUUGGUU UCG  GC    U
|•||  ||||||• |||  ||    |
CUUU  AAACCAG AGC  CG    G
    CC       G   UC  AAAA
---------------------------
AACAUUC->
---------------------------
```

ITS-2 rRNA secondary structure model of *Coccomyxa galuniae* strain CCAP 812/5 (BC-5) HG972995

Barcode 5 ITS2-E

5.8S/LSU stem

```
---00000-000-0111-11
---12345-678-9012-34
---23442-154-2453-26
```

BC

```
GUC      C   G    C
   UGCCU AGC UCGG UU->
   ||||| |•| ||•| |•
   ACGGA UUG AGUC AG<-
AGA      C   -    C
------------------------
->CACCC
------------------------
```

Helix I

```
11111
56789
64141
```

BC

```
     CA-     U
UCACA   CCCGC C
•||||   ||||| |
GGUGU   GGGCG G
     AGC     A
------------------------
AUC
------------------------
```

Helix II

```
22222222223
01234567890
65347774443
```

BC

```
    AGU         CA
UGGC   CCCGGUUC  \
••||   ||||||||  U
GUCG   GGGCCAAG  /
    CCU         AC
------------------------
AAGACCAGA
-------------------------------------------------------
```

Helix III

```
333333-333444444444---4555---55555--5566-666666667777
123456-789012345678---9012---34567--8901-234567890123
334288-134383384448---3383---14136--3361-363226448888
```

BC

```
    CG-     A  A   GAUC     ACA     CU     G        CC
GGCU   AGCG GG CCC     GG-G   ACAGU  GGUA GUGUUUCC  \
||||   |||| || |||     || |   ||||•  ||•| |•|||•||  A
CCGA   UCGC CC GGG     CC-C   UGUCG  CCGU CGCAAGGG  /
    AUG     C  A   ----     GGC     --     A        CC
-------------------------------------------------------
GGCA
---------------------------
```

Helix IV

```
    U-        -   U-  GCCG
GGAA  UUUGGUU UCG  GC    U
|•||  ||||||• |||  ||    |
CUUU  AAACCAG AGC  CG    G
    CC        G   UC  AAAA
---------------------------
AACAUUC->
---------------------------
```

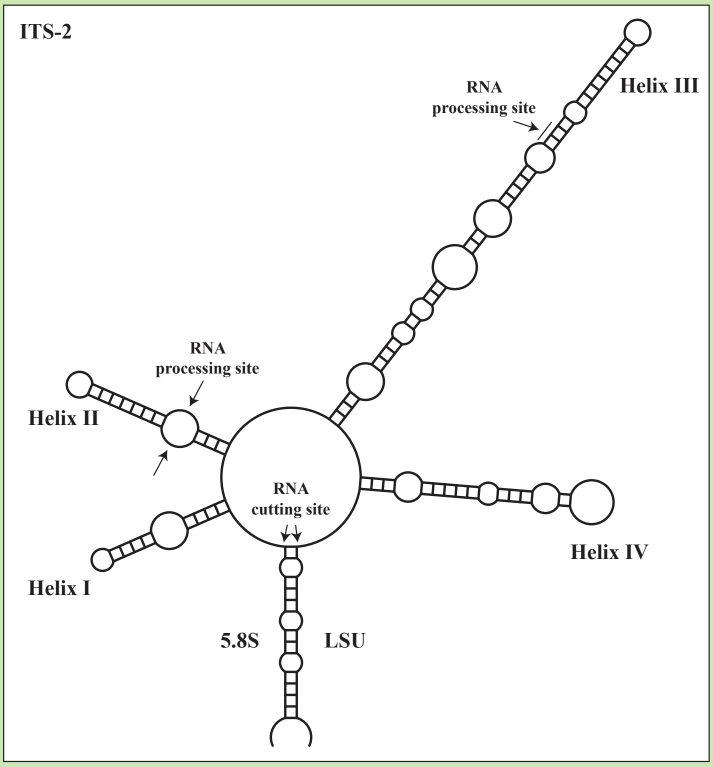

Barcode legend:

1 = A-U
2 = U-A
3 = G-C
4 = C-G
5 = G•U
6 = U•G
7 = mismatch
8 = deletion,single or unpaired bases

ITS-2 rRNA secondary structure model of *Coccomyxa galuniae* strain SAG 2253 (BC-5) HG972996

Barcode 5
ITS2-E

5.8S/LSU stem

```
---00000-000-0111-11
---12345-678-9012-34
---23442-154-2453-26
```

BC

```
GUC     C   G    C
   UGCCU AGC UCGG UU->
   ||||| |•| ||•| |•
   ACGGA UUG AGUC AG<-
AGA     C   -    C
------------------------
->CACCC
------------------------
```

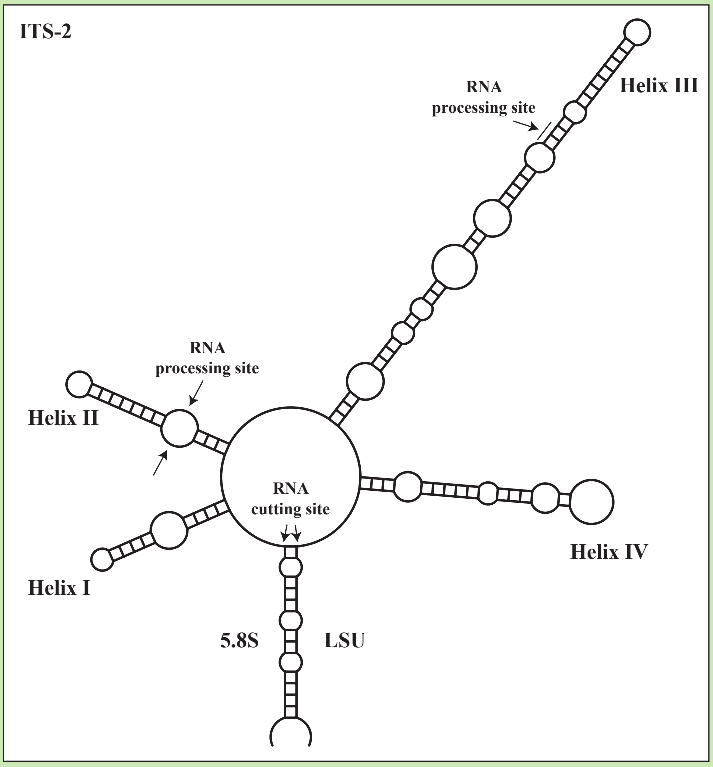

Helix I

```
11111
56789
64141
```

BC

```
     CA-     U
UCACA   CCCGC C
•||||   ||||| |
GGUGU   GGGCG G
     AGC     A
------------------------
AUC
------------------------
```

Helix II

```
22222222223
01234567890
65347774443
```

BC

```
    AGU        CA
UGGC   CCCGGUUC  \
••||   ||||||||  U
GUCG   GGGCCAAG  /
    CCU        AC
------------------------
AAGACCAGA
--------------------------------------------------------
```

Barcode legend:

1 = A-U
2 = U-A
3 = G-C
4 = C-G
5 = G•U
6 = U•G
7 = mismatch
8 = deletion,single or unpaired bases

Helix III

```
333333-333444444444---4555---55555--5566-666666667777
123456-789012345678---9012---34567--8901-234567890123
334288-134383384448---3383---14136--3361-363226448888
```

BC

```
    CG-    A  A   GAUC    ACA     CU    G        CC
GGCU   AGCG GG CCC    GG-G   ACAGU  GGUA GUGUUUCC  \
||||   |||| || |||    || |   ||||•  ||•| |•|||•||  A
CCGA   UCGC CC GGG    CC-C   UGUCG  CCGU CGCAAGGG  /
    AUG    C  A   ----    GGC     --    A        CC
--------------------------------------------------------
GGCA
---------------------------
```

Helix IV

```
    U-        -   U-  GCCG
GGAA  UUUGGUU UCG  GC    U
|•||  ||||||• |||  ||    |
CUUU  AAACCAG AGC  CG    G
    CC       G   UC  AAAA
---------------------------
AACAUUC->
---------------------------
```

ITS-2 rRNA secondary structure model of *Coccomyxa galuniae* strain SAG 2254 (BC-5) HG972997

Barcode 5
ITS2-E

5.8S/LSU stem

```
---00000-000-0111-11
---12345-678-9012-34
```

```
---23442-154-2453-26
```

BC

```
GUC     C   G    C
   UGCCU AGC UCGG UU->
   ||||| |•| ||•| |•
   ACGGA UUG AGUC AG<-
AGA     C   -    C
------------------------
->CACCC
------------------------
```

Helix I

```
11111
56789
```

```
64141
```

BC

```
     CA-     U
UCACA   CCCGC C
•||||   ||||| |
GGUGU   GGGCG G
     AGC     A
------------------------
AUC
------------------------
```

Helix II

```
22222222223
01234567890
```

```
65347774443
```

BC

```
    AGU        CA
UGGC   CCCGGUUC  \
••||   ||||||||  U
GUCG   GGGCCAAG  /
    CCU        AC
------------------------
AAGACCAGA
-------------------------------------------------------
```

Helix III

```
333333-333444444444---4555---55555--5566-666666667777
123456-789012345678---9012---34567--8901-234567890123
```

```
334288-134383384448---3383---14136--3361-363226448888
```

BC

```
    CG-    A  A   GAUC    ACA     CU    G        CC
GGCU   AGCG GG CCC    GG-G   ACAGU  GGUA GUGUUUCC  \
||||   |||| || |||    || |   ||||•  ||•| |•|||•||  A
CCGA   UCGC CC GGG    CC-C   UGUCG  CCGU CGCAAGGG  /
    AUG    C  A   ----    GGC     --    A        CC
-------------------------------------------------------
GGCA
---------------------------
```

Helix IV

```
    U-       -   U-  GCCG
GGAA  UUUGGUU UCG  GC    U
|•||  ||||||• |||  ||    |
CUUU  AAACCAG AGC  CG    G
    CC       G   UC  AAAA
---------------------------
AACAUUC->
---------------------------
```

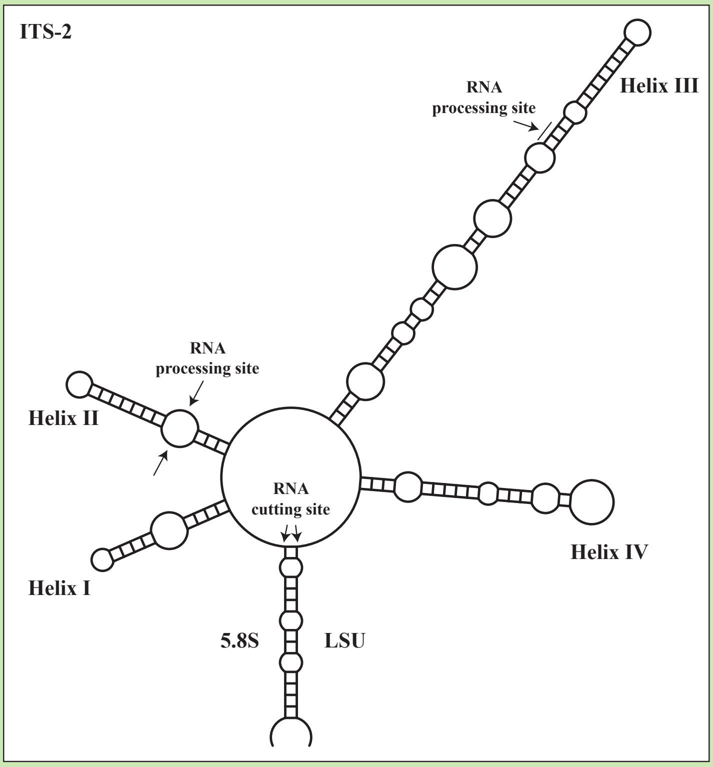

Barcode legend:

1 = A-U
2 = U-A
3 = G-C
4 = C-G
5 = G•U
6 = U•G
7 = mismatch
8 = deletion,single or unpaired bases

ITS-2 rRNA secondary structure model of *Coccomyxa dispar* strain SAG 49.84 (BC-6) HG972998

Barcode 6 ITS2-F

5.8S/LSU stem

```
---00000-000-0111-11
---12345-678-9012-34
---23442-154-2453-26
```

BC

```
GCC     C   G    U
   UGCCU AGC UCGG UU->
   ||||| |•| ||•| |•
   ACGGA UUG AGUC AG<-
AGA     C   -    C
------------------------
->ACACCC
------------------------
```

Helix I

```
11111
56789
64224
```

BC

```
      -     U
UCUUCU ACCAU U
•||||| ||||• |
GGAAGA UGGUG U
      G     U
------------------------
ACC
------------------------
```

Helix II

```
22222222223
01234567890
65347774443
```

BC

```
    UGU          CG
UGGC   CCCGAUUGUU  \
••||   ||||||||||  U
GUCG   GGGCUAACAA  /
    CCU          CG
------------------------
AAGAACAGA
-------------------------------------------------------
```

Helix III

```
333--333-3334-444-444-44----45-5555555--5566-666666667777
123--456-7890-123-456-78----90-1234567--8901-234567890123
334--484-1343-233-444-42----33-4114136--3361-343122888384
```

BC

```
   --   G    -   A   A  CCAC  G       CU    G            UA
GGC  C-C AGCG UGG CCC CU    GG CAACAGU  GGUA GCGAUU---G-C  C
|||  | | |||| ||| ||| ||    || ||||||•  ||•| ||||||   | |  |
CCG  G-G UCGC ACC GGG GA    CC GUUGUCG  CCGU CGCUAA---C-G  A
   AA   -    U   A   -  ----  G       --    A            UA
-------------------------------------------------------
GCAAGCA
---------------------------
```

Helix IV

```
  AAA
GG   C
||   |
CC   C
  AAA
---------------------------
AAACAUUC->
---------------------------
```

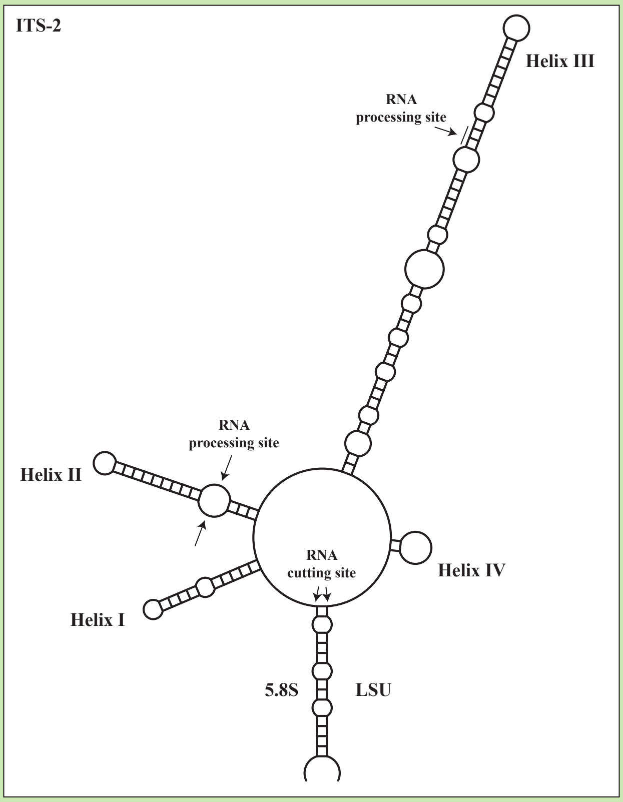


```
Barcode legend:

     1 = A-U
     2 = U-A
     3 = G-C
     4 = C-G
     5 = G•U
     6 = U•G
   7 = mismatch
8 = deletion,single or unpaired bases
```

ITS-2 rRNA secondary structure model of *Coccomyxa viridis* strain SAG 216-1 (BC-7a) HG972999

Barcode 7a ITS2-G1

5.8S/LSU stem

```
---00000-000-0111-11
---12345-678-9012-34
---23442-156-2453-26
```

BC

```
GCC     C   G    U
   UGCCU AGU UCGG UU->
   ||||| |•• ||•| |•
   ACGGA UUG AGUC AG<-
GGA     C   -    C
------------------------
->CACCC
------------------------
```

Helix I

```
1111---1
5678---9
6434---4
```

BC

```
    CCA        A--     U
UCGC   CCCUUUCU   UUCUC C
•|||   ||||•|||   ||||| |
GGCG   GGGAGAGA   AAGAG A
    AG-        CAC     C
------------------------
AUC
------------------------
```

Helix II

```
22222222223
01234567890
65347774443
```

BC

```
    CGU       U
UGGC   CCCGGUC C
••||   ||||||| |
GUCG   GGGCCAG A
    UCU       C
------------------------
AAGCGCAGA
-------------------------------------------------------
```

Helix III

```
333-333-3334444-4444-------4-455--55-555--55566-6666--66667777
123-456-7890123-4567-------8-901--23-456--78901-2345--67890123
334-286-1343833-2444-------3-333--41-413--43361-3631--44822424
```

BC

```
   -   G       A    UAUAGAU U   AA  A   AC     G    GC        U
GGC U-U AGCG-GG UCCC       G GGG  CA CAG  CGGUA GUGA  CC-UUCUC C
||| | • |||| || ||||       | |||  || |||  |||•| |•||  || ||||| |
CCG A-G UCGC-CC AGGG       C CCC  GU GUC  GCCGU CGCU  GG-AAGAG C
   C   G       -    ------- -   AG  C   --     A    A-        G
-------------------------------------------------------
CAGCAG
---------------------------
```

Helix IV

```
   A---      GA       U
GAG    CGGUGU  CCGGUCC C
|||    |||||•  ||||||| |
CUC    GCCACG  GGCCAGG G
   AUAC      AA       C
---------------------------
AACCAUUC->
---------------------------
```

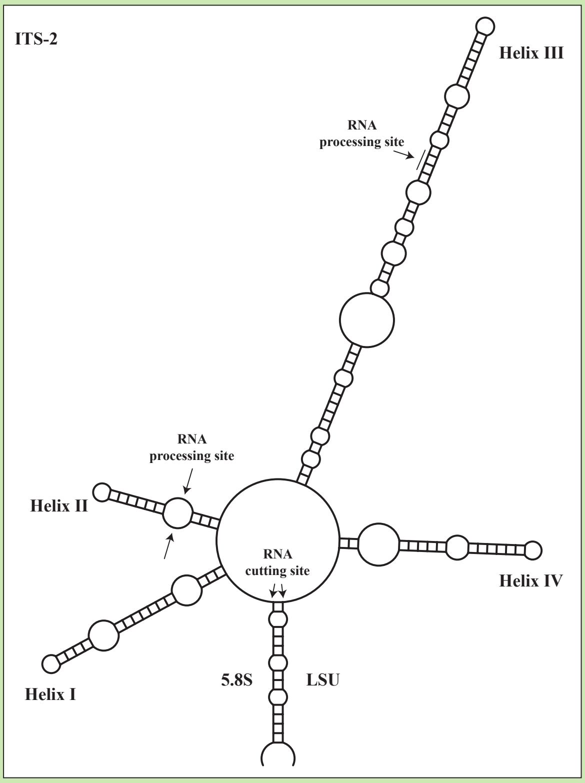


```
Barcode legend:

1 = A-U
2 = U-A
3 = G-C
4 = C-G
5 = G•U
6 = U•G
7 = mismatch
8 = deletion,single or unpaired bases
```

ITS-2 rRNA secondary structure model of *Coccomyxa viridis* strain Wien C19 (BC-7a) HG973000

Barcode 7a ITS2-G2

5.8S/LSU stem

```
---00000-000-0111-11
---12345-678-9012-34
---23442-156-2453-26
```

BC

```
GCC     C   G    U
   UGCCU AGU UCGG UU->
   ||||| |•• ||•| |•
   ACGGA UUG AGUC AG<-
GGA     C   -    C
------------------------
->CACCC
------------------------
```

Helix I

```
1111---1
5678---9
6434---4
```

BC

```
    CCA        A--     U
UCGC   CCCUUUCU   UUCUU C
•|||   ||||•|||   ||||• |
GGCG   GGGAGAGA   AAGAG A
    AG-        CAC     C
------------------------
AUC
------------------------
```

Helix II

```
22222222223
01234567890
65347774443
```

BC

```
    CGU       U
UGGC   CCCGGUC C
••||   ||||||| |
GUCG   GGGCCAG A
    UCU       C
------------------------
AAGCGCAGA
-------------------------------------------------------
```

Helix III

```
333-333-3334444-4444-------4-455--55-555--55566-6666--66667777
123-456-7890123-4567-------8-901--23-456--78901-2345--67890123
334-286-1343833-2444-------3-333--41-413--43361-3631--44822424
```

BC

```
   -   G       A    UAUAGAU U   AA  A   AC      G    GC        U
GGC U-U AGCG-GG UCCC       G GGG  CA CAG  CGGUA GUGA  CC-UUCUC C
||| | • |||| || ||||       | |||  || |||  |||•| |•||  || ||||| |
CCG A-G UCGC-CC AGGG       C CCC  GU GUC  GCCGU CGCU  GG-AAGAG C
   C   G       -    ------- -   AG  C   --      A    A-        G
-------------------------------------------------------
CAGCAG
---------------------------
```

Helix IV

```
   A---      GA       U
GAG    CGGUGU  CCGGUCC C
|||    |||||•  ||||||| |
CUC    GCCACG  GGCCAGG G
   AUAC      AA       C
---------------------------
AACCAUUC->
---------------------------
```

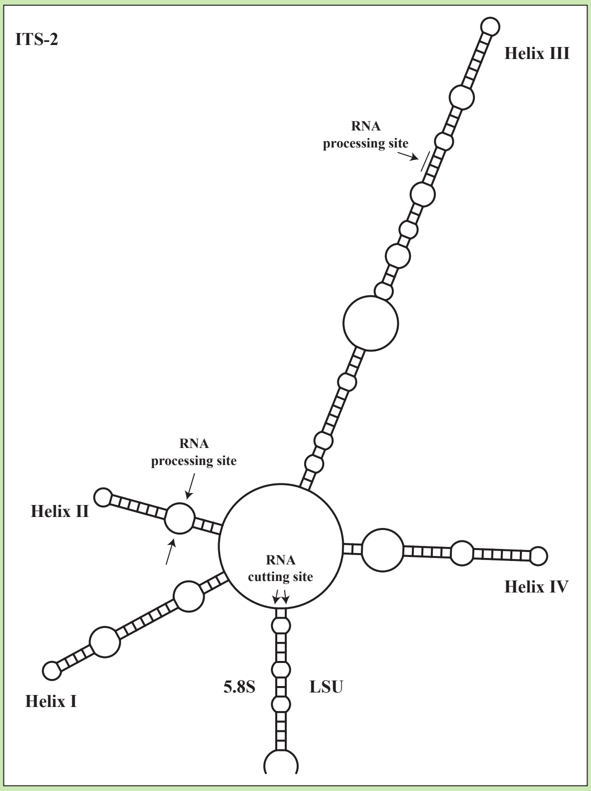


```
Barcode legend:

1 = A-U
2 = U-A
3 = G-C
4 = C-G
5 = G•U
6 = U•G
7 = mismatch
8 = deletion,single or unpaired bases
```

ITS-2 rRNA secondary structure model of *Coccomyxa viridis* strain SAG 216-4 (BC-7a) HG973001

Barcode 7a ITS2-G3

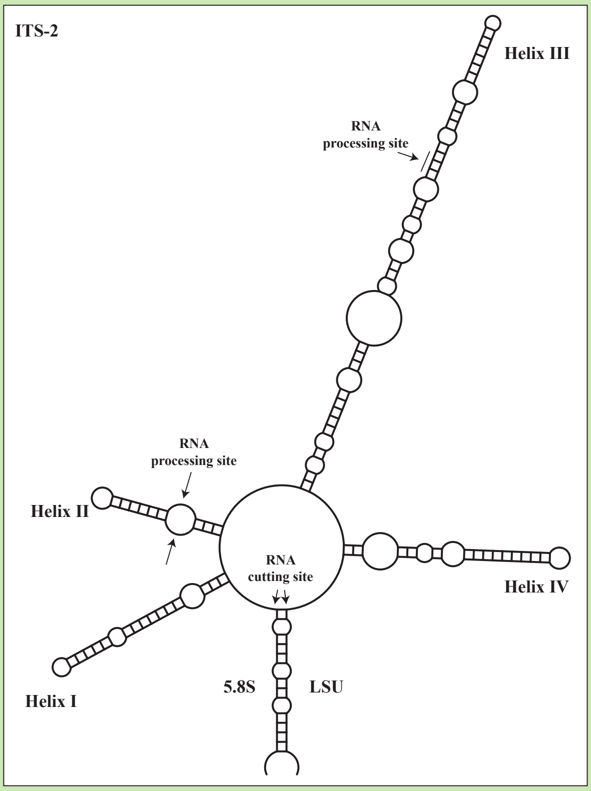


```
Barcode legend:

1 = A-U
2 = U-A
3 = G-C
4 = C-G
5 = G•U
6 = U•G
7 = mismatch
8 = deletion,single or unpaired bases
```

5.8S/LSU stem

```
---00000-000-0111-11
---12345-678-9012-34
---23442-156-2453-26
```

BC

```
GCC     C   G    U
   UGCCU AGU UCGG UU->
   ||||| |•• ||•| |•
   ACGGA UUG AGUC AG<-
GGA     C   -    C
------------------------
->CACCC
------------------------
```

Helix I

```
1111--1
5678--9
6434--4
```

BC

```
    CA        -      U
UCGC  CCCCUUUC UCUGUC C
•|||  |||||||| |||||| |
GGCG  GGGGAAAG AGACAG C
    C-        C      G
------------------------
AUC
------------------------
```

Helix II

```
22222222223
01234567890
65347774443
```

BC

```
    CGU       UU
UGGC   CCCGGUC  \
••||   ||||•||  U
GUCG   GGGCUAG  /
    UCU       UC
------------------------
AAGCGCAGA
-------------------------------------------------------
```

Helix III

```
333-333-3334444--4444-------4-455--55-555--55566-6666--66667777
123-456-7890123--4567-------8-901--23-456--78901-2345--67890123
334-286-1343833--2444-------3-333--41-413--43361-3631--44822424
```

BC

```
   -   G       AU    UUCUAAU U   AA  A   AC      G    GC        C
GGC U-U AGCG-GG  UCCC       G GGG  CA CAG  CGGUA GUGA  CC-UUCUC \
||| | • |||| ||  ||||       | |||  || |||  |||•| |•||  || ||||| U
CCG A-G UCGC-CC  AGGG       C CCC  GU GUC  GCCGU CGCU  GG-AAGAG /
   C   G       --    ------- -   AG  C   --     A    A-        C
-------------------------------------------------------
CAGCAG
---------------------------
```

Helix IV

```
   A---   U  GA          CA
GAG    CGG GU  CGGUGGUUCC  \
|||    ||| ||  ||•|||||||  C
CUC    GCC CA  GCUACCAAGG  /
   ACUA   C  AA          AG
---------------------------
AACCAUUC->
---------------------------
```

ITS-2 rRNA secondary structure model of *Coccomyxa viridis* strain SAG 216-14 (BC-7a) HG973002

Barcode 7a
ITS2-G3

5.8S/LSU stem

```
---00000-000-0111-11
---12345-678-9012-34
---23442-156-2453-26
```

BC

```
GCC     C   G    U
   UGCCU AGU UCGG UU->
   ||||| |•• ||•| |•
   ACGGA UUG AGUC AG<-
GGA     C   -    C
------------------------
->CACCC
------------------------
```

Helix I

```
1111--1
5678--9
6434--4
```

BC

```
    CA        -      U
UCGC  CCCCUUUC UCUGUC C
•|||  |||||||| |||||| |
GGCG  GGGGAAAG AGACAG C
    C-        C      G
------------------------
AUC
------------------------
```

Helix II

```
22222222223
01234567890
65347774443
```

BC

```
    CGU       UU
UGGC   CCCGGUC  \
••||   ||||•||  U
GUCG   GGGCUAG  /
    UCU       UC
------------------------
AAGCGCAGA
-------------------------------------------------------
```

Helix III

```
333-333-3334444--4444-------4-455--55-555--55566-6666--66667777
123-456-7890123--4567-------8-901--23-456--78901-2345--67890123
334-286-1343833--2444-------3-333--41-413--43361-3631--44822424
```

BC

```
   -   G       AU    UUCUAAU U   AA  A   AC     G    GC        C
GGC U-U AGCG-GG  UCCC       G GGG  CA CAG  CGGUA GUGA  CC-UUCUC \
||| | • |||| ||  ||||       | |||  || |||  |||•| |•||  || ||||| U
CCG A-G UCGC-CC  AGGG       C CCC  GU GUC  GCCGU CGCU  GG-AAGAG /
   C   G        --   ------- -   AG  C   --     A    A-        C
-------------------------------------------------------
CAGCAG
---------------------------
```

Helix IV

```
   A---   U  GA          CA
GAG    CGG GU  CGGUGGUUCC  \
|||    ||| ||  ||•|||||||  C
CUC    GCC CA  GCUACCAAGG  /
   ACUA   C  AA          AG
---------------------------
AACCAUUC->
---------------------------
```

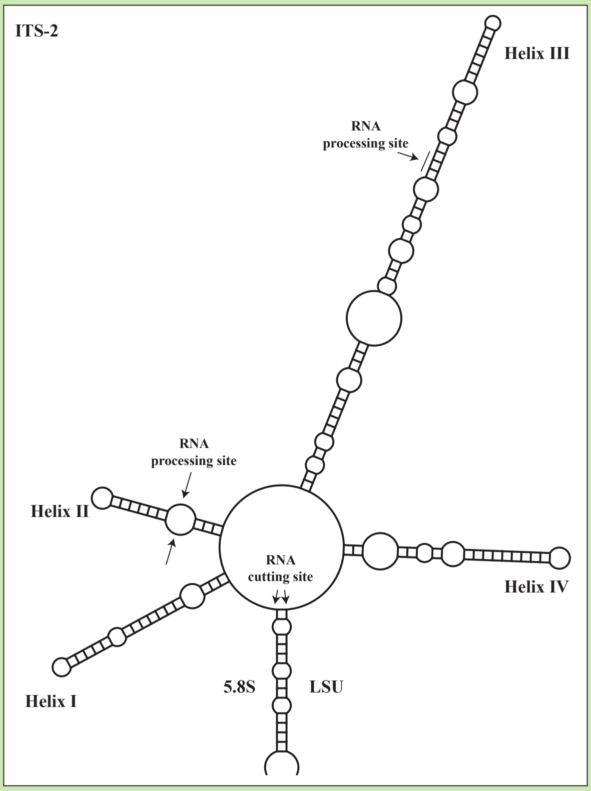

Barcode legend:

1 = A-U
2 = U-A
3 = G-C
4 = C-G
5 = G•U
6 = U•G
7 = mismatch
8 = deletion,single or unpaired bases

ITS-2 rRNA secondary structure model of *Coccomyxa viridis* strain SAG 2104 (BC-7a) HG973003

Barcode 7a ITS2-G3

5.8S/LSU stem

```
---00000-000-0111-11
---12345-678-9012-34
---23442-156-2453-26
```

BC

```
GCC     C   G    U
   UGCCU AGU UCGG UU->
   ||||| |•• ||•| |•
   ACGGA UUG AGUC AG<-
GGA     C   -    C
------------------------
->CACCC
------------------------
```

Helix I

```
1111--1
5678--9
6434--4
```

BC

```
    CA        -      U
UCGC  CCCCUUUC UCUGUC C
•|||  |||||||| |||||| |
GGCG  GGGGAAAG AGACAG C
    C-        C      G
------------------------
AUC
------------------------
```

Helix II

```
22222222223
01234567890
65347774443
```

BC

```
    CGU       UU
UGGC   CCCGGUC  \
••||   ||||•||  U
GUCG   GGGCUAG  /
    UCU       UC
------------------------
AAGCGCAGA
--------------------------------------------------------
```

Helix III

```
333-333-3334444--4444-------4-455--55-555--55566-6666--66667777
123-456-7890123--4567-------8-901--23-456--78901-2345--67890123
334-286-1343833--2444-------3-333--41-413--43361-3631--44822424
```

BC

```
   -   G       AU    UUCUAAU U    AA A    AC     G     GC        C
GGC U-U AGCG-GG  UCCC       G GGG  CA CAG  CGGUA GUGA  CC-UUCUC \
||| | • |||| ||  ||||       | |||  || |||  |||•| |•||  || ||||| U
CCG A-G UCGC-CC  AGGG       C CCC  GU GUC  GCCGU CGCU  GG-AAGAG /
   C   G       --    ------- -    AG C    --     A     A-        C
--------------------------------------------------------
CAGCAG
---------------------------
```

Helix IV

```
   A---   U  GA          CA
GAG    CGG GU  CGGUGGUUCC  \
|||    ||| ||  ||•|||||||  C
CUC    GCC CA  GCUACCAAGG  /
   ACUA   C  AA          AG
---------------------------
AACCAUUC->
---------------------------
```

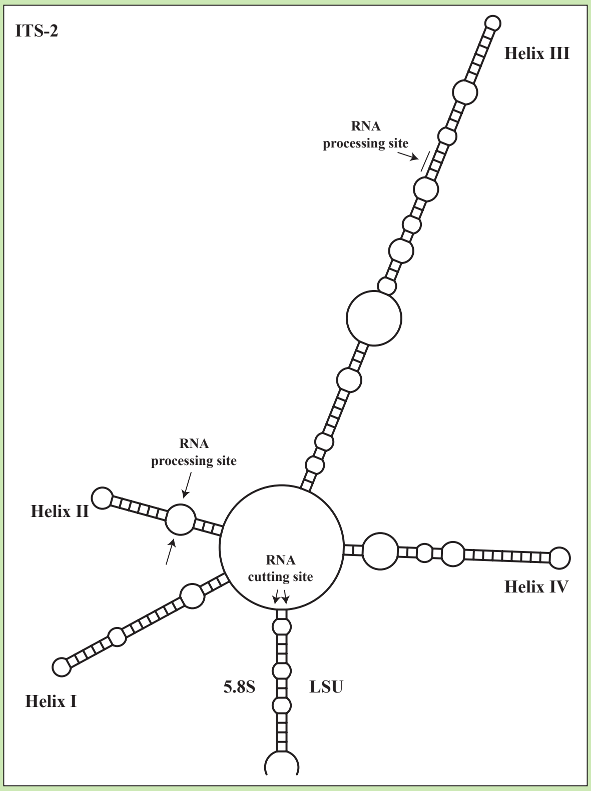


```
Barcode legend:

1 = A-U
2 = U-A
3 = G-C
4 = C-G
5 = G•U
6 = U•G
7 = mismatch
8 = deletion,single or unpaired bases
```

ITS-2 rRNA secondary structure model of *Coccomyxa viridis* strain SAG 2040 (BC-7b) HG973004

Barcode 7b
ITS2-G4

5.8S/LSU stem

```
---00000-000-0111-11
---12345-678-9012-34
---23442-156-2453-26
```

BC

```
GCC     C   G    U
   UGCCU AGU UCGG UU->
   ||||| |•• ||•| |•
   ACGGA UUG AGUC AG<-
GGA     C   -    C
------------------------
->CACCC
------------------------
```

Helix I

```
1111-1
5678-9
6434-4
```

BC

```
    C U       -    U  C
UCGC C CCCUUUC UUUU UC U
•||| | ||||||| |||• || |
GGCG G GGGAAAG AAAG AG C
    U -       U    C  G
------------------------
AUC
------------------------
```

Helix II

```
22222222223
01234567890
65347774443
```

BC

```
    CGU       UC
UGGC   CCCGGUC  \
••||   |||||||  C
GUCG   GGGCCAG  /
    UCU       UU
------------------------
AAGCGCAGA
-------------------------------------------------------
```

Helix III

```
333-3333334444--4444-------4-455--55-555--55566-6666--66667777
123-4567890123--4567-------8-901--23-456--78901-2345--67890123
334-2661343833--2444-------3-333--41-413--43361-3631--44822424
```

BC

```
   -          AU    UUCUAAU U   AA  A   AC     G    GC        CU
GGC UUUAGCG-GG  UCCC       G GGG  CA CAG  CGGUA GUGA  CC-UUCUC  \
||| |••|||| ||  ||||       | |||  || |||  |||•| |•||  || |||||  C
CCG AGGUCGC-CC  AGGG       C CCC  GU GUC  GCCGU CGCU  GG-AAGAG  /
   C          --    ------- -   AG  C   --     A    A-        AG
-------------------------------------------------------
CAGCAG
---------------------------
```

Helix IV

```
   A---     GUAA        U
GAG    CGGUG   UGAUGGGUC C
|||    |||||   |•||||||| |
CUC    GCCAC   AUUACCCAG A
   ACUA     AACA        C
---------------------------
AACCAUUC->
---------------------------
```

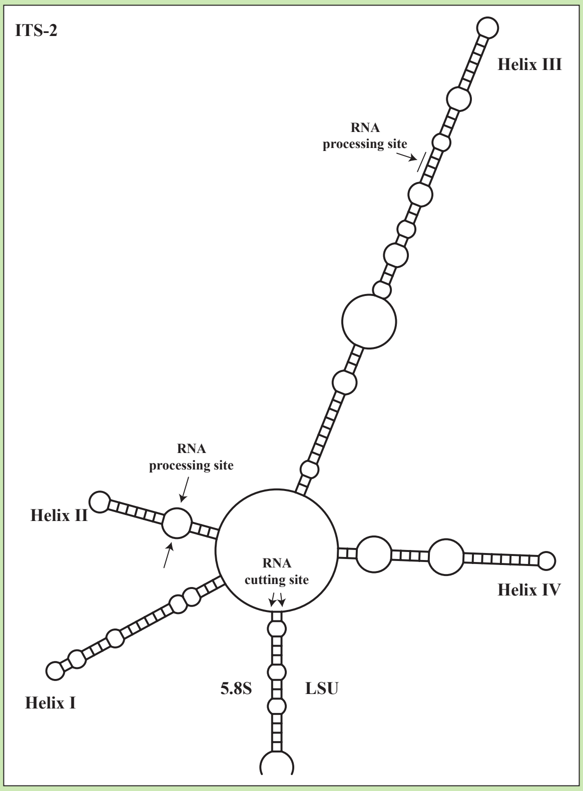


```
Barcode legend:

1 = A-U
2 = U-A
3 = G-C
4 = C-G
5 = G•U
6 = U•G
7 = mismatch
8 = deletion,single or unpaired bases
```

ITS-2 rRNA secondary structure model of *Coccomyxa viridis* strain SAG 2127 (BC-7b) HG973005

Barcode 7b
ITS2-G4

5.8S/LSU stem

```
---00000-000-0111-11
---12345-678-9012-34
---23442-156-2453-26
```

BC

```
GCC      C   G    U
   UGCCU AGU UCGG UU->
   ||||| |•• ||•| |•
   ACGGA UUG AGUC AG<-
GGA      C   -    C
------------------------
->CACCC
------------------------
```

Helix I

```
1111-1
5678-9
6434-4
```

BC

```
    C U       -    U  C
UCGC C CCCUUUC UUUU UC U
•||| | ||||||| |||• || |
GGCG G GGGAAAG AAAG AG C
    U -       U    C  G
------------------------
AUC
------------------------
```

Helix II

```
22222222223
01234567890
65347774443
```

BC

```
    CGU       UC
UGGC   CCCGGUC  \
••||   |||||||  C
GUCG   GGGCCAG  /
    UCU       UU
------------------------
AAGCGCAGA
-------------------------------------------------------
```

Helix III

```
333-3333334444--4444-------4-455--55-555--55566-6666--66667777
123-4567890123--4567-------8-901--23-456--78901-2345--67890123
334-2661343833--2444-------3-333--41-413--43361-3631--44822424
```

BC

```
   -          AU    UUCUAAU U   AA  A   AC     G    GC        CU
GGC UUUAGCG-GG  UCCC       G GGG  CA CAG  CGGUA GUGA  CC-UUCUC  \
||| |••|||| ||  ||||       | |||  || |||  |||•| |•||  || |||||  C
CCG AGGUCGC-CC  AGGG       C CCC  GU GUC  GCCGU CGCU  GG-AAGAG  /
   C          --    ------- -   AG  C   --     A    A-        AG
-------------------------------------------------------
CAGCAG
---------------------------
```

Helix IV

```
   A---     GUAA         U
GAG    CGGUG    UGAUGGGUC C
|||    |||||    |•||||||| |
CUC    GCCAC    AUUACCCAG A
   ACUA     AACA         C
---------------------------
AACCAUUC->
---------------------------
```

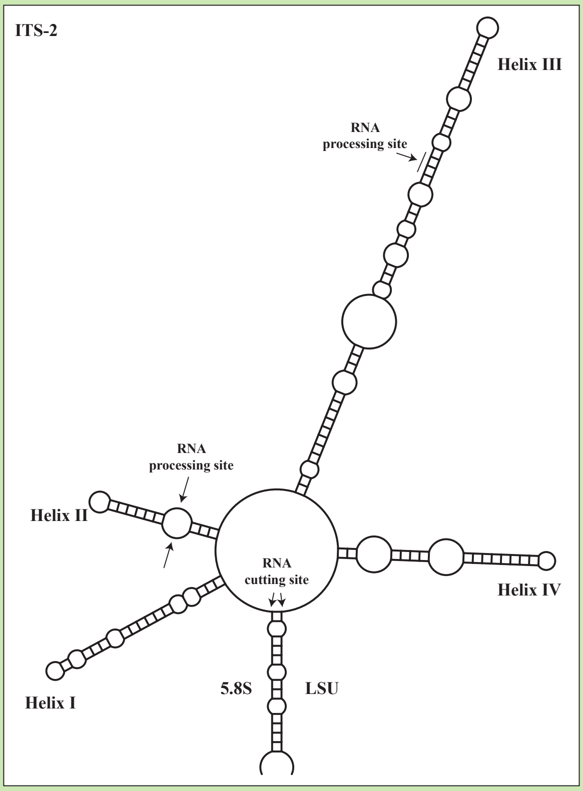


```
Barcode legend:

1 = A-U
2 = U-A
3 = G-C
4 = C-G
5 = G•U
6 = U•G
7 = mismatch
8 = deletion,single or unpaired bases
```

ITS-2 rRNA secondary structure model of *Coccomyxa viridis* strain SAG 2325 (BC-7b) HG973006

Barcode 7b ITS2-G4

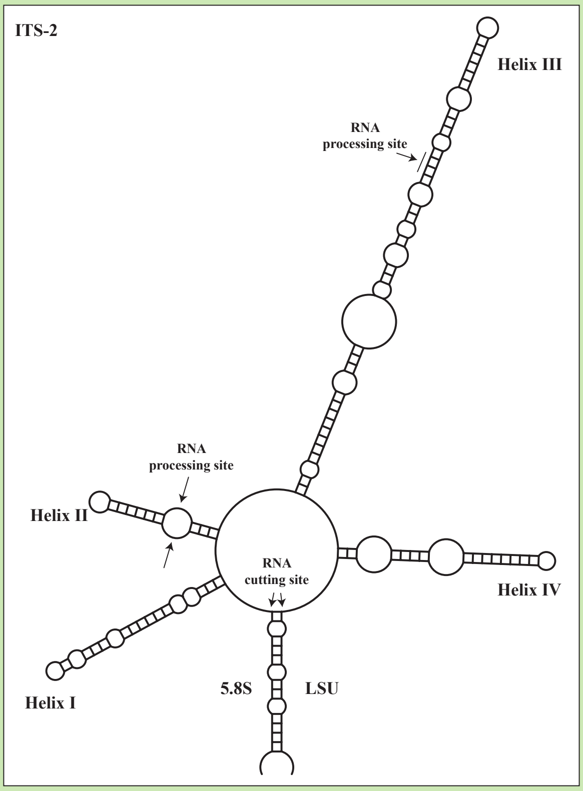

5.8S/LSU stem

```
---00000-000-0111-11
---12345-678-9012-34
---23442-156-2453-26
```

BC

```
GCC     C   G    U
   UGCCU AGU UCGG UU->
   ||||| |•• ||•| |•
   ACGGA UUG AGUC AG<-
GGA     C   -    C
------------------------
->CACCC
------------------------
```

Helix I

```
1111-1
5678-9
6434-4
```

BC

```
    C U       -    U  C
UCGC C CCCUUUC UUUU UC U
•||| | ||||||| |||• || |
GGCG G GGGAAAG AAAG AG C
    U -       U    C  G
------------------------
AUC
------------------------
```

Helix II

```
22222222223
01234567890
65347774443
```

BC

```
    CGU       UC
UGGC    CCCGGUC  \
••||    |||||||  C
GUCG    GGGCCAG  /
    UCU       UU
------------------------
AAGCGCAGA
-------------------------------------------------------
```

Helix III

```
333-3333334444--4444-------4-455--55-555--55566-6666--66667777
123-4567890123--4567-------8-901--23-456--78901-2345--67890123
334-2661343833--2444-------3-333--41-413--43361-3631--44822424
```

BC

```
   -          AU    UUCUAAU U   AA  A   AC     G    GC        CU
GGC UUUAGCG-GG  UCCC       G GGG  CA CAG  CGGUA GUGA  CC-UUCUC  \
||| |••|||| ||  ||||       | |||  || |||  |||•| |•||  || |||||  C
CCG AGGUCGC-CC  AGGG       C CCC  GU GUC  GCCGU CGCU  GG-AAGAG  /
   C          --    ------- -   AG  C   --     A    A-        AG
-------------------------------------------------------
CAGCAG
---------------------------
```

Helix IV

```
   A---     GUAA        U
GAG    CGGUG    UGAUGGGUC C
|||    |||||    |•||||||| |
CUC    GCCAC    AUUACCCAG A
   ACUA     AACA        C
---------------------------
AACCAUUC->
---------------------------
```

Barcode legend:

1 = A-U
2 = U-A
3 = G-C
4 = C-G
5 = G•U
6 = U•G
7 = mismatch
8 = deletion,single or unpaired bases

ITS-2 rRNA secondary structure model of *Coccomyxa viridis* strain CAUP H5103 (BC-7b) HG973007

Barcode 7b
ITS2-G5

5.8S/LSU stem

```
---00000-000-0111-11
---12345-678-9012-34
```

```
---23442-156-2453-26
```

BC

```
GCC     C   G    U
   UGCCU AGU UCGG UU->
   ||||| |•• ||•| |•
   ACGGA UUG AGUC AG<-
GGA     C   -    C
------------------------
->CACCC
------------------------
```

Helix I

```
1111-1
5678-9
```

```
6434-4
```

BC

```
    C U        -    U  C
UCGC C CCCUUUC UUUC UC U
•||| | ||||||| |||| || |
GGCG G GGGAAAG AAAG AG C
    U -        U    C  G
------------------------
AUC
------------------------
```

Helix II

```
22222222223
01234567890
```

```
65347774443
```

BC

```
    CGU       UC
UGGC    CCCGGUC  \
••||    |||||||  C
GUCG    GGGCCAG  /
    UCU       UU
------------------------
AAGCGCAGA
-------------------------------------------------------
```

Helix III

```
333-3333334444--4444-------4-455--55-555--55566-6666--66667777
123-4567890123--4567-------8-901--23-456--78901-2345--67890123
```

```
334-2661343833--2444-------3-333--41-413--43361-3631--44822424
```

BC

```
   -          AU    UUCUAAU U   AA  A   AC     G    GC        CU
GGC UUUAGCG-GG  UCCC       G GGG  CA CAG  CGGUA GUGA  CC-UUCUC  \
||| |••|||| ||  ||||       | |||  || |||  |||•| |•||  || |||||  C
CCG AGGUCGC-CC  AGGG       C CCC  GU GUC  GCCGU CGCU  GG-AAGAG  /
   C          --    ------- -   AG  C   --     A    A-        AG
-------------------------------------------------------
CAGCAG
---------------------------
```

Helix IV

```
   A---     GUAA         U
GAG     CGGUG    UGAUGGGUC C
|||     |||||    |•||||||| |
CUC     GCCAC    AUUACCCAG A
   ACUA     AACA         C
---------------------------
AACCAUUC->
---------------------------
```

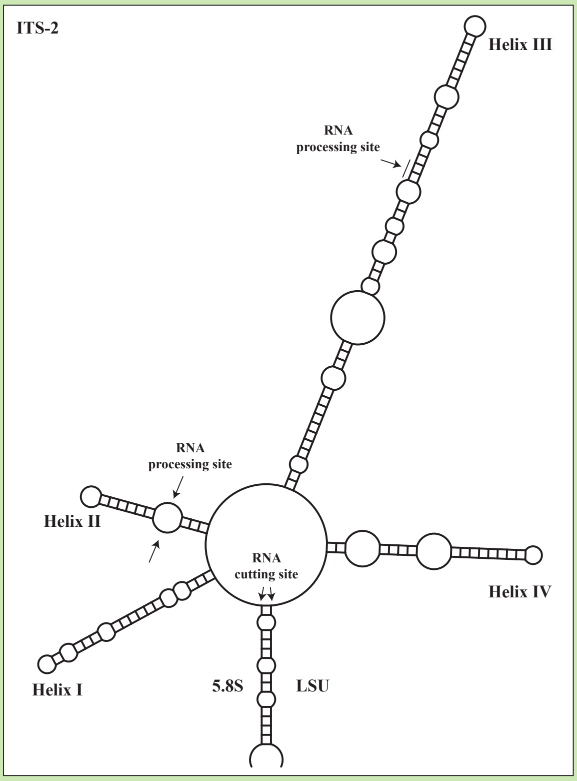

Barcode legend:

1 = A-U
2 = U-A
3 = G-C
4 = C-G
5 = G•U
6 = U•G
7 = mismatch
8 = deletion,single or unpaired bases

ITS-2 rRNA secondary structure model of *Coccomyxa subellipsoidea* uncultured (BC-1d) AY293946 *

Barcode 1d
ITS2-A4

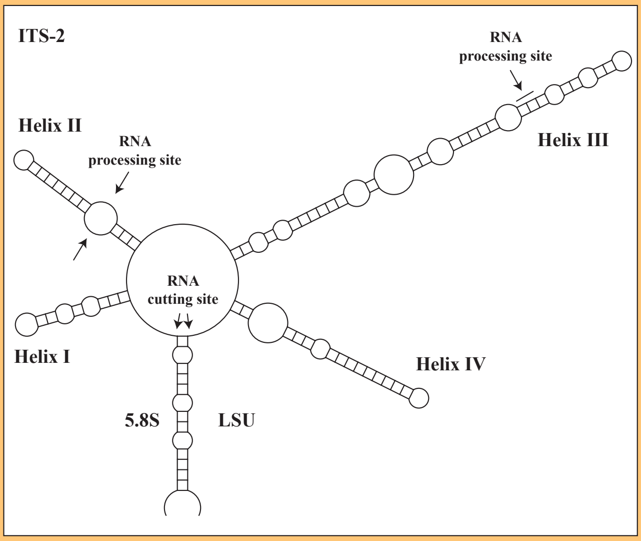

Barcode legend:

1 = A-U
2 = U-A
3 = G-C
4 = C-G
5 = G•U
6 = U•G
7 = mismatch
8 = deletion,single or unpaired bases

5.8S/LSU stem

```
---00000-000-0111-11
---12345-678-9012-34
---23442-154-2453-26
```

BC

```
GUC      C    G     C
   UGCCU AGC UCGG UU->
   ||||| |•| ||•| |•
   acgga uug aguC AG<-
aga      c    -     C
------------------------
->UACCC
------------------------
```

Helix I

```
1111-1
5678-9
6414-4
```

BC

```
    -  C   A
UCAC CC CCC A
•||| || ||| |
GGUG GG GGG U
    C  A   C
------------------------
ACC
------------------------
```

Helix II

```
22222222223
01234567890
65347774443
```

BC

```
    AGU        G
UGGC   CCCGGUCG C
••||   |||||||| |
GUCG   GGGCCAGC A
    CCU        A
------------------------
AAGACCAGA
-------------------------------------------------------
```

Helix III

```
333-333-333444444--444---455--555555--5566-666666667777
123-456-789012345--678---901--234567--8901-234567890123
334-284-134181383--448---533--114136--3361-363884488884
```

BC

```
   -   G         AC   GCAU   AA      CU    G   A        G
GGC U-C AGCA-AG-G   CC     GGG   AACAGU   GGUA GUG -CC----C C
||| | | |||| || |   ||     •||   |||||•   ||•| |•|  ||    | |
CCG A-G UCGU-UC-C   GG     UCC   UUGUCG   CCGU CGC -GG----G A
   A   G         GA   ----   GC      --    A   -        A
-------------------------------------------------------
GGC
---------------------------
```

Helix IV

```
   AA--    -          U
AGG     UCGU CGGUCGCCCU A
|||     |||• |||•|||||• |
UCC     AGCG GCCGGCGGGG G
   CACA    U          C
---------------------------
UUUC->
---------------------------
```

ITS-2 rRNA secondary structure model of *Coccomyxa simplex* strain GSE4G (BC-3c) HE586518 *

Barcode 3c ITS2-C8

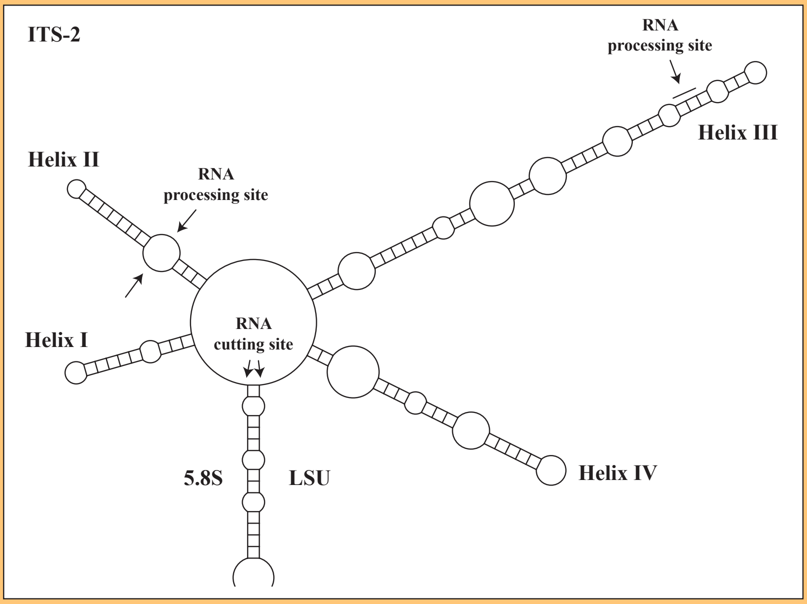

5.8S/LSU stem

```
---00000-000-0111-11
---12345-678-9012-34
---23442-154-2453-26
```

BC

```
GUC      C   G    C
   UGCCU AGC UCGG UU->
   |||||  |•|  ||•|  |•
   acgga uug aguc ag<-
aga      c   -    c
------------------------
->CACCC
------------------------
```

Helix I

```
1111-1
5678-9
6414-4
```

BC

```
    C      C
UCAC CCUCCU A
•|||  |||||• |
GGUG GGAGGG C
    C      C
------------------------
ACC
------------------------
```

Helix II

```
22222222223
01234567890
63347774443
```

BC

```
    CGU        U
UGGC   CCCGGUCG \
•|||   |||||||| U
GCCG   GGGCCAGC /
    CCU        G
------------------------
AAGAACAGA
-------------------------------------------------------
```

Helix III

```
333333-333444444444---4555---55555--5566-666666667777
123456-789012345678---9012---34567--8901-234567890123
334288-114313384448---3383---14136--3361-363184488884
```

BC

```
    CG-      A   AAUC    ACA     CU    G    -      G
GGCU   AACGAGG CCC    GG-G   ACAGU  GGUA GUGA CC----C C
||||   ||||||| |||    || |   ||||•  ||•| |•|| ||     | |
CCGA   UUGCUCC GGG    CC-C   UGUCG  CCGU CGCU GG----G G
    AUG      A   ----    GGC     --    A    A       A
-------------------------------------------------------
GGC
---------------------------
```

Helix IV

```
   AA---    -    CGA     CU
AGG     UCGC CGGU   CCUUUC  G
|||     |||| ||||   ||||||  |
UCC     AGCG GCCA   GGAAAG  A
   CACAC    C    AC-     CC
---------------------------
Uuuc->
---------------------------
```

```
Barcode legend:

1 = A-U
2 = U-A
3 = G-C
4 = C-G
5 = G•U
6 = U•G
7 = mismatch
8 = deletion,single or unpaired bases
```

ITS-2 rRNA secondary structure model of *Coccomyxa viridis* strain CR-2 (BC-7a) HE586519

Barcode 7a ITS2-G6

5.8S/LSU stem

```
---00000-000-0111-11
---12345-678-9012-34
---23442-156-2453-26
```

BC

```
GCC      C   G    U
   UGCCU AGU UCGG UU->
   ||||| |•• ||•| |•
   ACGGA UUG AGUC AG<-
GGA      C   -    C
------------------------
->CACCC
------------------------
```

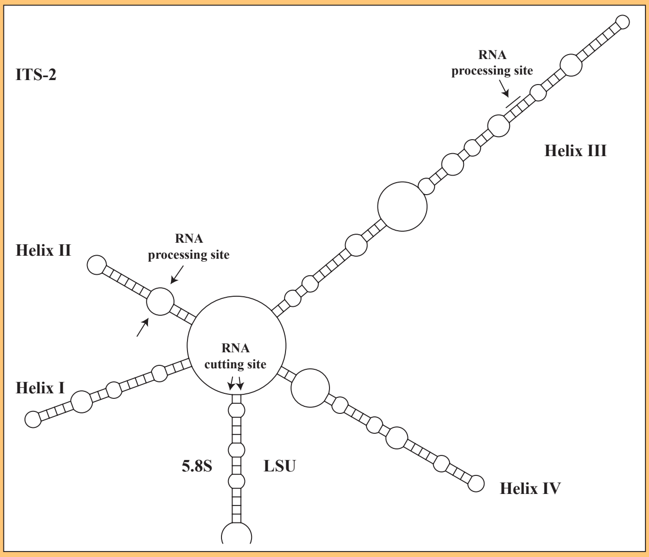

Helix I

```
1111-1
5678-9
6434-4
```

BC

```
    C     U   UU     U
UCGC CUCCC UYC  UUAUC U
•||| |•||| |||  ||||| |
GGCG GGGGG AAG  AAUAG C
    U     C   CU     G
------------------------
AUC
------------------------
```

```
Y = C or U
(should be a U)
```

Barcode legend:

```
1 = A-U
2 = U-A
3 = G-C
4 = C-G
5 = G•U
6 = U•G
7 = mismatch
8 = deletion,single or unpaired bases
```

Helix II

```
22222222223
01234567890
65347774443
```

BC

```
    CGU       CA
UGGC   CCCGAUU  \
••||   |||||||  U
GUCG   GGGCUAA  /
    UCU       CC
------------------------
AAGCGCAGA
-------------------------------------------------------
```

Helix III

```
333-333-3334444--4444-------4-455--55-555--55566-6666--66667777
123-456-7890123--4567-------8-901--23-456--78901-2345--67890123
334-286-1343833--2444-------3-333--41-413--43361-3631--44822424
```

BC

```
   -   G       AU    UUCUGAU C   AA  A   AC     G    GC        C
GGC U-U AGCG-GG  UCCC       G GGG  CA CAG  CGGUA GUGA  CC-UUCUC \
||| | • |||| ||  ||||       | |||  || ||   |||•| |•||  || ||||| U
CCG A-G UCGC-CC  AGGG       C CCC  GU GUC  GCCGU CGCU  GG-AAGAG /
   C   G       --    ------- -   AG  C   --     A    A-        C
-------------------------------------------------------
CAGCA
---------------------------
```

Helix IV

```
   -----  -    -  GA     A    U
GGA      GA CGGU GU  CUGGG GGUC C
|||      || ||•| ||  ||||| |•|| |
CCU      CU GCUA CA  GACCC CUAG A
   ACUCA  A    C  AG     C    C
---------------------------
AUUC->
---------------------------
```

ITS-2 rRNA secondary structure model of *Coccomyxa viridis* strain C9 (BC-7a) HE586536 *

Barcode 7a ITS2-G7

5.8S/LSU stem

```
---00000-000-0111-11
---12345-678-9012-34
---23442-156-2453-26
```

BC

```
GCC      C    G     U
   UGCCU AGU UCGG UU->
   |||||  |•• ||•| |•
   acgga uug aguc AG<-
gga      c    -     C
------------------------
->CACCC
------------------------
```

Helix I

```
1111--1
5678--9
6434--4
```

BC

```
    CA           -    U
UCGC  CCCCUUUCUC UGUC C
•|||  |||||||||| |||| |
GGCG  GGGGAAAGAG ACAG C
    C-           U    G
------------------------
AUC
------------------------
```

Helix II

```
22222222223
01234567890
65347774443
```

BC

```
    CUU       GU
UGGC   CCCGGUC  \
••||   |||||||  A
GUCG   GGGCCAG  /
    UCU       UU
------------------------
AAGCGCAGA
-------------------------------------------------------
```

Helix III

```
333-333-3334444--4444-------4-455--55-555--55566-6666--66667777
123-456-7890123--4567-------8-901--23-456--78901-2345--67890123
334-286-1343833--2444-------3-333--41-413--43361-3631--44822424
```

BC

```
   -   G       AU     UUCUAAU U    AA  A   UC      G    GC         C
GGC U-U AGCG-GG  UCCC        G GGG   CA CAG  CGGUA GUGA  CC-UUCUC \
||| |  • |||| ||  ||||        | |||   || |||  |||•| |•||  || ||||| U
CCG A-G UCGC-CC  AGGG        C CCC   GU GUC  GCCGU CGCU  GG-AAGAG /
   C   G       --     ------- -    AG  C   --      A    A-         C
-------------------------------------------------------
CAGCAG
---------------------------
```

Helix IV

```
   A---   C  GA          CA
GAG    CGG GU  CGGUGGUUCC  A
|||    ||| ||  ||•|||||||  |
CUC    GCC CA  GCUACCAAGG  U
   ACUA   C  AA          AA
---------------------------
AACCAUUC->
---------------------------
```

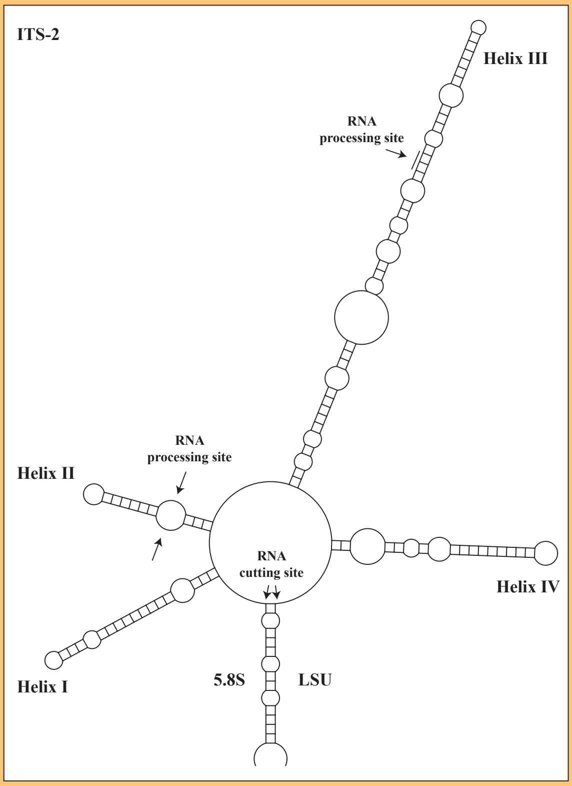

Barcode legend:

1 = A-U
2 = U-A
3 = G-C
4 = C-G
5 = G•U
6 = U•G
7 = mismatch
8 = deletion,single or unpaired bases

ITS-2 rRNA secondary structure model of *Coccomyxa sp.*
uncultured sample (BC-8) FJ553991

Barcode 8
ITS2-J

5.8S/LSU stem

```
---00000-000-0111-11
---12345-678-9012-34
---63442-154-2453-26
```

BC

```
GCC     C   G    U
   UGCCU AGC UCGG UU->
   •|||| |•| ||•| |•
   GCGGA UUG AGUC AG<-
AGA     C   -    C
------------------------
->ACACCC
------------------------
```

Helix I

```
11111
56789
64224
```

BC

```
     C       G
UCUUC UACACAU U
•|||| •|||||• |
GGAAG GUGUGUG G
     C       U
------------------------
ACC
------------------------
```

Helix II

```
22222222223
01234567890
65347774443
```

BC

```
    UGU      UU  G
UGGC   CCCGAU  UU C
••||   ||||||  || |
GUCG   GGGCUA  AA A
    CCU      CU  A
------------------------
AAGAACAGA
-------------------------------------------------------
```

Helix III

```
333--333-3334-444---44444----45-5555555--5566-666666667777
123--456-7890-123---45678----90-1234567--8901-234567890123
354--484-1343-233---42442----33-4114136--3361-343124888381
```

BC

```
   --   G    -   ACC     CUAA  G       CU    G            G
GGC  C-C AGCG UGG    CUCCU    GG CAACAGU  GGUA GCGAUC---G-A C
|•|  | | |||| |||    |||||    || ||||||•  ||•| ||||||    | | |
CUG  G-G UCGC ACC    GAGGA    CC GUUGUCG  CCGU CGCUAG---C-U A
   AA   -    U   A--     ----  G       --    A            G
-------------------------------------------------------
GCAAGC
---------------------------
```

Helix IV

```
   A
GGG A
||| |
CCC A
   A
---------------------------
AUGAAACAUUC->
---------------------------
```

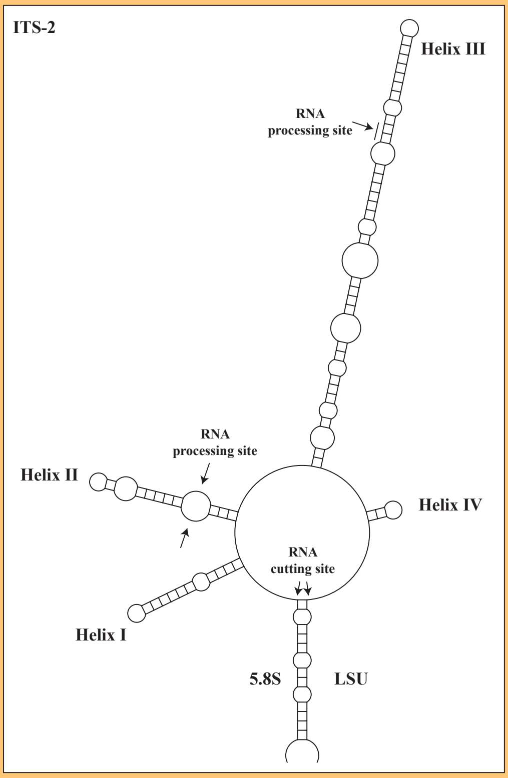

Barcode legend:

1 = A-U
2 = U-A
3 = G-C
4 = C-G
5 = G•U
6 = U•G
7 = mismatch
8 = deletion,single or unpaired bases

ITS-2 rRNA secondary structure model of *Coccomyxa sp.* strain T4 (BC-9a) HE586516

Barcode 9a ITS2-K1

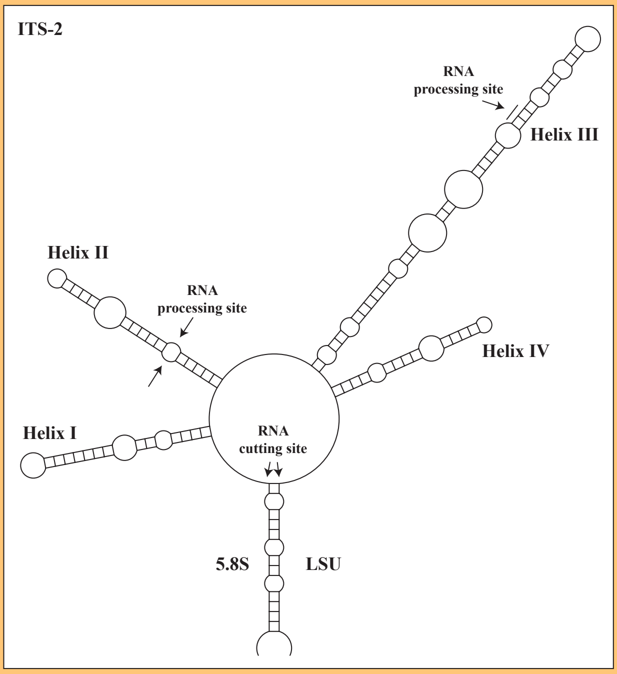

Barcode legend:

1 = A-U
2 = U-A
3 = G-C
4 = C-G
5 = G•U
6 = U•G
7 = mismatch
8 = deletion,single or unpaired bases

5.8S/LSU stem

```
---00000-000-0111-11
---12345-678-9012-34
---23442-154-2453-26   BC

GUC     C   G    C
   UGCCU AGC UCGG UU->
   ||||| |•| ||•| |•
   ACGGA UUG AGUC AG<-
GGA     C   -    C
------------------------
->CACCCCC
------------------------
```

Helix I

```
11111
56789
64344   BC

     -   C-        UU
UCGCC UCC  CUUUGUUU  U
•|||| |||  |•|||||•  |
GGCGG AGG  GGAACAAG  A
     U   AA        CC
------------------------
ACG
------------------------
```

Helix II

```
22222222223
01234567890
65343374443   BC

      U      GAA     C
UGGCGG CCCGGU   CUUUC U
••|||| ||||||   ||||| |
GUCGCC GGGCCA   GAAAG U
      U      ACA     C
------------------------
AAGACCAGA
-------------------------------------------------------
```

Helix III

```
33-3333-333444444444---4555---55555--5566-666-666667777
12-3456-789012345678---9012---34567--8901-234-567890123
33-4284-134115384448---3338---14136--3361-313-382488888   BC

  -    G       A   AUUC   ACAA      CU     G   C    GU
GG CU-C AGCAAGG CCC    MGG    ACAGU  GGUA GAG G-UC  C
|| || | |||||•| |||    |||    ||||•  ||•| ||| | ||  |
CC GA-G UCGUUUC GGG    CCC    UGUCG  CCGU CUC C-AG  U
  G    G       A   ----   GGC-      C-     A   -    AC
-------------------------------------------------------
G
---------------------------
```

M = C or A
(should be a G)

Helix IV

```
     C     UC     U
GAAGG GCUCC  GGUUA \
||||• |||||  ||||| G
CUUCU CGAGG  CCAAU /
     -     --     G
---------------------------
AUGAAAUAUUC->
---------------------------
```

ITS-2 rRNA secondary structure model of *Coccomyxa sp.* strain CCAP 216/25 (BC-9b) FR850476

Barcode 9b ITS2-K2

5.8S/LSU stem

```
---00000-000-0111-11
---12345-678-9012-34
---23442-154-2453-26
```

BC

```
GNC     C   G    C *
   UGCCU AGC UCGG Uu->
   ||||| |•| ||•| |•
   ACGGA UUG AGUC AG<-
GGA     C   -    C
------------------------
->CACCCCC
------------------------
```

N = here probably U
* = U is missing here

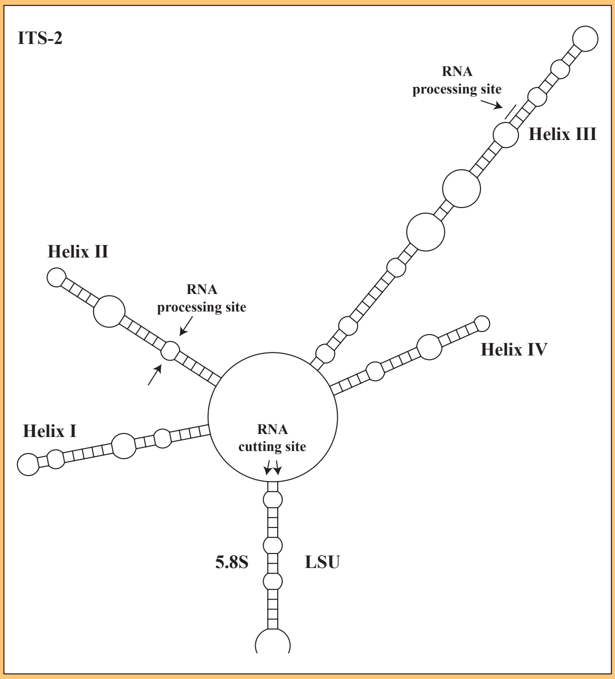

Helix I

```
11111
56789
64344
```

BC

```
     -   C-      C  CU
UCGCC UCC  CUUUGU UC  \
•|||| |||  |•|||| ||  A
GGCGG AGG  GGAACA AG  /
     U   AA      -  CC
------------------------
ACG
------------------------
```

Helix II

```
22222222223
01234567890
65343374443
```

BC

```
      U      GAA     C
UGGCGG CCCGGU   CUUUC U
••|||| ||||||   |•||| |
GUCGCC GGGCCA   GGAAG U
      U      ACA     C
------------------------
AAGACCAGA
-------------------------------------------------------
```

Barcode legend:

1 = A-U
2 = U-A
3 = G-C
4 = C-G
5 = G•U
6 = U•G
7 = mismatch
8 = deletion,single or unpaired bases

Helix III

```
33-3333-333444444444---4555---55555--5566-666-666667777
12-3456-789012345678---9012---34567--8901-234-567890123
33-4284-154115384448---3338---14136--3361-313-382488888
```

BC

```
  -    G       A   AUUC   ACAA     CU    G   C    GU
GG CU-C AGCAAGG CCC    GGG    ACAGU  GGUA GAG G-UC  C
|| || | |•|||•| |||    |||    ||||•  ||•| ||| | ||  |
CC GA-G UUGUUUC GGG    CCC    UGUCG  CCGU CUC C-AG  U
  G    G       A   ----   GGC-     C-    A   -    AC
-------------------------------------------------------
G
---------------------------
```

Helix IV

```
     C     UC     U
GAAGG GCUCC  GGUUA \
||||• ||||   ||||| G
CUUCU CGAGG  CCAAU /
     -     --     G
---------------------------
AUGAAAUAUUC->
---------------------------
```

ITS-2 rRNA secondary structure model of *Coccomyxa sp.* strain T1 (BC-10) HE586550

Barcode 10 ITS2-L1

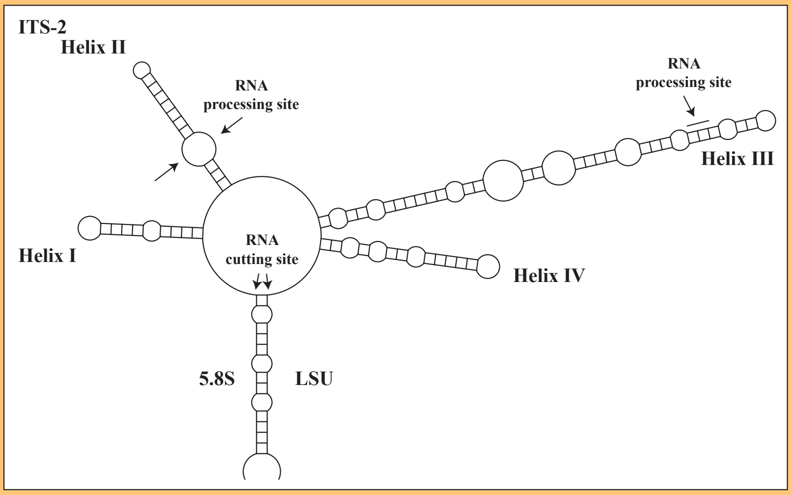

5.8S/LSU stem

```
---00000-000-0111-11
---12345-678-9012-34
```

```
---23442-154-2453-26
```

BC

```
GUC     C   G    C
   UGCCU AGC UCGG UU->
   ||||| |•| ||•| |•
   ACGGA UUG AGUC AG<-
GGA     C   -    C
------------------------
->CACCCUC
------------------------
```

Barcode legend:

1 = A-U
2 = U-A
3 = G-C
4 = C-G
5 = G•U
6 = U•G
7 = mismatch
8 = deletion,single or unpaired bases

Helix I

```
11111
56789
```

```
64344
```

BC

```
     C     UC
UCGCC CCCCG  \
•|||| |||||  A
GGCGG GGGGC  /
     C     AC
------------------------
ACA
------------------------
```

Helix II

```
22222222223
01234567890
```

```
65347774443
```

BC

```
    AGU        A
UGGC   CCCGGUCG \
••||   |||||||| U
GUCG   GGGCCAGC /
    CCU        C
------------------------
AAGACCAGA
-------------------------------------------------------
```

Helix III

```
33-3333-333444444444---4555--55555--5566-666666667777
12-3456-789012345678---9012--34567--8901-234567890123
```

```
33-4284-134115384448---3338--14136--3361-363184488884
```

BC

```
  -    G       A   AAUC   ACA     CU    G    -       G
GG CU-C AGCAAGG CCC     GGG   ACAGU  GGUA GUGA CC----C C
|| || | |||||•| |||     |||   ||||•  ||•| |•|| ||    | |
CC GA-G UCGUUUC GGG     CCC   UGUCG  CCGU CGCU GG----G A
  G    G       A   ----   GGC     --    A    A       A
-------------------------------------------------------
-
---------------------------
```

Helix IV

```
   A  C   G      AU
GGU GG GCG CCGGUU  \
|•| |• ||| ||||||  G
CUA CU CGC GGCCAA  /
   -  A   -      GG
---------------------------
UCAUUC->
---------------------------
```

ITS-2 rRNA secondary structure model of *Coccomyxa sp.* strain T3 (BC-10) HE586515

Barcode 10 ITS2-L1

5.8S/LSU stem

```
---00000-000-0111-11
---12345-678-9012-34
---23442-154-2453-26
```

BC

```
GUC      C    G     C
   UGCCU AGC UCGG UU->
   ||||| |•| ||•| |•
   ACGGA UUG AGUC AG<-
GGA      C    -     C
------------------------
->CACCCUC
------------------------
```

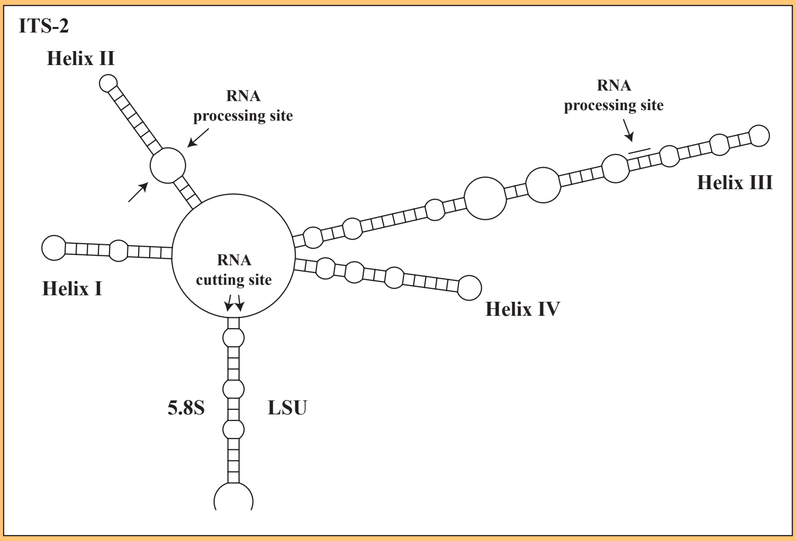

Helix I

```
11111
56789
64344
```

BC

```
     C      UC
UCGCC CCCCG   \
•|||| |||||    A
GGCGG GGGGC   /
     C      AC
------------------------
ACA
------------------------
```

Barcode legend:

1 = A-U
2 = U-A
3 = G-C
4 = C-G
5 = G•U
6 = U•G
7 = mismatch
8 = deletion,single or unpaired bases

Helix II

```
22222222223
01234567890
65347774443
```

BC

```
    AGU        A
UGGC   CCCGGUCG \
••||   |||||||| U
GUCG   GGGCCAGC /
    CCU        C
------------------------
AAGACCAGA
-------------------------------------------------------
```

Helix III

```
33-3333-333444444444---4555--55555--5566-666666667777
12-3456-789012345678---9012--34567--8901-234567890123
33-4284-134115384448---3338--14136--3361-363184488884
```

BC

```
  -    G       A   AAUC   ACA     CU    G    -      G
GG CU-C AGCAAGG CCC    GGG   ACAGU  GGUA GUGA CC----C C
|| || | |||||•| |||    |||   ||||•  ||•| |•|| ||    | |
CC GA-G UCGUUUC GGG    CCC   UGUCG  CCGU CGCU GG----G A
  G    G       A   ----   GGC     --    A    A      A
-------------------------------------------------------
-
---------------------------
```

Helix IV

```
   A  C   G      AU
GGU GG GCG CCGGUU  \
|•| |• ||| ||||||   G
CUA CU CGC GGCCAA  /
   -  A   -      GG
---------------------------
UCAUUC->
---------------------------
```

ITS-2 rRNA secondary structure model of *Coccomyxa sp.* strain ACCV1 (BC-10) HE617183

Barcode 10 ITS2-L2

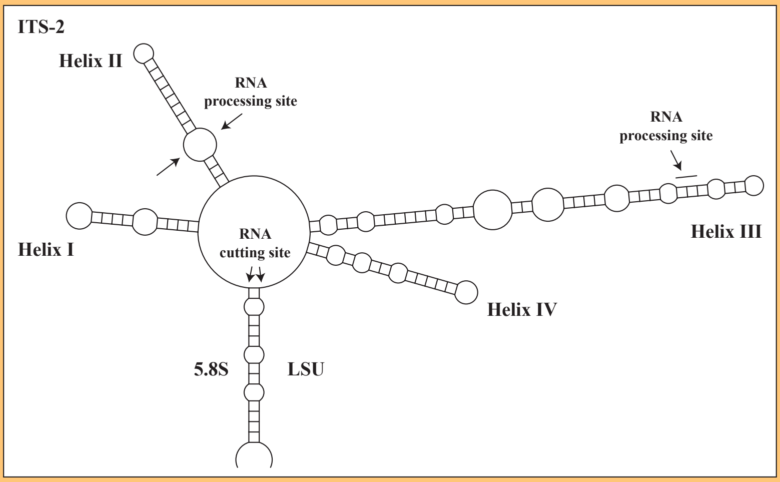

Barcode legend:

1 = A-U
2 = U-A
3 = G-C
4 = C-G
5 = G•U
6 = U•G
7 = mismatch
8 = deletion,single or unpaired bases

5.8S/LSU stem

```
---00000-000-0111-11
---12345-678-9012-34
---23442-154-2453-26   BC

GUC     C   G    C
   UGCCU AGC UCGG UU->
   ||||| |•| ||•| |•
   ACGGA UUG AGUC AG<-
GGA     C   -    C
------------------------
->CACCCUC
------------------------
```

Helix I

```
11111
56789
64344   BC

     C-     CU
UCGCC  CUCCC  U
•||||  |||||  |
GGCGG  GAGGG  C
     CA     UG
------------------------
ACA
------------------------
```

Helix II

```
22222222223
01234567890
65347774443   BC

    AGU         U
UGGC   CCCGGUCGC U
••||   |||||||•| |
GUCG   GGGCCAGUG C
    CCU         G
------------------------
AAGGCCAGA
-------------------------------------------------------
```

Helix III

```
33-3333-333444444444---4555--55555--5566-666666667777
12-3456-789012345678---9012--34567--8901-234567890123
33-4284-134115384448---3338--14136--3361-363184488884   BC

  -    G       A   AAUC   ACA     CU    G    -       G
GG CU-C AGCAAGG CCC    GGG   ACAGU  GGUA GUGA CC----C C
|| || | |||||•| |||    |||   ||||•  ||•| |•|| ||    | |
CC GA-G UCGUUUC GGG    CCC   UGUCG  CCGU CGCU GG----G A
  G    G       A   ----   GGC     --    A    A       A
-------------------------------------------------------
-
---------------------------
```

Helix IV

```
   A  C   U      AC
GGA GG GCG CCGGUU  \
•|| || ||| ||||||  G
UCU CC CGC GGCCAA  /
   A  C   -      GG
---------------------------
CAUUC->
---------------------------
```

ITS-2 rRNA secondary structure model of *Coccomyxa sp.*
strain UTEX SNO83 (BC-11) HE586506 *

Barcode 11
ITS2-M

5.8S/LSU stem

```
---00000-000-0111-11
---12345-678-9012-34
---23442-154-2453-26
```

BC

```
GCC     C   G    -
   UGCCU AGC UCGG UU->
   ||||| |•| ||•| |•
   acgga uug aguc ag<-
gga     c   -    c
------------------------
->CCACCC
------------------------
```

Helix I

```
1111-1
5678-9
6434-4
```

BC

```
    - -    -    UU
UCGC C UCUC CCAC  \
•||| | |||| ||||  C
GGCG G AGAG GGUG  /
    A A    U    GC
------------------------
ACC
------------------------
```

Helix II

```
22222222223
01234567890
65344374443
```

BC

```
      U       CA
UGGCCG CCCGAUC  \
••|||| |||||||  G
GUCGGC GGGCUAG  /
      U       CG
------------------------
AAGCGCAGA
-------------------------------------------------------
```

Helix III

```
333-333-333444-44444------4-455--55-555--55566-6666--66667777
123-456-789012-34567------8-901--23-456--78901-2345--67890123
334-286-134333-32444------3-333--41-413--43361-3631--43828484
```

BC

```
   -   G      A     UCCAAU C    AA  A    AC      G     GC        G
GGC U-U AGCGGG GUCCC      G GGG  CA CAG  CGGUA GUGA  CG-U-C-C G
||| | • |||||| |||||      | |||  || |||  |||•| |•||  || | | | |
CCG A-G UCGCCC CAGGG      C CCC  GU GUC  GCCGU CGCU  GC-A-G-G A
   C   G      -     ------ -    GG  C    --      A     A-        A
-------------------------------------------------------
GAGCA
---------------------------
```

Helix IV

```
   -  C   G   C    ---     G   G
GGA GA GGU CGG UC    GCGAC GGG C
||| || ||| ||| ||    ||||| ||| |
CCU CU CCA GCC AG    CGCUG CCC G
   A  A   -   -  CGA       A   A
---------------------------
AUuc->
---------------------------
```

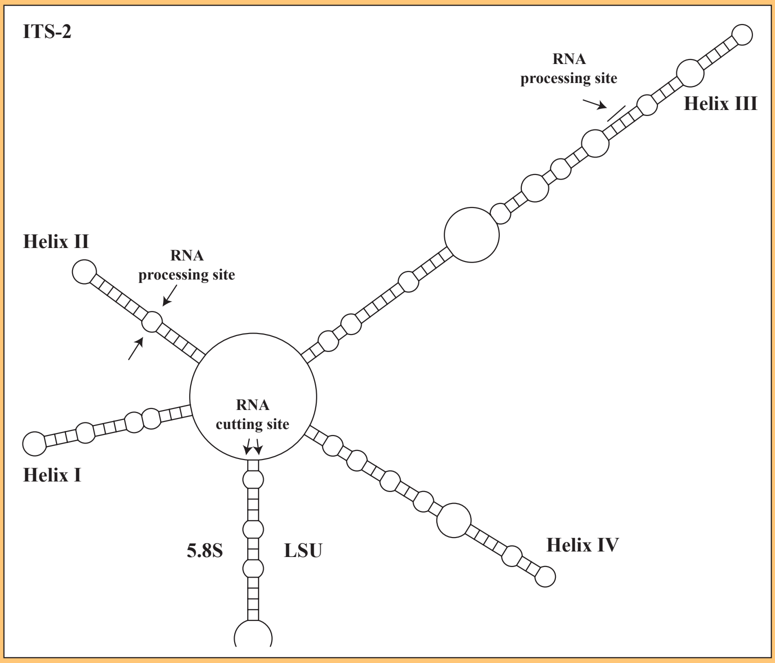

Barcode legend:

```
1 = A-U
2 = U-A
3 = G-C
4 = C-G
5 = G•U
6 = U•G
7 = mismatch
8 = deletion,single or unpaired bases
```

ITS-2 rRNA secondary structure model of *Coccomyxa sp.* strain C4 (BC-12) HE586508 *

Barcode 12 ITS2-N

5.8S/LSU stem

```
---00000-000-0111-11
---12345-678-9012-34
---23442-154-2453-26
```

BC

```
GCC      C    G     -
   UGCCU AGC UCGG UU->
   ||||| |•| ||•| |•
   acgga uug aguc aG<-
gga      c    -     c
------------------------
->CCACCC
------------------------
```

Helix I

```
1111-1
5678-9
6434-4
```

BC

```
    -      CUC-   A
UCGC CUCAC     CC U
•||| |||||     || |
GGCG GAGUG     GG C
    A      CGUU   C
------------------------
ACC
------------------------
```

Helix II

```
22222222223
01234567890
65344374443
```

BC

```
      U       CC
UGGCCG CCCGAUC  \
••|||| |||||||  U
GUCGGC GGGCUAG  /
      U       CG
------------------------
AAGCGCAGA
-------------------------------------------------------
```

Helix III

```
333-333-333444-44444-------4-455--55-555--55566-6666--6666-7777
123-456-789012-34567-------8-901--23-456--78901-2345--6789-0123
334-286-134333-32444-------3-333--41-413--43361-3631--4324-2424
```

BC

```
   -   G      A      UUCUAAC U   AA  A   AC      G     GC     C    U
GGC U-U AGCGGG GUCCC        G GGG  CA CAG  CGGUA GUGA  CGUC UCUC C
||| | • |||||| |||||        | |||  || |||  |||•| |•||  |||| |||| |
CCG A-G UCGCCC CAGGG        C CCC  GU GUC  GCCGU CGCU  GCAG AGAG C
   C   G      -      ------- -   GG  C   --      A     A-     -    G
-------------------------------------------------------
GAGCA
---------------------------
```

Helix IV

```
   GAC   G      --          G
GGA   GGU CGGCU  CGCGACGGGG A
|||   |•| ||||•  |||||||||| |
CCU   CUA GCCGG  GCGCUGCCCC G
   A--   A      CG          A
---------------------------
AUUC->
---------------------------
```

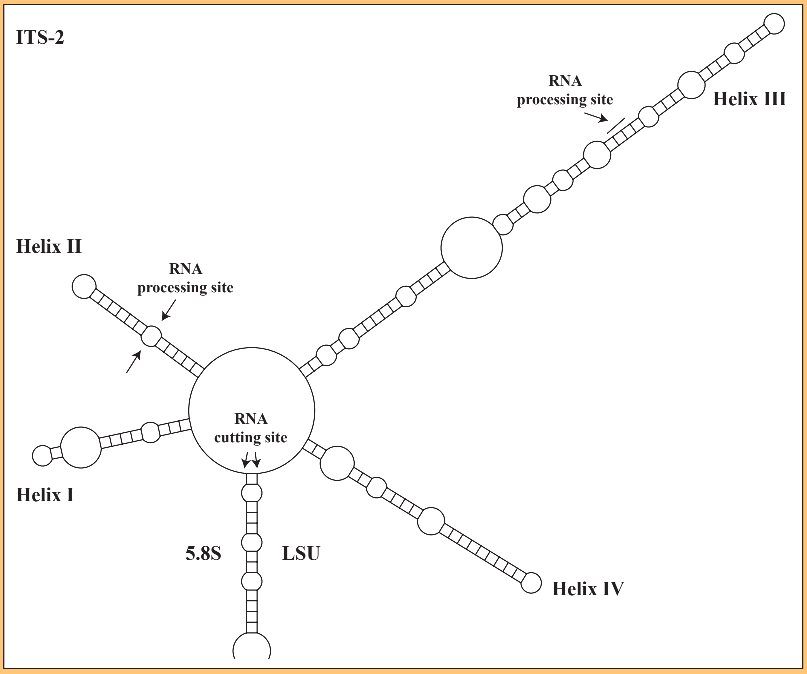


```
Barcode legend:

1 = A-U
2 = U-A
3 = G-C
4 = C-G
5 = G•U
6 = U•G
7 = mismatch
8 = deletion,single or unpaired bases
```
