# Supplementary figures and images for: Evaluating the Species Boundaries of Green Microalgae (Coccomyxa, Trebouxiophyceae, Chlorophyta) Using Integrative Taxonomy and DNA Barcoding with Further Implications for the Species Identification in Environmental Samples
Source: PLoS One. 2015 Jun 16;10(6):e0127838. doi: 10.1371/journal.pone.0127838 (PMC4469705; doi:10.1371/journal.pone.0127838)

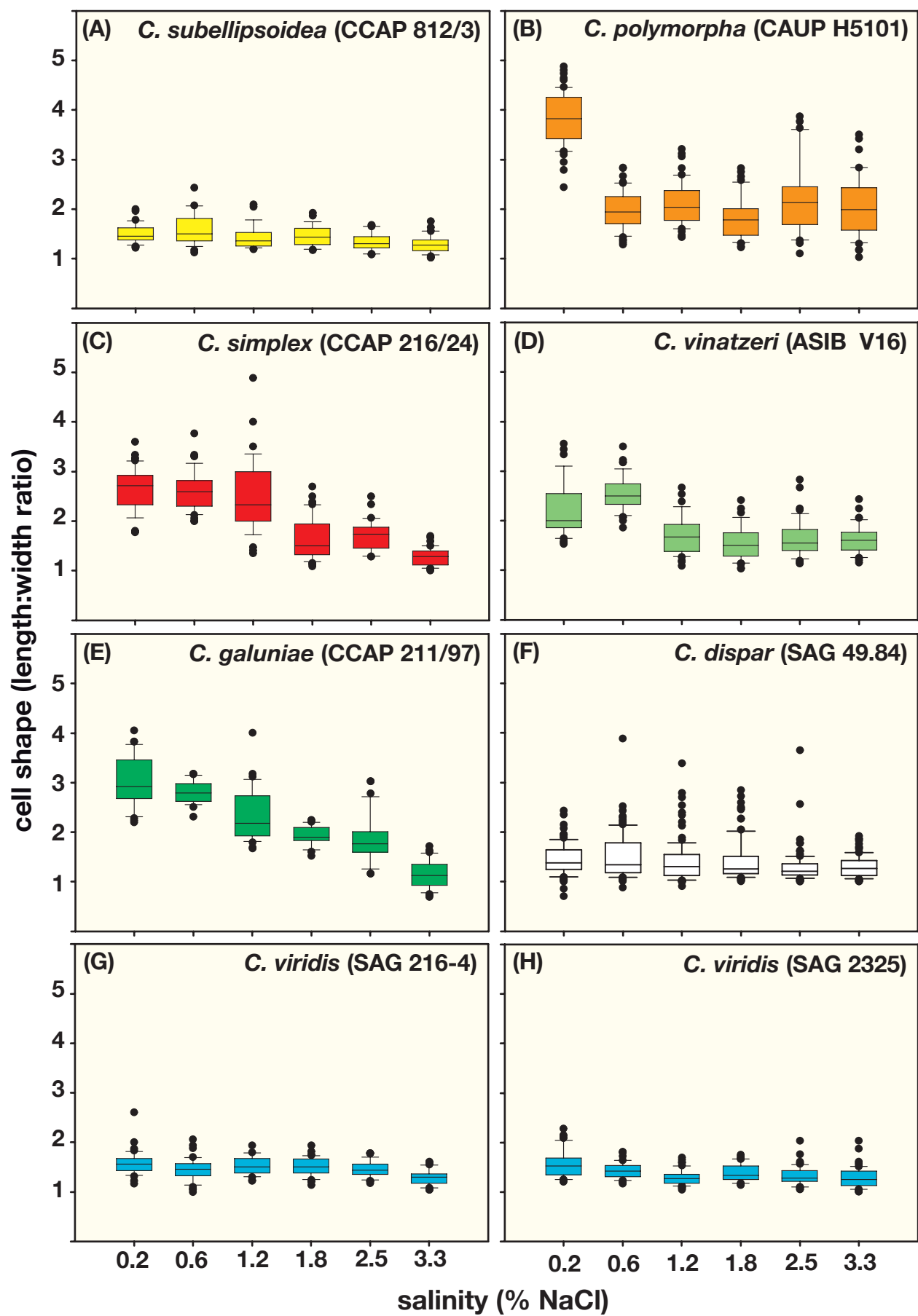

Supplement: S3 Fig — (PDF) [file pone.0127838.s003.pdf]

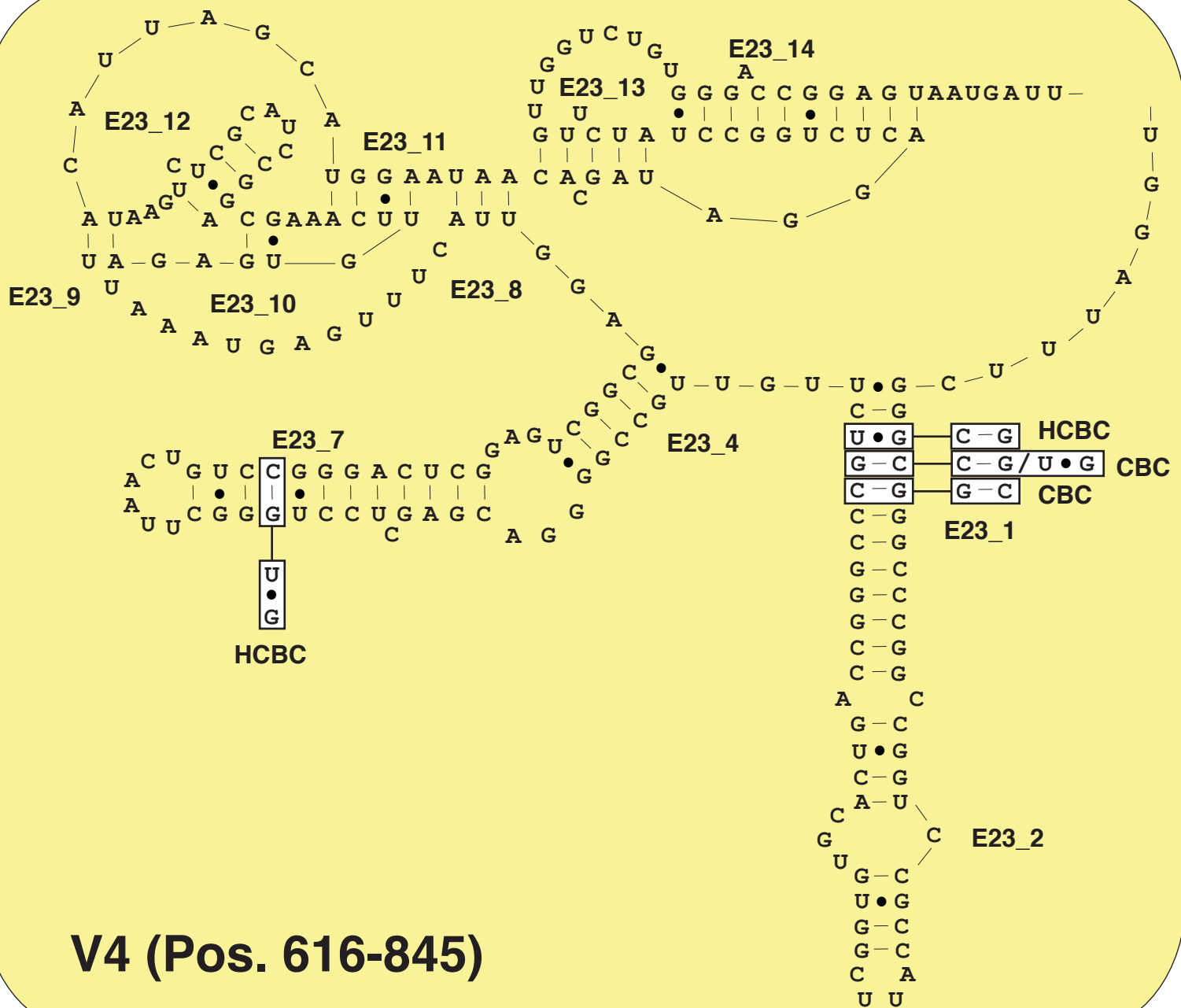

## V9 (Pos. 1631-1737)

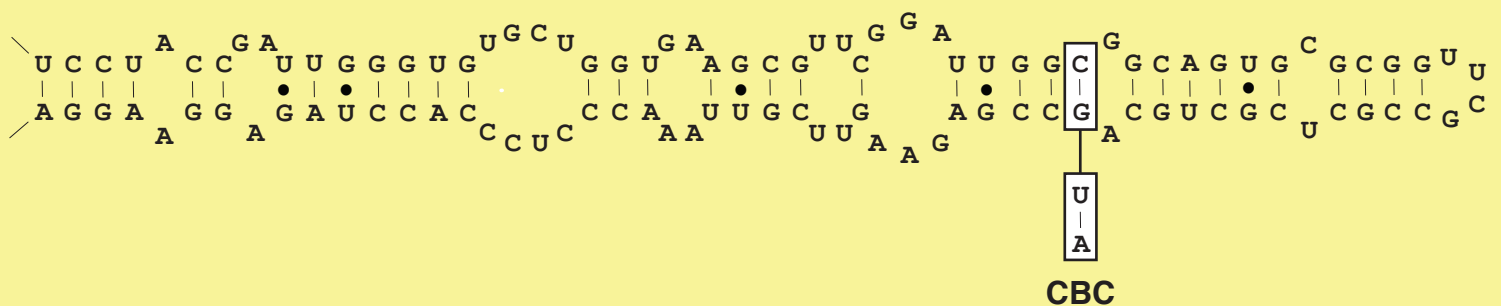

Supplement: S4 Fig — The numbering of the helices follows according to Wuyts et al. [83]. The CBCs/HCBCs characterizing Coccomyxa species in comparison to Elliptochloris bilobata and Hemichloris antarctica are highlighted in white boxes. (PDF) [file pone.0127838.s004.pdf]

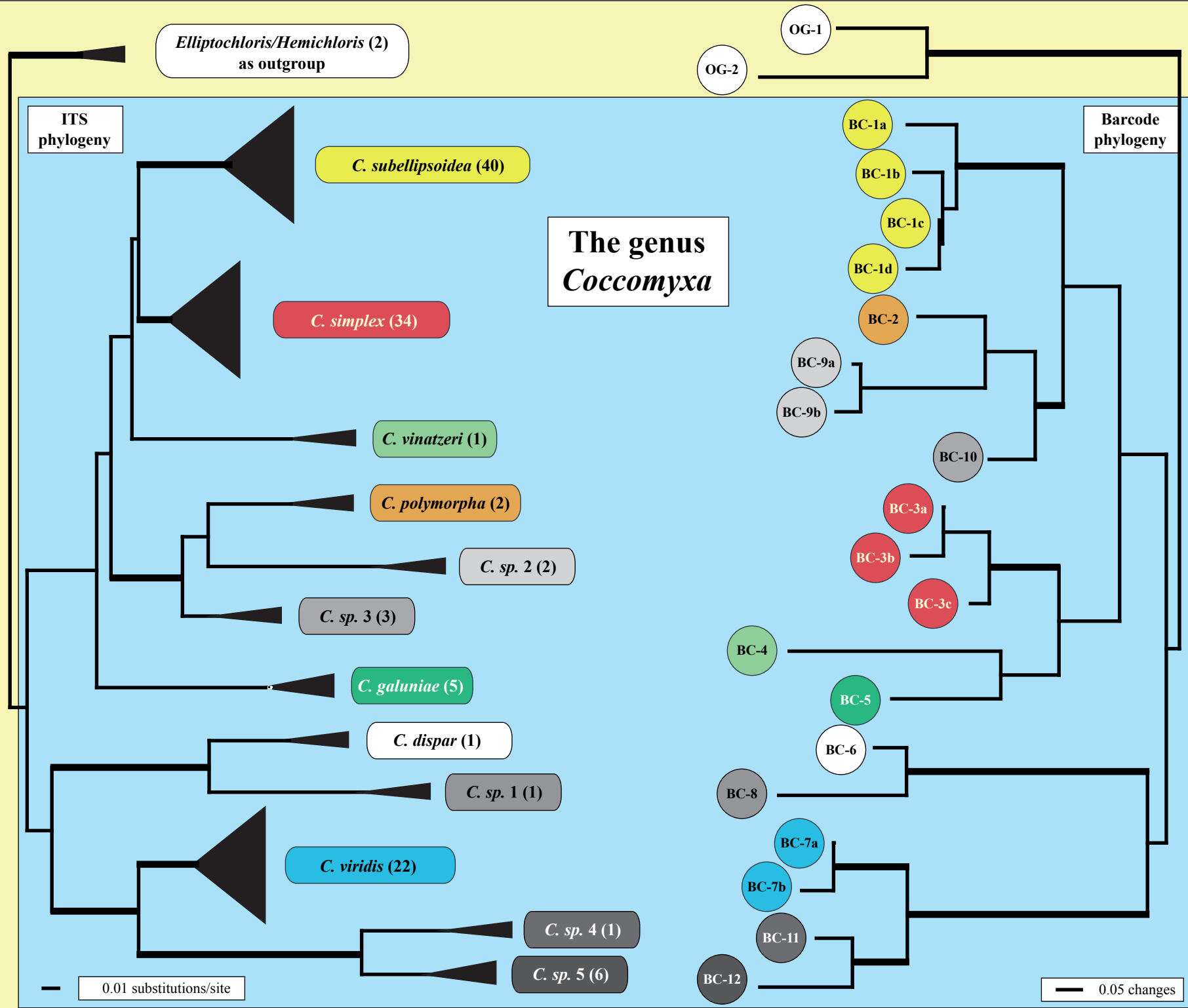

Supplement: S6 Fig — The phylogenetic tree shown on the left was inferred using the maximum likelihood method based on a data set of 895 aligned positions of 120 taxa using PAUP 4.0b10. The data set contains the sequences of the strains investigated in this study and the GenBank entries found with a BLAST search as described in the text. The number of records for each species is given in brackets after the color-coded species designation. The distance phylogeny of the number-coded ITS-2 DNA Barcodes is shown on the right. (PDF) [file pone.0127838.s006.pdf]

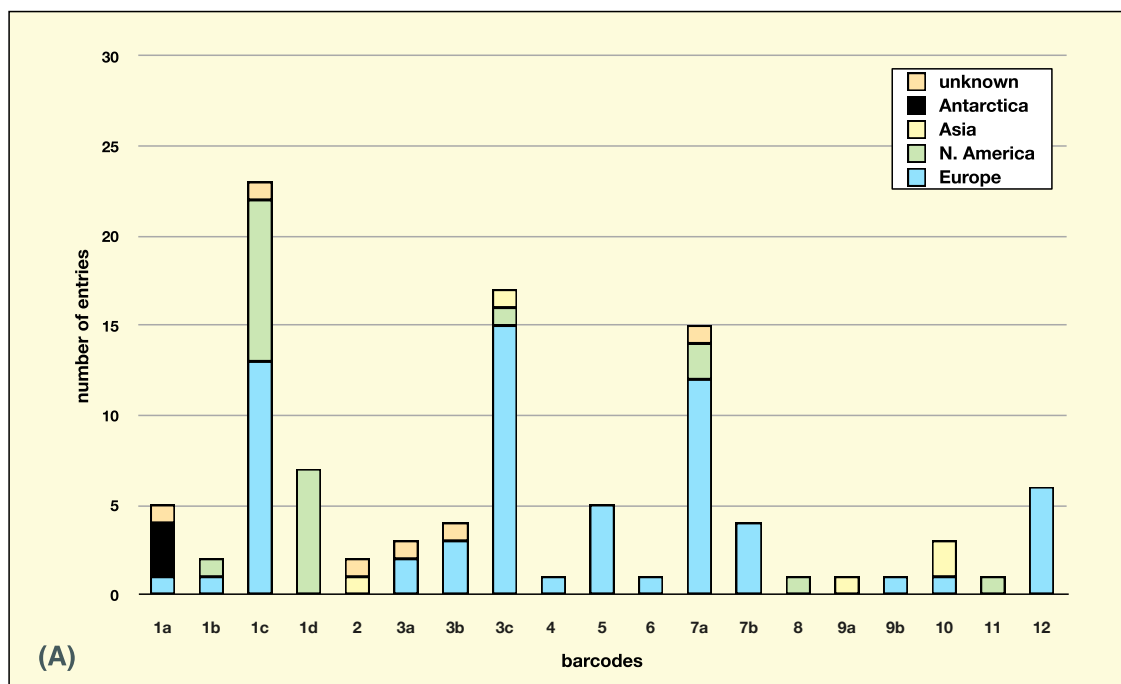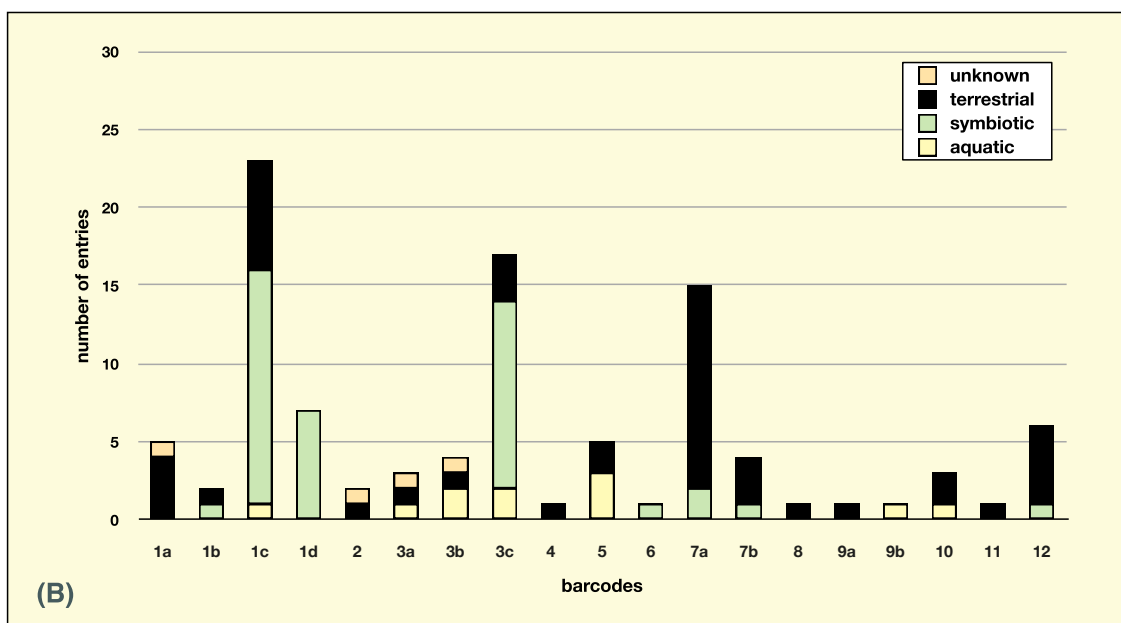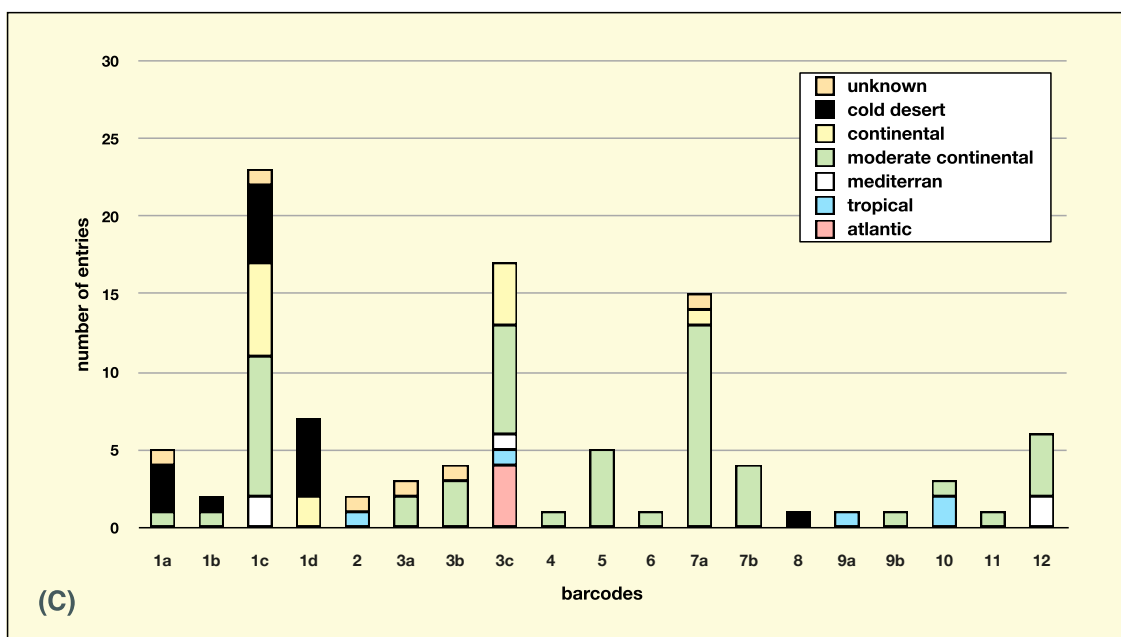

Supplement: S7 Fig — Detailed BLAST search results are summarized in the S6–S7 Tables. (PDF) [file pone.0127838.s007.pdf]
