# Supplementary material for: Evaluating the Species Boundaries of Green Microalgae (Coccomyxa, Trebouxiophyceae, Chlorophyta) Using Integrative Taxonomy and DNA Barcoding with Further Implications for the Species Identification in Environmental Samples
Source: PLoS One. 2015 Jun 16;10(6):e0127838. doi: 10.1371/journal.pone.0127838 (PMC4469705; doi:10.1371/journal.pone.0127838)
Supplement: S5 Fig — The phylogenetic tree shown was inferred using the maximum likelihood method based on a concatenated data set of 1780 aligned positions of 59 taxa using PAUP 4.0b10. For the analysis, the GTR+I+G model (base frequencies: A 0.2460, C 0.2225, G 0.2748, T 0.2567; rate matrix: A-C 1.0276, A-G 2.3668, A-T 0.9015, C-G 1.3952, C-T 5.5154, G-T 1.0000) with the proportion of invariable sites (I = 0.6279) and gamma distribution shape parameter (G = 0.5246) was chosen, which was calculated as the best model by Modeltest 3.7. The branches in bold are highly supported (Bayesian values > 0.95; bootstrap values > 70%) in all analyses. (PDF) [file pone.0127838.s005.pdf]

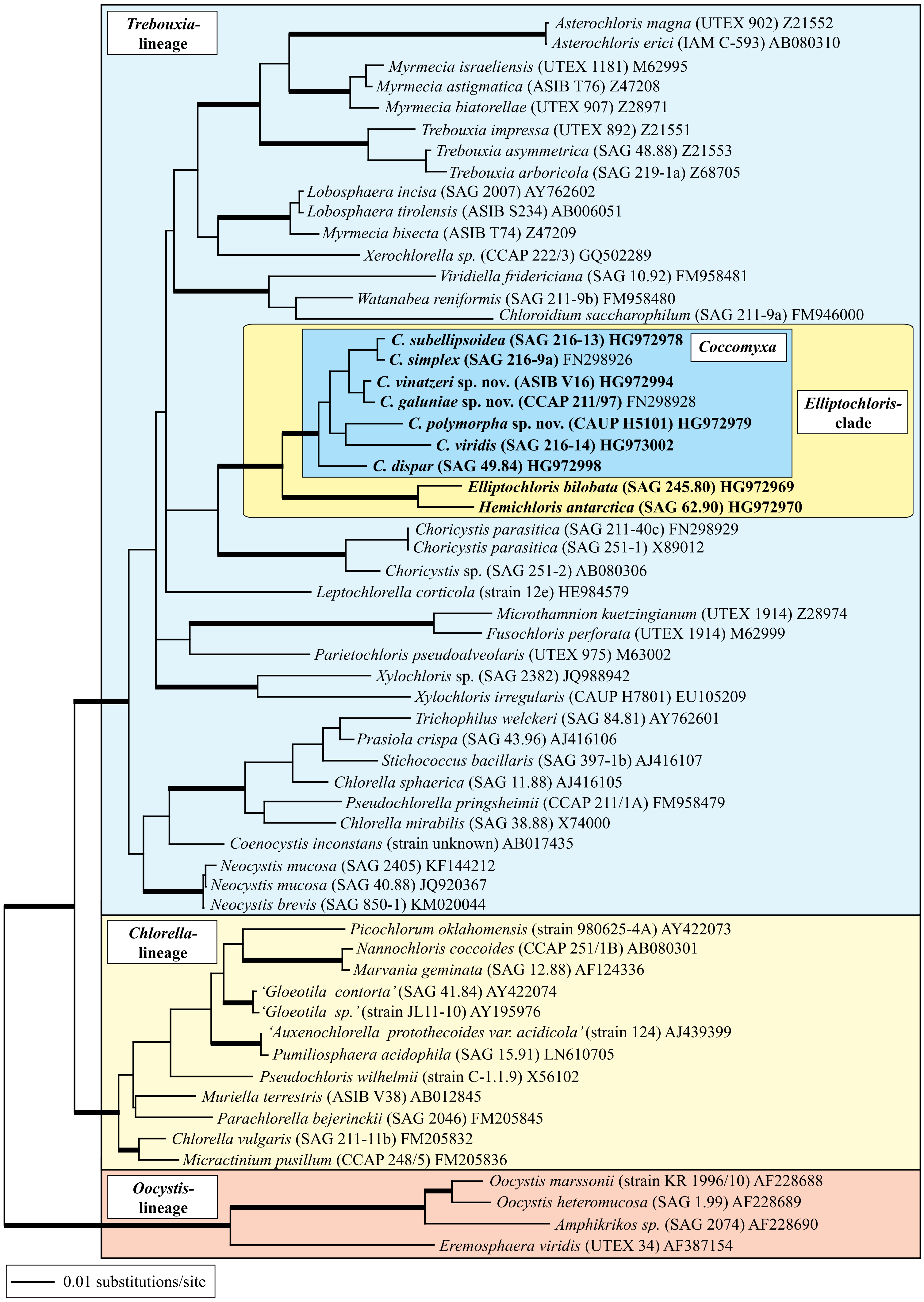

Trebouxia-lineage
Asterochloris magna (UTEX 902) Z21552
Asterochloris erici (IAM C-593) AB080310
Myrmecia israeliensis (UTEX 1181) M62995
Myrmecia astigmatica (ASIB T76) Z47208
Myrmecia biatorellae (UTEX 907) Z28971
Trebouxia impressa (UTEX 892) Z21551
Trebouxia asymmetrica (SAG 48.88) Z21553
Trebouxia arboricola (SAG 219-1a) Z68705
Lobosphaera incisa (SAG 2007) AY762602
Lobosphaera tirolensis (ASIB S234) AB006051
Myrmecia bisecta (ASIB T74) Z47209
Xerochlorella sp. (CCAP 222/3) GQ502289
Viridiella fridericiana (SAG 10.92) FM958481
Watanabea reniformis (SAG 211-9b) FM958480
Chloroidium saccharophilum (SAG 211-9a) FM946000
C. subellipsoidea (SAG 216-13) HG972978
Coccomyxa
C. simplex (SAG 216-9a) FN298926
C. vinatzeri sp. nov. (ASIB V16) HG972994
C. galuniae sp. nov. (CCAP 211/97) FN298928
Elliptochloris-clade
C. polymorpha sp. nov. (CAUP H5101) HG972979
C. viridis (SAG 216-14) HG973002
C. dispar (SAG 49.84) HG972998
Elliptochloris bilobata (SAG 245.80) HG972969
Hemichloris antarctica (SAG 62.90) HG972970
Choricystis parasitica (SAG 211-40c) FN298929
Choricystis parasitica (SAG 251-1) X89012
Choricystis sp. (SAG 251-2) AB080306
Leptochlorella corticola (strain 12e) HE984579
Microthamnion kuetzingianum (UTEX 1914) Z28974
Fusochloris perforata (UTEX 1914) M62999
Parietochloris pseudoalveolaris (UTEX 975) M63002
Xylochloris sp. (SAG 2382) JQ988942
Xylochloris irregularis (CAUP H7801) EU105209
Trichophilus welckeri (SAG 84.81) AY762601
Prasiola crispa (SAG 43.96) AJ416106
Stichococcus bacillaris (SAG 397-1b) AJ416107
Chlorella sphaerica (SAG 11.88) AJ416105
Pseudochlorella pringsheimii (CCAP 211/1A) FM958479
Chlorella mirabilis (SAG 38.88) X74000
Coenocystis inconstans (strain unknown) AB017435
Neocystis mucosa (SAG 2405) KF144212
Neocystis mucosa (SAG 40.88) JQ920367
Neocystis brevis (SAG 850-1) KM020044
Chlorella-lineage
Picochlorum oklahomensis (strain 980625-4A) AY422073
Nannochloris coccoides (CCAP 251/1B) AB080301
Marvania geminata (SAG 12.88) AF124336
'Gloeotila contorta' (SAG 41.84) AY422074
'Gloeotila sp.' (strain JL11-10) AY195976
'Auxenochlorella protothecoides var. acidicola' (strain 124) AJ439399
Pumiliosphaera acidophila (SAG 15.91) LN610705
Pseudochloris wilhelmii (strain C-1.1.9) X56102
Muriella terrestris (ASIB V38) AB012845
Parachlorella bejerinckii (SAG 2046) FM205845
Chlorella vulgaris (SAG 211-11b) FM205832
Micractinium pusillum (CCAP 248/5) FM205836
Oocystis-lineage
Oocystis marssonii (strain KR 1996/10) AF228688
Oocystis heteromucosa (SAG 1.99) AF228689
Amphikrikos sp. (SAG 2074) AF228690
Eremosphaera viridis (UTEX 34) AF387154
0.01 substitutions/site
