# Supplementary material for: Evaluating the Species Boundaries of Green Microalgae (Coccomyxa, Trebouxiophyceae, Chlorophyta) Using Integrative Taxonomy and DNA Barcoding with Further Implications for the Species Identification in Environmental Samples
Source: PLoS One. 2015 Jun 16;10(6):e0127838. doi: 10.1371/journal.pone.0127838 (PMC4469705; doi:10.1371/journal.pone.0127838)
Supplement: S1 Table — Accession numbers new in this study are marked in bold. (PDF) [file pone.0127838.s008.pdf]

Table S1. Strains used at the present study. New accession numbers in this study are marked in bold.

Strain	Accession number	Species name	Taxonomic revision	Habitat
SAG 245.80	**HG972969**	*Elliptochloris bilobata*	-	photobiont of the lichen *Catolechia wahlenbergii*
SAG 62.90	**HG972970**	*Hemichloris antarctica*	-	cryptoendolithic
SAG 49.84	**HG972998**	*Coccomyxa sp.*	*Coccomyxa dispar*	photobiont of the lichen *Multiclavula vernalis*
SAG 2253	**HG972996**	*Pseudoccomyxa closterioides*	*Coccomyxa galuniae*	forest soil
SAG 2254	**HG972997**	*Pseudoccomyxa closterioides*	*Coccomyxa galuniae*	forest soil
CCAP 211/97	FN298928	*Coccomyxa sp.*	*Coccomyxa galuniae*	maybe endosymbiont in *Stentor amethystinus*
CCAP 812/5	**HG972995**	*Pseudococcomyxa sp.*	*Coccomyxa galuniae*	phytoplankton
CAUP H5101	**HG972979**	*Choricystis chodatii*	*Coccomyxa polymorpha*	unknown
SAG 216-2	**HG972989**	*Coccomyxa chodatii*	*Coccomyxa simplex*	phytoplankton
SAG 216-3b	**HG972980**	*Coccomyxa elongata*	*Coccomyxa simplex*	unknown
SAG 216-3c	**HG972990**	*Coccomyxa elongata*	*Coccomyxa simplex*	unknown
SAG 216-5	**HG972982**	*Coccomyxa peltigerae*	*Coccomyxa simplex*	photobiont of the lichen *Peltigera aphtosa*
SAG 216-6	**HG972988**	*Coccomyxa peltigera-variolosae*	*Coccomyxa simplex*	photobiont of the lichen *Peltigera variolosa*
SAG 216-8	**HG972991**	*Coccomyxa rayssiae*	*Coccomyxa simplex*	unknown
SAG 216-9a	FN298926	*Coccomyxa simplex*	*Coccomyxa simplex*	from a culture of *Paramecium bursaria*
SAG 216-10	**HG972986**	*Coccomyxa solorina-bisporae*	*Coccomyxa simplex*	photobiont of the lichen *Solorina bispora*
SAG 216-11a	**HG972983**	*Coccomyxa solorina-croceae*	*Coccomyxa simplex*	photobiont of the lichen *Solorina crocea*
SAG 216-11b	**HG972984**	*Coccomyxa solorina-croceae*	*Coccomyxa simplex*	photobiont of the lichen *Solorina crocea*
SAG 216-12	**HG972987**	*Coccomyxa solorina-saccatae*	*Coccomyxa simplex*	photobiont of the lichen *Solorina saccata*
CCAP 216/15	**HG972985**	*Coccomyxa subellipsoidea*	*Coccomyxa simplex*	unknown
CCAP 216/24	FN298927	*Coccomyxa sp.*	*Coccomyxa simplex*	from a culture of *Paramecium bursaria*
CCAP 812/2A	**HG972992**	*Pseudococcomyxa simplex*	*Coccomyxa simplex*	phytoplankton
CCAP 812/2B	**HG972993**	*Pseudococcomyxa simplex*	*Coccomyxa simplex*	epiphyte of a Tobacco plant
CAUP H5107	**HG972981**	*Choricystis sp.*	*Coccomyxa simplex*	wet sandstone
Wien C20	**HG972975**	*Coccomyxa sp.*	*Coccomyxa subellipsoidea*	unknown
SAG 216-7	**HG972976**	*Coccomyxa pringsheimii*	*Coccomyxa subellipsoidea*	photobiont of the lichen *Omphalina sp.*
SAG 216-13	**HG972978**	*Coccomyxa subellipsoidea*	*Coccomyxa subellipsoidea*	photobiont of the lichen *Omphalina sp.*
SAG 69.80	**HG972977**	*Coccomyxa pringsheimii*	*Coccomyxa subellipsoidea*	photobiont of the lichen *Omphalina sp.*
CCAP 812/3	**HG972972**	*Pseudococcomyxa simplex*	*Coccomyxa subellipsoidea*	phytoplankton
NIES 2166	AGSI00000000	*Coccomyxa subellipsoidea*	*Coccomyxa subellipsoidea*	biofilm on stones
NIES 2252	**HG972973**	*Coccomyxa sp.*	*Coccomyxa subellipsoidea*	unknown
NIES 2353	**HG972971**	*Coccomyxa sp.*	*Coccomyxa subellipsoidea*	biofilm on stones
CAUP H5105	**HG972974**	*Choricystis sp.*	*Coccomyxa subellipsoidea*	wet sandstone
ASIB V16	**HG972994**	*Coccomyxa confluens*	*Coccomyxa vinatzeri*	soil
SAG 216-1	**HG972999**	*Coccomyxa arvernensis*	*Coccomyxa viridis*	epiphyte of the lichen *Acarospora fuscata*
SAG 216-4	**HG973001**	*Coccomyxa mucigena*	*Coccomyxa viridis*	photobiont of the lichen *Peltigera aphtosa*
SAG 216-14	**HG973002**	*Coccomyxa viridis*	*Coccomyxa viridis*	epiphyte of the lichen *Sphaerophorus coralloides*
SAG 2040	**HG973004**	*Coccomyxa sp.*	*Coccomyxa viridis*	roof tile
SAG 2104	**HG973003**	*Coccomyxa sp.*	*Coccomyxa viridis*	unknown
SAG 2127	**HG973005**	*Coccomyxa sp.*	*Coccomyxa viridis*	roof tile
SAG 2325	**HG973006**	*Coccomyxa sp.*	*Coccomyxa viridis*	epiphyte of the embryo of *Gingko biloba*
Wien C19	**HG973000**	*Coccomyxa sp.*	*Coccomyxa viridis*	bark of *Fagus silvatica*
CAUP H5103	**HG973007**	*Choricystis sp.*	*Coccomyxa viridis*	wet sandstone
