# Supplementary material for: Evaluating the Species Boundaries of Green Microalgae (Coccomyxa, Trebouxiophyceae, Chlorophyta) Using Integrative Taxonomy and DNA Barcoding with Further Implications for the Species Identification in Environmental Samples
Source: PLoS One. 2015 Jun 16;10(6):e0127838. doi: 10.1371/journal.pone.0127838 (PMC4469705; doi:10.1371/journal.pone.0127838)
Supplement: S2 Table — (PDF) [file pone.0127838.s009.pdf]

Table S2: Bayesian and Bootstrap support for each branch highlighted in Figure 2 using different phylogenetic methods described in detail in Material & Methods.

	SSU+ITS					
	PHASE	MrBayes	RAxML	ML	NJ	MP
Branch 1	1.00	1.00	100	100	100	100
Branch 2	1.00	1.00	100	100	100	100
Branch 3	1.00	1.00	100	100	100	100
Branch 4	1.00	1.00	100	100	100	100
Branch 5	1.00	1.00	100	100	100	100
Branch 6	-	-	58	87	86	75
Branch 7	-	0.95	71	76	52	53
Branch 8	1.00	1.00	65	51	94	75
Branch 9	1.00	1.00	100	100	100	100
Branch 10	-	-	-	-	-	-
Branch 11	0.98	1.00	58	50	-	-
Branch 12	1.00	1.00	84	91	95	94
Branch 13	1.00	1.00	83	86	72	75
Branch 14	1.00	1.00	100	100	100	100
Branch 15	1.00	1.00	100	100	100	100
Branch 16	1.00	1.00	74	92	100	92
Branch 17	1.00	1.00	100	100	100	100
Branch 18	1.00	1.00	100	100	100	100
Branch 19	-	-	-	-	-	-
Branch 20	1.00	1.00	100	100	100	100
Branch 21	-	-	80	55	80	73
Branch 22	1.00	1.00	100	100	100	100
Branch 23	1.00	1.00	100	100	100	100

Table S2: Bayesian and Bootstrap support for each branch highlighted in Figure 2 using different phylogenetic methods described in detail in Material & Methods.

	SSU					
	PHASE	MrBayes	RAxML	ML	NJ	MP
Branch 1	1.00	1.00	100	100	100	100
Branch 2	1.00	1.00	100	100	100	100
Branch 3	-	-	54	-	-	-
Branch 4	-	-	-	-	63	-
Branch 5	-	-	69	-	95	66
Branch 6	-	-	-	-	-	-
Branch 7	-	-	-	-	81	-
Branch 8	-	-	-	-	62	-
Branch 9	1.00	1.00	97	89	92	94
Branch 10	-	-	-	-	-	-
Branch 11	-	-	51	-	54	-
Branch 12	-	-	-	-	-	-
Branch 13	0.98	-	-	-	-	-
Branch 14	-	0.98	84	66	81	74
Branch 15	-	-	-	-	-	-
Branch 16	-	-	-	-	-	-
Branch 17	0.98	0.96	63	63	66	61
Branch 18	-	-	60	-	61	-
Branch 19	-	-	-	-	-	-
Branch 20	-	-	-	-	-	-
Branch 21	1.00	1.00	96	93	94	93
Branch 22	-	-	-	-	-	-
Branch 23	1.00	1.00	95	88	96	93

Table S2: Bayesian and Bootstrap support for each branch highlighted in Figure 2 using different phylogenetic methods described in detail in Material & Methods.

	ITS					
	PHASE	MrBayes	RAxML	ML	NJ	MP
Branch 1	1.00	1.00	100	100	99	99
Branch 2	1.00	1.00	100	100	100	100
Branch 3	1.00	1.00	100	100	100	100
Branch 4	1.00	1.00	100	99	99	100
Branch 5	1.00	1.00	100	100	100	100
Branch 6	-	-	74	57	79	80
Branch 7	-	0.99	79	77	-	-
Branch 8	0.99	-	57	56	87	81
Branch 9	1.00	1.00	100	100	100	100
Branch 10	-	-	-	-	-	-
Branch 11	-	-	-	-	-	-
Branch 12	1.00	1.00	66	78	84	92
Branch 13	-	-	-	56	-	60
Branch 14	1.00	1.00	100	100	100	100
Branch 15	1.00	1.00	100	100	100	100
Branch 16	1.00	1.00	83	90	100	97
Branch 17	1.00	1.00	94	99	100	100
Branch 18	1.00	1.00	100	100	100	100
Branch 19	-	-	-	-	-	-
Branch 20	1.00	1.00	99	100	100	100
Branch 21	-	-	-	-	51	55
Branch 22	1.00	1.00	100	100	100	100
Branch 23	1.00	1.00	100	100	100	100
