# Supplementary material for: Evaluating the Species Boundaries of Green Microalgae (Coccomyxa, Trebouxiophyceae, Chlorophyta) Using Integrative Taxonomy and DNA Barcoding with Further Implications for the Species Identification in Environmental Samples
Source: PLoS One. 2015 Jun 16;10(6):e0127838. doi: 10.1371/journal.pone.0127838 (PMC4469705; doi:10.1371/journal.pone.0127838)
Supplement: S3 Table — The user-defined trees were generated manually in TreeView. The log-likelihood values of each tree were calculated in PAUP. The observed difference in-lnL (Obs) and p-values of the approximately unbiased test (AU), unweighted (KH) and weighted (wKH) Kishino-Hasegawa test, and unweighted (SH) and weighted (wSH) Shimodaira-Hasegawa test were calculated in CONSEL. P-values < 0.05 indicate a significant rejection at 5% level and marked with an asterisk. (PDF) [file pone.0127838.s010.pdf]

Table S3: Topology tests upon eight user-defined trees derived from Figure 2 (= best tree), with a focus on the generic and species concept within the genus *Coccomyxa*

User-defined trees modified in TreeView	Obs	AU	KH	SH	wKH	wSH
**Tree 1 = best tree (Figure 2)**	-2.7	**0.964**	0.853	1.000	0.853	1.000
**Tree 2 = collapsed clade (branch 1); polyphyly of *Coccomyxa***	7765.3	**< 0.001***	0*	0*	0*	0*
**Tree 3 = collapsed branch 2; polyphyly of *C. viridis***	39.7	**< 0.001***	< 0.001*	< 0.001*	< 0.001*	0.001*
**Tree 4 = collapsed branch 12; polyphyly of *C. simplex***	8.0	**0.046***	0.060	0.732	0.060	0.215
**Tree 5 = collapsed branch 20; polyphyly of *C. subellipsoidea***	15.2	**0.011***	0.018*	0.595	0.018*	0.061
**Tree 6 = collapsed branch 11**	26.5	**0.001***	0.008*	0.505	0.008*	0.021*
**Tree 7 = collapsed branch 7**	3.8	**0.119**	0.119	0.842	0.119	0.435
**Tree 8 = collapsed branch 8**	2.7	**0.188**	0.147	0.917	0.147	0.576
**Tree 9 = collapsed branch 10**	26.5	**0.001***	0.008*	0.505	0.008*	0.021*

The user-defined trees were generated manually in TreeView. The log-likelihood values of each tree were calculated in PAUP. The observed difference in -lnL (Obs), and p-values of the approximately unbiased test (AU), unweighted (KH) and weighted (wKH) Kishino-Hasegawa test, and unweighted (SH) and weighted (wSH) Shimodaira-Hasegawa test were calculated in CONSEL. P-values < 0.05 indicate a significant rejection at 5% level and marked with an asterisk.
