# Supplementary material for: Evaluating the Species Boundaries of Green Microalgae (Coccomyxa, Trebouxiophyceae, Chlorophyta) Using Integrative Taxonomy and DNA Barcoding with Further Implications for the Species Identification in Environmental Samples
Source: PLoS One. 2015 Jun 16;10(6):e0127838. doi: 10.1371/journal.pone.0127838 (PMC4469705; doi:10.1371/journal.pone.0127838)
Supplement: S4 Table — Upper right corner = number of CBCs; lower left corner = number of HCBCs. (A) V4 region of SSU rDNA, (B) V9 region of SSU rDNA, and (C) ITS-2 DNA barcode (including the newly found barcodes of the BLAST search). (PDF) [file pone.0127838.s011.pdf]

Table S4A: Pairwise comparison of the V4 region (SSU) among the *Coccomyxa* species.
Upper right corner = number of CBCs; lower left corner = number of HCBCs.

species	*Ellipt.*	*Hemi.*	*C. subellipsoidea*		*C. polymorpha*	*C. simplex*			*C. vinatzeri*	*C. galuniae*	*C. dispar*	*C. viridis*
**POP-ID**	**V4-OG1**	**V4-OG2**	**V4-A1**	**V4-A2**	**V4-B**	**V4-C1**	**V4-C2**	**V4-C3**	**V4-D**	**V4-E**	**V4-F**	**V4-G**
**V4-OG1**		1	3	3	4	3	3	3	3	3	3	3
**V4-OG2**	1		2	2	3	2	2	2	2	2	2	2
**V4-A1**	2	2		0	1	0	0	0	0	0	0	0
**V4-A2**	2	2	0		1	0	0	0	0	0	0	0
**V4-B**	2	2	0	0		0	0	0	1	1	1	1
**V4-C1**	2	2	0	0	1		0	0	0	0	0	0
**V4-C2**	2	2	0	0	1	0		0	0	0	0	0
**V4-C3**	2	2	0	0	1	0	0		0	0	0	0
**V4-D**	2	2	0	0	0	0	0	0		0	0	0
**V4-E**	2	2	0	0	0	0	0	0	0		0	0
**V4-F**	2	2	0	0	0	0	0	0	0	0		0
**V4-G**	4	4	2	2	2	2	2	2	2	2	2	

Table S4B: Pairwise comparison of the V9 region (SSU) among the *Coccomyxa* species.
Upper right corner = number of CBCs; lower left corner = number of HCBCs.

species	*Ellipt.*	*Hemi.*	*C. sub./sim.*	*C. polymorpha*	*C. vinatzeri*	*C. galuniae*	*C. dispar*	*C. viridis*	
POP-ID	V9-OG1	V9-OG2	V9-A	V9-B	V9-C	V9-D	V9-E	V9-F1	V9-F2
V9-OG1		4	3	2	4	4	3	2	3
V9-OG2	0		5	5	5	5	5	5	5
V9-A	1	0		1	0	0	1	2	2
V9-B	0	0	2		2	2	2	0	0
V9-C	0	0	1	2		0	1	3	3
V9-D	1	0	3	3	2		1	3	3
V9-E	1	0	2	0	3	5		3	2
V9-F1	1	0	1	2	1	2	0		0
V9-F2	1	0	1	2	1	2	1	0	

Table S4C: Pairwise comparison of ITS-2 Barcode region among the *Coccomyxa* species. Upper right corner = number of CBCs; lower left corner = number of HCBCs.

species	*Ellipt.*	*Hemi.*	*C. subellipsoidea*				*C. polymorpha*	*C. simplex*			*C. vinatzeri*	*C. galuniae*	*C. dispar*	*C. viridis*		*C. sp. 1*	*C. sp. 2*		*C. sp. 3*	*C. sp. 4*	*C. sp. 5*
**POP-ID**	**OG-1**	**OG-2**	**BC-1a**	**BC-1b**	**BC-1c**	**BC-1d**	**BC-2**	**BC-3a**	**BC-3b**	**BC-3c**	**BC-4**	**BC-5**	**BC-6**	**BC-7a**	**BC-7b**	**BC-8**	**BC-9a**	**BC-9b**	**BC-10**	**BC-11**	**BC-12**
**OG-1**		6	6	7	7	7	5	6	6	5	6	6	5	3	3	6	6	6	4	4	4
**OG-2**	0		8	9	9	9	7	8	8	7	8	9	7	6	6	8	9	9	7	7	7
**BC-1a**	5	6		0	0	0	2	5	5	4	5	4	7	5	5	7	3	3	1	6	7
**BC-1b**	5	5	2		0	0	3	5	5	4	5	5	8	5	5	8	4	4	2	6	7
**BC-1c**	4	5	1	1		0	3	6	6	5	6	5	8	5	5	8	4	4	2	6	7
**BC-1d**	5	6	2	2	1		3	6	6	5	6	5	8	5	5	8	4	4	2	6	7
**BC-2**	3	4	2	2	1	2		6	6	5	6	5	6	3	3	9	2	2	1	4	5
**BC-3a**	3	4	2	2	1	2	2		0	0	1	3	8	4	4	9	7	7	4	6	7
**BC-3b**	3	4	2	2	1	2	2	0		0	1	3	8	4	4	9	7	7	4	6	7
**BC-3c**	3	4	2	2	1	2	2	0	0		1	3	7	3	3	8	6	6	3	5	6
**BC-4**	5	6	2	2	1	2	3	3	3	3		4	10	8	8	11	6	6	4	9	10
**BC-5**	4	5	2	2	1	2	2	2	2	2	1		6	6	6	7	5	5	4	7	7
**BC-6**	3	4	2	2	1	2	2	2	2	2	3	2		10	10	3	8	8	7	11	11
**BC-7a**	7	7	2	4	3	4	4	3	3	3	4	3	4		0	11	4	4	1	1	2
**BC-7b**	7	7	2	4	3	4	4	3	3	3	4	3	4	0		11	4	4	1	1	2
**BC-8**	5	6	4	4	3	4	4	4	4	4	4	4	2	6	6		10	10	8	13	13
**BC-9a**	4	4	2	2	1	2	2	2	2	2	2	2	1	4	4	3		0	3	6	7
**BC-9b**	5	5	3	2	2	3	3	3	3	3	3	3	2	5	5	4	1		3	6	7
**BC-10**	5	5	2	2	1	2	2	2	2	2	3	2	2	4	4	4	0	1		3	4
**BC-11**	7	7	2	4	3	4	4	3	3	3	4	2	4	0	0	6	4	5	4		1
**BC-12**	7	2	2	4	3	4	4	3	3	3	4	2	4	0	0	6	4	5	4	0	
