# Supplementary material for: Evaluating the Species Boundaries of Green Microalgae (Coccomyxa, Trebouxiophyceae, Chlorophyta) Using Integrative Taxonomy and DNA Barcoding with Further Implications for the Species Identification in Environmental Samples
Source: PLoS One. 2015 Jun 16;10(6):e0127838. doi: 10.1371/journal.pone.0127838 (PMC4469705; doi:10.1371/journal.pone.0127838)
Supplement: S5 Table — (PDF) [file pone.0127838.s012.pdf]

Table S5: Salinity dependent sensitivity in percentage among *Coccomyxa* species calculated by the decline of the growth rate from optimal (0.2 or 0.6% NaCl) to pessimal conditions (1.8, 2.5, or 3.3% NaCl, respectively).

species	strain	BC	decline of growth rate	relative salinity sensitivity (%)
C. subellipsoidea	NIES 2166	1a	-0.0246	98.0
	NIES 2252	1a	-0.0094	37.5
	CCAP 812/3	1a	-0.0158	63.0
	SAG 216-7	1c	-0.0085	33.9
	SAG 69.80	1c	-0.0130	51.8
	SAG 216-13	1c	-0.0175	69.8
C. polymorpha	CAUP H5101	2	-0.0251	100.0
C. simplex	SAG 216-3b	3a	-0.0072	28.6
	SAG 216-2	3b	-0.0121	48.3
	SAG 216-3c	3b	-0.0147	58.7
	SAG 216-8	3b	-0.0095	37.9
	SAG 216-9a	3b	-0.0118	47.1
	SAG 216-5	3c	-0.0067	26.7
	SAG 216-11a	3c	-0.0062	24.9
	SAG 216-11b	3c	-0.0055	21.9
	SAG 216-6	3c	-0.0052	20.7
	SAG 216-10	3c	-0.0041	16.3
	SAG 216-12	3c	-0.0014	5.7
	CCAP 216/15	3c	-0.0052	20.8
	CCAP 216/24	3c	-0.0116	46.4
	CCAP 812/2A	3c	-0.0111	44.3
	CCAP 812/2B	3c	-0.0029	11.6
C. vinatzeri	ASIB V16	4	-0.0083	33.1
C. galuniae	CCAP 211/97	5	-0.0151	60.2
	CCAP 812/5	5	-0.0116	46.3
	SAG 2253	5	-0.0073	29.1
	SAG 2254	5	-0.0076	30.4
C. dispar	SAG 49.84	6	-0.0107	42.5
C. viridis	SAG 216-1	7a	-0.0095	38.0
	SAG 216-4	7a	-0.0045	17.9
	SAG 216-14	7a	-0.0036	14.4
	SAG 2104	7a	-0.0022	8.8
	SAG 2040	7b	-0.0076	30.3
	SAG 2127	7b	-0.0092	36.7
	SAG 2325	7b	-0.0057	22.5
