# Supplementary material for: Evaluating the Species Boundaries of Green Microalgae (Coccomyxa, Trebouxiophyceae, Chlorophyta) Using Integrative Taxonomy and DNA Barcoding with Further Implications for the Species Identification in Environmental Samples
Source: PLoS One. 2015 Jun 16;10(6):e0127838. doi: 10.1371/journal.pone.0127838 (PMC4469705; doi:10.1371/journal.pone.0127838)
Supplement: S6 Table — (A) V4 region of SSU rDNA, (B) V9 region of SSU rDNA, and (C) ITS-2 DNA barcode. The grade of identities from 97–100% are color-coded. The designation after the accession number showed 100% identity to one of the strain in our study, the number in brackets recognized base differences in the sequences compared to the Coccomyxa species in this study, which are explained in S7 Table. The designations of the different haplotypes and ITS-2 Barcode followed those of S7 Table. (PDF) [file pone.0127838.s013.pdf]

Table S6A: BLAST N search results of the different types of V4 region (SSU) among the *Coccomyxa* species (100% coverage; 97-100% identity).

C. subellipsoidea		*C. polymorpha*	*C. simplex*			*C. vinatzeri*	*C. galuniae*	*C. dispar*	*C. viridis*
V4-A1	V4-A2	V4-B	V4-C1	V4-C2	V4-C3	V4-D	V4-E	V4-F	V4-G
AB742451 V4-A1	JQ315652 V4-A2	HE586514 (11)	JQ315652 V4-A2	HE586513 V4-C2	FN298927 V4-C3	HE586513 V4-C2	HE586512 V4-E	HE586512 V4-E	JX169832 V4-G
AB488787 V4-A1	HE586517 V4-A2	HE586515 (4)	HE586517 V4-A2	HE586504 V4-C2	AB260896 V4-C3	HE586504 V4-C2	HE586505 V4-E	HE586505 V4-E	HE586519 V4-G
JQ315652 V4-A2	HE586511 V4-A2	HE617184 (7)	HE586511 V4-A2	HQ317304 V4-C2	AM743096 V4-C3	HQ317304 V4-C2	HQ287928 V4-E	HQ287928 V4-E	HE586509 V4-G
HE586517 V4-A2	HE586510 V4-A2	HE617183 (7)	HE586510 V4-A2	FN298926 V4-C2	HE586513 V4-C2	FN298926 V4-C2	FN298928 V4-E	FN298928 V4-E	HE586508 V4-G
HE586511 V4-A2	GQ122371 V4-A2	KC155324 (7)	GQ122371 V4-A2	FJ648514 V4-C2	HE586504 V4-C2	FJ648514 V4-C2	HE586513 V4-C2	HE586518 (2)	HE586507 V4-G
HE586510 V4-A2	AY762603 V4-A2	JQ315652 V4-A2	AY762603 V4-A2	FJ648512 V4-C2	FR865679 (5)	FJ648512 V4-C2	HE586504 V4-C2	HE586513 V4-C2	HE586506 V4-G
GQ122371 V4-A2	FR865679 (5)	KC155323 (1)	HE586513 V4-C2	FJ946891 V4-C2	HQ317304 V4-C2	FJ946891 V4-C2	HQ317304 V4-C2	HE586504 V4-C2	JQ717057 V4-G
AY762603 V4-A2	HE586518 (2)	HE586517 V4-A2	HE586504 V4-C2	FJ592491 V4-C2	FN298926 V4-C2	FJ592491 V4-C2	FN298926 V4-C2	JQ411021 (2)	JN573865 V4-G
KC155323 (1)	JQ411021 (2)	HE586516 (3)	FR865679 (5)	FJ592486 V4-C2	FJ648514 V4-C2	FJ592486 V4-C2	FJ648514 V4-C2	HQ317304 V4-C2	FR865588 V4-G
FR865679 (5)	KC155323 (1)	HE586511 V4-A2	HQ317304 V4-C2	FJ592339 V4-C2	FJ648512 V4-C2	FJ592339 V4-C2	FJ648512 V4-C2	FN298927 V4-C3	HQ287296 V4-G
HE586518 (2)	HE586513 V4-C2	HE586510 V4-A2	FN298927 V4-C3	FJ592333 V4-C2	FJ946891 V4-C2	FJ592333 V4-C2	FJ946891 V4-C2	FN298926 V4-C2	HQ287295 V4-G
HE586516 ((3)	HE586504 V4-C2	GQ122371 V4-A2	FN298926 V4-C2	FN597599 (8)	FJ592491 V4-C2	HE586512 V4-E	FJ592491 V4-C2	FJ648514 V4-C2	HQ287294 V4-G
HE586515 (4)	AB742451 V4-A1	AY762603 V4-A2	FJ648514 V4-C2	HE586518 (2)	FJ592486 V4-C2	HE586505 V4-E	FJ592486 V4-C2	FJ648512 V4-C2	HQ287291 V4-G
HE586513 V4-C2	HQ317304 V4-C2	HE586513 V4-C2	FJ648512 V4-C2	JQ411021 (2)	FJ592339 V4-C2	HQ287928 V4-E	FJ592339 V4-C2	FJ946891 V4-C2	HQ287290 V4-G
HE586504 V4-C2	FN298927 V4-C3	HE586504 V4-C2	FJ946891 V4-C2	FJ592367 (9)	FJ592333 V4-C2	FN298928 V4-E	FJ592333 V4-C2	FJ592491 V4-C2	HQ287289 V4-G
JQ411021 (2)	FN298926 V4-C2	FR865679 (5)	FJ592491 V4-C2	FN597598 (12)	FN597599 (8)	FN597599 (8)	FN597599 (8)	FJ592486 V4-C2	HQ287288 V4-G
HQ317304 V4-C2	FJ648514 V4-C2	HQ317304 V4-C2	FJ592486 V4-C2	FR865679 (5)	JQ315652 V4-A2	HE586518 (2)	HE586518 (2)	FJ592339 V4-C2	HQ287287 V4-G
FN298927 V4-C3	FJ648512 V4-C2	FN298927 V4-C3	FJ592339 V4-C2	FN298927 V4-C3	HE586518 (2)	JQ411021 (2)	JQ411021 (2)	FJ592333 V4-C2	HQ287286 V4-G
FN298926 V4-C2	FJ946891 V4-C2	FN298926 V4-C2	FJ592333 V4-C2	AB260896 V4-C3	HE586517 V4-A2	FJ592367 (9)	FJ592367 (9)	AB260896 V4-C3	HQ287285 V4-G
FJ648514 V4-C2	AB488787 V4-A1	FJ648514 V4-C2	AB260896 V4-C3	AM743096 V4-C3	HE586511 V4-A2	FN597598 (12)	FN597598 (12)	AM743096 V4-C3	HQ287284 V4-G
FJ648512 V4-C2	FJ592491 V4-C2	FJ648512 V4-C2	AM743096 V4-C3	JQ315652 V4-A2	HE586510 V4-A2	FR865679 (5)	JQ315652 V4-A2	FN597599 (8)	HQ287282 V4-G
FJ946891 V4-C2	FJ592486 V4-C2	FJ946891 V4-C2	AY422078 (6)	HE586517 V4-A2	JQ411021 (2)	FN298927 V4-C3	HE586517 V4-A2	FR865679 (5)	HQ287281 V4-G
FJ592491 V4-C2	FJ592339 V4-C2	FJ592491 V4-C2	FN597599 (8)	HE586512 V4-E	GQ122371 V4-A2	AB260896 V4-C3	HE586511 V4-A2	FJ592367 (9)	HQ287280 V4-G
FJ592486 V4-C2	FJ592333 V4-C2	FJ592486 V4-C2	KC155323 (1)	HE586511 V4-A2	FJ592367 (9)	AM743096 V4-C3	HE586510 V4-A2	FN597598 (12)	HQ287278 V4-G
FJ592339 V4-C2	AB260896 V4-C3	FJ592339 V4-C2	HE586518 (2)	HE586510 V4-A2	AY762603 V4-A2	JQ315652 V4-A2	FR865679 (5)	JQ315652 V4-A2	HQ287277 V4-G
FJ592333 V4-C2	AM743096 V4-C3	FJ592333 V4-C2	AB742451 V4-A1	HE586505 V4-E	FN597598 (12)	HE586517 V4-A2	FN298927 V4-C3	HE586517 V4-A2	HQ287276 V4-G
AB260896 V4-C3	AY422078 (6)	AB260896 V4-C3	JQ411021 (2)	HQ287928 V4-E	AY422078 (6)	HE586511 V4-A2	GQ122371 V4-A2	HE586511 V4-A2	HQ287275 V4-G
AM743096 V4-C3	FN597599 (8)	AM743096 V4-C3	AB488787 V4-A1	FN298928 V4-E	AY494499 (13)	HE586510 V4-A2	AB260896 V4-C3	HE586510 V4-A2	HQ287274 V4-G
AY422078 (6)	HE586515 (4)	AY422078 (6)	FJ592367 (9)	GQ122371 V4-A2	KC155323 (1)	GQ122371 V4-A2	AM743096 V4-C3	GQ122371 V4-A2	HQ287273 V4-G
HE617184 (7)	FJ592367 (9)	FR850476 (10)	HE586514 (11)	AY762603 V4-A2	HE586512 V4-E	AY762603 V4-A2	AY762603 V4-A2	AY762603 V4-A2	HQ287272 V4-G
HE617183 (7)	HE586514 (11)	FN597599 (8)	FN597598 (12)	AY422078 (6)	HE586505 V4-E	KC155323 (1)	AY494499 (13)	JQ946088 (14)	HQ287271 V4-G
FN597599 (8)	HE617184 (7)	HE586518 (2)	HE586515 (4)	JQ946088 (14)	AB742451 V4-A1	AY422078 (6)	GQ487247 (17)		HQ287270 V4-G
KC155324 (7)	HE617183 (7)	HE586512 V4-E	HE586512 V4-E	KC155323 (1)	HQ287928 V4-E	JQ946088 (14)	GQ502290 (17)		FJ648513 V4-G
FJ592367 (9)	FN597598 (12)	HE586505 V4-E	HE586505 V4-E	AB742451 V4-A1	FN298928 V4-E	HQ287293 (16)	GQ502289 (17)		AM981206 V4-G

Table S6A: BLAST N search results of the different types of V4 region (SSU) among the *Coccomyxa* species (100% coverage; 97-100% identity).

C. subellipsoidea		*C. polymorpha*	*C. simplex*			*C. vinatzeri*	*C. galuniae*	*C. dispar*	*C. viridis*
**V4-A1**	**V4-A2**	**V4-B**	**V4-C1**	**V4-C2**	**V4-C3**	**V4-D**	**V4-E**	**V4-F**	**V4-G**
FR850476 (10)	KC155324 (7)	AB742451 V4-A1	HQ287928 V4-E	AB488787 V4-A1	AB488787 V4-A1	HQ287292 (16)	KC155323 (1)		EU127471 V4-G
HE586514 (11)	HE586516 (3)	JQ411021 (2)	FN298928 V4-E	HE586514 (11)	HE586514 (11)		FR865691 (17)		EU127470 V4-G
FN597598 (12)	HE586512 V4-E	HQ287928 V4-E	AY494499 (13)	HE586515 (4)	HE586515 (4)		AY422078 (6)		AM167525 V4-G
HE586512 V4-E	HE586505 V4-E	FN298928 V4-E	HE617184 (7)	HE617184 (7)	JQ946088 (14)		HE586514 (11)		AJ302939 V4-G
HE586505 V4-E	HQ287928 V4-E	AB488787 V4-A1	HE617183 (7)	HE617183 (7)	HE617184 (7)		JX169832 V4-G		HQ287283 (19)
HQ287928 V4-E	FN298928 V4-E	FJ592367 (9)	KC155324 (7)	KC155324 (7)	HE617183 (7)		HE586519 V4-G		AB721029 (20)
FN298928 V4-E	AY494499 (13)	FN597598 (12)	HE586516 (3)	HE586516 (3)	KC155324 (7)		HE586515 (4)		HQ287293 (16)
AY494499 (13)	JQ946088 (14)		FR850476 (10)	EU282454 (15)	HE586516 (3)		HE586509 V4-G		HQ287292 (16)
JQ946088 (14)	FR850476 (10)		JQ946088 (14)	FR850476 (10)	FR850476 (10)		HE586508 V4-G		EU127472 (21)
	EU282454 (15)		EU282454 (15)		EU282454 (15)		HE586507 V4-G		HE586512 V4-E
							HE586506 V4-G		HE586505 V4-E
							AB742451 V4-A1		HQ287928 V4-E
							JQ717057 V4-G		FN298928 V4-E
							JN573865 V4-G		
							FR865588 V4-G		
							HQ287296 V4-G		
							HQ287295 V4-G		
							HQ287294 V4-G		
							HQ287291 V4-G		
							HQ287290 V4-G		
							HQ287289 V4-G		
							HQ287288 V4-G		
							HQ287287 V4-G		
							HQ287286 V4-G		
							HQ287285 V4-G		
							HQ287284 V4-G		
							HQ287282 V4-G		
							HQ287281 V4-G		
							HQ287280 V4-G		
							HQ287278 V4-G		
							HQ287277 V4-G		
							HQ287276 V4-G		
							HQ287275 V4-G		
							HQ287274 V4-G		
							HQ287273 V4-G		

Table S6A: BLAST N search results of the different types of V4 region (SSU) among the *Coccomyxa* species (100% coverage; 97-100% identity).

C. subellipsoidea		*C. polymorpha*	*C. simplex*			*C. vinatzeri*	*C. galuniae*	*C. dispar*	*C. viridis*
**V4-A1**	**V4-A2**	**V4-B**	**V4-C1**	**V4-C2**	**V4-C3**	**V4-D**	**V4-E**	**V4-F**	**V4-G**
							HQ287272 V4-G		
							HQ287271 V4-G		
							HQ287270 V4-G		
							FJ648513 V4-G		
							AM981206 V4-G		
							AB488787 V4-A1		
							FJ592475 (18)		
							EU127471 V4-G		
							EU127470 V4-G		
							AM167525 V4-G		
							AJ302939 V4-G		
							JQ946088 (14)		
							EU282454 (15)		
100 %									
99 %									
98 %									
97 %									

Table S6B: BLAST N search results of the different types of V9 region (SSU) among the *Coccomyxa* species (100% coverage; 97-100% identity).

C. sub./C.sim.	*C. polymorpha*	*C. vinatzeri*	*C. galuniae*	*C. dispar*	*C. viridis*	
**V9-A**	**V9-B**	**V9-C**	**V9-D**	**V9-E**	**V9-F1**	**V9-F2**
FN597599 V9-A	HE617184 V9-B	FN597599 V9-A	HE586541 V9-D	FJ553991 V9-E	JX169832 V9-F1	AJ302939 V9-F2
FN597598 V9-A	HE617183 V9-B	FN597598 V9-A	HE586540 V9-D		HE586523 V9-F1	JX169832 V9-F1
JQ315652 V9-A	FR850476 V9-B	JQ315652 V9-A	HE586521 V9-D		HE586522 V9-F1	HE586523 V9-F1
HE586545 V9-A	JX869405 V9-B	HE586545 V9-A	HE586520 V9-D		HE586519 V9-F1	HE586522 V9-F1
HE586524 V9-A	HE586516 V9-B	HE586541 V9-D	HE586512 V9-D		HE586508 V9-F1	HE586519 V9-F1
HE586518 V9-A	HE586515 V9-B	HE586540 V9-D	FN298928 V9-D		HE586507 V9-F1	HE586508 V9-F1
HE586517 V9-A	HE586514 V9-B	HE586524 V9-A	HE586505 (6)		FR865588 V9-F1	HE586507 V9-F1
HE586504 V9-A		HE586521 V9-D			FJ648513 V9-F1	FR865588 V9-F1
FR865679 V9-A		HE586520 V9-D			AM981206 V9-F1	FJ648513 V9-F1
HQ317304 V9-A		HE586518 V9-A			EU127471 V9-F1	AM981206 V9-F1
FN298927 V9-A		HE586517 V9-A			EU127470 V9-F1	EU127471 V9-F1
FN298926 V9-A		HE586512 V9-D			AM167525 V9-F1	EU127470 V9-F1
FJ648514 V9-A		HE586504 V9-A			HE586506 (7)	AM167525 V9-F1
FJ946891 V9-A		FR865679 V9-A			HE586536 (8)	HE586506 (7)
GQ122371 V9-A		HQ317304 V9-A			AJ302939 V9-F2	HE586538 (9)
AB488788 V9-A		FN298928 V9-D			HE586538 (9)	HE586536 (8)
AB260896 V9-A		FN298927 V9-A			HE586509 (9)	
AY762603 V9-A		FN298926 V9-A				
HE586511 (1)		FJ648514 V9-A				
HE586513 (2)		FJ946891 V9-A				
HE586510 (3)		GQ122371 V9-A				
FJ648512 (4)		AB488788 V9-A				100 %
AB488795 (5)		AB260896 V9-A				99 %
		AY762603 V9-A				98 %
		HE586511 (1)				97 %

Table S6C: BLAST N search results of the different types of ITS-2 among the *Coccomyxa* species (100% coverage; 97-100% identity).

C. subellipsoidea				*C. polymorpha*	*C. simplex*					
**BC-1a**	**BC-1b**	**BC-1c**		**BC-2**	**BC-3a**	**BC-3b**			**BC-3c**	
**ITS2-A1+A2**	**ITS2-A3**	**ITS2-A4**	**ITS2-A5**	**ITS2-B**	**ITS2-C1**	**ITS2-C2**	**ITS2-C3**	**ITS2-C4**	**ITS2-C5+C6**	**ITS2-C7**
HE586527 A1	AY293948 (3)	AY328523 A3	AY328523 A3	HE586514 B	HE586524 C1	FN597598 (19)	FN298926 C4	FN298926 C4	FN597599 C5	FN298927 C7
HE586517 (1)	AY293942 (3)	AY293947 A3	AY293947 A3		FN597599 C5	HE586504 (20)	HE586504 (20)	HE586504 (20)	HE586551 C5	AB260896 C7
AY293932 (1)	HE586517 (1)	AY293945 (8)	AY293945 (8)		HE586551 C5	FN298926 C4	AY328524 (21)	AY328524 (21)	HE586545 C5	
HE586553 (2)	AY293943 (5)	AY293944 (8)	AY293944 (8)		HE586545 C5	AY328524 (21)			HE586513 C5	
HE586552 (2)	AY293938 (5)	AY293941 (8)	AY293941 (8)		HE586513 C5				AY293967 C5	
AY293948 (3)	AY293936 (5)	AY293937 (8)	AY293937 (8)		AY293967 C5				AY293966 C5	
AY293946 (4)	AY293932 (1)	AY293933 (8)	AY293933 (8)		AY293966 C5				AY328522 C5	
AY293942 (3)	HE586557 (6)	AY293948 (3)	AY293948 (3)		AY328522 C5				AY333648 C5	
AY293939 (2)	AY333650 (7)	AY293942 (3)	AY293942 (3)		AY333648 C5				AY333647 C5	
		HE586511 (9)	HE586511 (9)		AY333647 C5				AY333649 C5	
		HE586553 (2)	HE586553 (2)		AY333649 C5				AY293964 (17)	
		HE586552 (2)	HE586552 (2)		AY293964 (17)				AY293965 (18)	
		HE586517 (1)	HE586517 (1)		AY293965 (18)				HE586524 B1	
		AY293943 (5)	AY293943 (5)							
		AY293939 (2)	AY293939 (2)							
		AY293938 (5)	AY293938 (5)							
		AY293936 (5)	AY293936 (5)							
		AY293932 (1)	AY293932 (1)							
		HE586554 (10)	HE586554 (10)							
		HE586544 (11)	HE586544 (11)							
		HE586510 (10)	HE586510 (10)							
		HE586556 (12)	HE586556 (12)							
		HE586555 (12)	HE586555 (12)							
		HE586543 (13)	HE586543 (13)							
		AY293946 (4)	AY293946 (4)							
100 %		AY293935 (14)	AY293935 (14)							
99 %		AY293934 (15)	AY293934 (15)							
98 %		AY333650 (7)	AY333650 (7)							
97 %		AY293940 (16)	AY293940 (16)							

Table S6C: BLAST N search results of the different types of ITS-2 among the *Coccomyxa* species (100% coverage; 97-100% identity).

C. vinatzeri	*C. galuniae*	*C. dispar*	*C. viridis*				
**BC-4**	**BC-5**	**BC-6**	**BC-7a**			**BC-7b**	
**ITS2-D**	**ITS2-E**	**ITS2-F**	**ITS2-G1**	**ITS2-G2**	**ITS2-G3**	**ITS2-G4**	**ITS2-G5**
-	JQ082333 E	-	HE586522 G2	HE586522 G2	HE586538 G3	-	-
	FN298928 E		DQ660909 G2	DQ660909 G2	HE586537 G3		
			HE586523 (22)	HE586523 (22)	HE586535 G3		
					HE586534 (23)		
					HE586531 (24)		
					HE586533 (25)		
					HE586532 (26)		
					HE586539 (27)		
