# Supplementary material for: Evaluating the Species Boundaries of Green Microalgae (Coccomyxa, Trebouxiophyceae, Chlorophyta) Using Integrative Taxonomy and DNA Barcoding with Further Implications for the Species Identification in Environmental Samples
Source: PLoS One. 2015 Jun 16;10(6):e0127838. doi: 10.1371/journal.pone.0127838 (PMC4469705; doi:10.1371/journal.pone.0127838)
Supplement: S7 Table — (B) Meta data of the GenBank entries found by the BLAST search of the different barcode regions and its evaluation. The ITS-2 of the accession numbers highlighted in orange represented new lineages and the secondary structure is shown in S2 Fig. The entries under the column ‘culture/environment’ (C/E) in blue represent multiple copies of ITS-2. The uncertain species identification of some entries are marked with an asterisk. The entries with a number in brackets under the columns ‘V4’ ‘V9’ and ‘ITS-2’ are evaluated using the secondary structures of these regions. The entries in pink represent taxa, which do not belong to Coccomyxa. Details are written in the text. (PDF) [file pone.0127838.s014.pdf]

Table S7A: ITS-2 Barcode and population identifier

	Barcode	ITS-2	V4	V9
	ID	POP-ID	POP-ID	POP-ID
NIES 2166	1a	A1	A1	A
NIES 2353	1a	A1	A1	A
CCAP 812/3	1a	A1	A1	A
NIES 2252	1a	A2	A1	A
CAUP H5105	1b	A3	A2	A
Wien C20	1c	A4	A2	A
SAG 216-7	1c	A4	A2	A
SAG 216-13	1c	A4	A2	A
SAG 69.80	1c	A5	A2	A
CAUP H5101	2	B	B	B
SAG 216-3b	3a	C1	C1	A
CAUP H5107	3a	C1	C1	A
SAG 216-2	3b	C2	C2	A
SAG 216-3c	3b	C3	C2	A
SAG 216-8	3b	C4	C2	A
SAG 216-9a	3b	C4	C2	A
SAG 216-5	3c	C5	C2	A
SAG 216-10	3c	C5	C2	A
SAG 216-12	3c	C5	C2	A
CCAP 216/15	3c	C5	C2	A
SAG 216-11a	3c	C6	C2	A
SAG 216-11b	3c	C6	C2	A
SAG 216-6	3c	C6	C2	A
CCAP 216/24	3c	C7	C3	A
CCAP 812/2A	3c	C7	C3	A
CCAP 812/2B	3c	C7	C3	A
ASIB V16	4	D	D	C
CCAP 211/97	5	E	E	D
CCAP 812/5	5	E	E	D
SAG 2253	5	E	E	D
SAG 2254	5	E	E	D
SAG 49.84	6	F	F	E
SAG 216-1	7a	G1	G	F1
Wien C19	7a	G2	G	F1
SAG 216-4	7a	G3	G	F1
SAG 216-14	7a	G3	G	F1
SAG 2104	7a	G3	G	F1
SAG 2040	7b	G4	G	F2
SAG 2127	7b	G4	G	F2
SAG 2325	7b	G4	G	F2
CAUP H5103	7b	G5	G	F2

Table S7B: Meta data of the GenBank entries found by the BLAST search of the different barcode regions and its evaluation.

	origin	life style	climate	C/E	length		V4	V9	ITS-2	BC	identification
HE586505	Europe	terrestrial	moderate continental	CAUP H103	1744	SSU ++	V4-E	V9-D (6)	-	-	*C. galuniae*
FN298928	Europe	aquatic	moderate continental	CCAP 211/97	2452	SSU/ITS +++	V4-E	V9-D	ITS2-E	BC-5	*C. galuniae*
HE586512	Europe	terrestrial	moderate continental	C* (C15)	1753	SSU +	V4-E	-	-	-	*C. galuniae*
HQ287928	Europe	aquatic	moderate continental	C* (KR 1988/12)	1609	SSU +	V4-E	-	-	-	*C. galuniae*
JQ082333	Europe	aquatic	moderate continental	C*(KR 1981/341)	758	ITS +	-	-	ITS2-E	BC-5	*C. galuniae*
HE586514	Asia	terrestrial	tropical	C* (T2)	2455	SSU/ITS +++	V4-B (11)	V9-B	ITS2-B	BC-2	*C. polymorpha*
JX869405	Europe	aquatic	moderate continental	E	938	SSU -+	-	V9-B	-	-	*C. polymorpha* ?
HE586513	Europe	symbiotic	moderate continental	C* (E4)	2443	SSU/ITS +++	V4-C2	V9-A (2)	ITS2-C5	BC-3c	*C. simplex*
HE586504	Europe	aquatic	moderate continental	CAUP H102	2412	SSU/ITS +++	V4-C2	V9-A	ITS2-C4 (20)	BC-3b	*C. simplex*
FN298926	Europe	aquatic	moderate continental	SAG 216-9a	2414	SSU/ITS +++	V4-C2	V9-A	ITS2-C4	BC-3b	*C. simplex*
HQ317304	Europe	terrestrial	moderate continental	UTEX 273	1793	SSU ++	V4-C2	V9-A	-	-	*C. simplex*
FJ648514	Europe	aquatic	moderate continental	UTEX 274	1760	SSU ++	V4-C2	V9-A	-	-	*C. simplex*
FJ648512	Europe	aquatic	moderate continental	SAG 216-2	1762	SSU ++	V4-C2	V9-A (4)	-	-	*C. simplex*
FN597598*	Europe	aquatic	moderate continental	SAG 216-2	3103	SSU/ITS +++	V4-C2 (12)	V9-A	ITS2-C2 (19)	-	*C. simplex*
FJ946891	Antarctic	terrestrial	cold desert	VPL5-6	1769	SSU ++	V4-C2	V9-A	-	-	*C. simplex*
FN298927	Europe	aquatic	atlantic	CCAP 216/24	2434	SSU/ITS +++	V4-C3	V9-A	ITS2-C7	BC-3c	*C. simplex*
AB260896	Europe	aquatic	atlantic	E	2433	SSU/ITS +++	V4-C3	V9-A	ITS2-C7	BC-3c	*C. simplex*
FJ592486	S. America	terrestrial	cold desert	E	1083	SSU +	V4-C2	-	-	-	*C. simplex*
FJ592339	S. America	terrestrial	cold desert	E	1083	SSU +	V4-C2	-	-	-	*C. simplex*
FJ592333	S. America	terrestrial	cold desert	E	1083	SSU +	V4-C2	-	-	-	*C. simplex*
AM743096	Europe	aquatic	mediterran	C* (L821)**	1601	SSU +	V4-C3	-	-	-	*C. simplex*
HE586518	N. America	terrestrial	moderate continental	C* (GSE4G)	2419	SSU/ITS ++-	V4-C2 (2)	V9-A	ITS2-C8*	BC-3c	*C. simplex*
FN597599	Europe	symbiotic	moderate continental	SAG 216-5	3103	SSU/ITS +++	V4-C2 (8)	V9-A	ITS2-C5	BC-3c	*C. simplex*
AY328522	Europe	symbiotic	moderate continental	SAG 216-5	725	ITS +	-	-	ITS2-C5	BC-3c	*C. simplex*
HE586524	Europe	aquatic	moderate continental	UTEX 2460	924	SSU/ITS -++	-	V9-A	ITS2-C1	BC-3a	*C. simplex*
AY422078	Europe	aquatic	moderate continental	UTEX 2460	1698	SSU +	V4-C1 (6)	-	-	-	*C. simplex*
HE586545	Europe	symbiotic	mediterran	C* (E5)	973	SSU/ITS -++	-	V9-A	ITS2-C5	BC-3c	*C. simplex*
HE586551	Asia	terrestrial	tropical	C* (T5)	632	ITS +	-	-	ITS2-C5	BC-3c	*C. simplex*
AY293967	Europe	symbiotic	moderate continental	UTEX 277	651	ITS +	-	-	ITS2-C5	BC-3c	*C. simplex*
AY293966	Europe	symbiotic	moderate continental	UTEX 275	651	ITS +	-	-	ITS2-C5	BC-3c	*C. simplex*
AY333648	Europe	symbiotic	continental	E	612	ITS +	-	-	ITS2-C5	BC-3c	*C. simplex*
AY333647	Europe	symbiotic	continental	E	612	ITS +	-	-	ITS2-C5	BC-3c	*C. simplex*
AY333649	Europe	symbiotic	continental	E	612	ITS +	-	-	ITS2-C5	BC-3c	*C. simplex*
AY293964	Europe	symbiotic	continental	UTEX 271	610	ITS +	-	-	ITS2-C6 (17)	BC-3c	*C. simplex*
AY293965	Europe	symbiotic	moderate continental	UTEX 276	650	ITS +	-	-	ITS2-C6 (18)	BC-3c	*C. simplex*
AY328524	Europe	terrestrial	moderate continental	SAG 216-8	696	ITS +	-	-	ITS2-C4 (21)	BC-3b	*C. simplex*
FJ592491	S. America	terrestrial	cold desert	E	1083	SSU +	V4-C2	-	-	-	*C. simplex*
JQ411021	Asia	unknown	unknown	C* (GTD2A1)	1246	SSU +	V4-C2 (2)	-	-	-	*C. simplex* *
FJ592367	S. America	terrestrial	cold desert	E	1083	SSU +	V4-C2 (9)	-	-	-	*C. simplex* *
JQ946088	Asia	unknown	unknown	C* (XDL-2012)	1677	SSU +	V4-C2 (14)	-	-	-	*C. simplex* *
EU282454	Asia	terrestrial	cold desert	C* (294-GA206)	1704	SSU +	V4-C2 (15)	-	-	-	*C. simplex* *
AY494499	Europe	terrestrial	continental	E	507	SSU +	V4-A2/C2 (13)	-	-	-	*C. simplex /C. subellipsoidea* ? **
FJ553991	N. America	terrestrial	cold desert	E	1003	SSU/ITS -+-	-	V9-E	ITS2-J*	BC-8*	*C. sp.* 1*
HE586516	Asia	terrestrial	tropical	C* (T4)	3440	SSU/ITS ++-	V4-B (3)	V9-B	ITS2-K1*	BC-9a*	*C. sp.* 2*
FR850476	Europe	aquatic	moderate continental	CCAP 216/25	4065	SSU/ITS ++-	V4-B (10)	V9-B	ITS2-K2*	BC-9b*	*C. sp.* 2*
HE586515	Asia	terrestrial	tropical	C* (T3)	2802	SSU/ITS ++-	V4-B (4)	V9-B	ITS2-L1*	BC-10*	*C. sp.* 3*
HE586550	Asia	terrestrial	tropical	C* (T1)	656	ITS-	-	-	ITS2-L1*	BC-10*	*C. sp.* 3*

Table S7B: Meta data of the GenBank entries found by the BLAST search of the different barcode regions and its evaluation.

	origin	life style	climate	C/E	length		V4	V9	ITS-2	BC	identification
HE617183	Europe	aquatic	moderate continental	C* (ACCV1)	2925	SSU/ITS ++-	V4-B (7)	V9-B	ITS2-L2*	BC-10*	*C. sp.* 3*
HE617184	Europe	aquatic	moderate continental	E	1797	SSU ++	V4-B (7)	V9-B	-	-	*C. sp.* 3* ?
KC155324	Europe	aquatic	mediterran	C* (AH4)	1526	SSU +	V4-B (7)	-	-	-	*C. sp.* 3* ?
KC155323	Europe	aquatic	mediterran	C* (AC1)	1516	SSU +	V4-B (1)	-	-	-	*C. sp.* 3* ?
HE586506	N. America	terrestrial	moderate continental	UTEX SNO83	2504	SSU/ITS ++-	V4-G	V9-F1 (7)	ITS2-M*	BC-11*	*C. sp.* 4*
HE586508	Europe	terrestrial	moderate continental	C* (C4)	2482	SSU/ITS ++-	V4-G	V9-F1	ITS2-N*	BC-12*	*C. sp.* 5*
HE586549	Europe	terrestrial	mediterran	C* (S2)	735	ITS-	-		ITS2-N*	BC-12*	*C. sp.* 5*
HE586548	Europe	terrestrial	mediterran	C* (S2)	734	ITS-	-	-	ITS2-N*	BC-12*	*C. sp.* 5*
HE586530	Europe	terrestrial	moderate continental	C* (C3)	739	ITS-	-	-	ITS2-N*	BC-12*	*C. sp.* 5*
HE586529	Europe	terrestrial	moderate continental	C* (C3)	738	ITS-	-	-	ITS2-N*	BC-12*	*C. sp.* 5*
HE586528	Europe	terrestrial	moderate continental	C* (C2)	731	ITS-	-	-	ITS2-N*	BC-12*	*C. sp.* 5*
HE586547	Europe	terrestrial	mediterran	C* (S1)	734	ITS-	-	-	ITS2-N*	BC-12*	*C. sp.* 5*
HE586546	Europe	terrestrial	mediterran	C* (S1)	733	ITS-	-	-	ITS2-N*	BC-12*	*C. sp.* 5*
AY333646	Europe	symbiotic	moderate continental	CCALA 306	690	ITS-	-	-	ITS2-N*	BC-12*	*C. sp.* 5*
AB742451	Asia	aquatic	tropical	C* (KGU-D001)	1730	SSU +	V4-A1	-	-	-	*C. subellipsoidea*
JQ315652	Asia	aquatic	tropical	C* (KMMCC 15)	1676	SSU ++	V4-A2	V9-A	-	-	*C. subellipsoidea*
HE586517	Europe	terrestrial	moderate continental	C* (U2)	2822	SSU/ITS +++	V4-A2	V9-A	ITS2-A4 (1)	BC-1c	*C. subellipsoidea*
HE586511	Europe	terrestrial	moderate continental	C*(C14)	2849	SSU/ITS +++	V4-A2	V9-A (1)	ITS2-A4 (9)	BC-1c	*C. subellipsoidea*
HE586510	Europe	terrestrial	moderate continental	C*(C13)	2807	SSU/ITS +++	V4-A2	V9-A (3)	ITS2-A4 (10)	BC-1c/d?*	*C. subellipsoidea*
GQ122371	Asia	aquatic	tropical	C* (KMMCC 10)	1675	SSU ++	V4-A2	V9-A	-	-	*C. subellipsoidea*
AY762603	Europe	symbiotic	moderate continental	SAG 69.80	2579	SSU ++	V4-A2	V9-A	-	-	*C. subellipsoidea*
FR865679	unknown	unknown	unknown	CCAP 211/60	1978	SSU ++	V4-A2 (5)	V9-A	-	-	*C. subellipsoidea*
AB488787	unknown	unknown	unknown	NIES 2252	1239	SSU +	V4-A1	-	-	-	*C. subellipsoidea*
HE586527	Europe	terrestrial	moderate continental	C*(C1)	649	ITS +	-	-	ITS2-A1	BC-1a	*C. subellipsoidea*
AY328523	Europe	symbiotic	moderate continental	SAG 216-13	702	ITS +	-	-	ITS2-A3	BC-1b	*C. subellipsoidea*
AY293947	N. America	symbiotic	cold desert	E	624	ITS +	-	-	ITS2-A3	BC-1b	*C. subellipsoidea*
AY293932	N. America	symbiotic	continental	E	625	ITS +	-	-	ITS2-A4 (1)	BC-1c	*C. subellipsoidea*
HE586553	Europe	terrestrial	moderate continental	C* (U1)	688	ITS +	-	-	ITS2-A4 (2)	BC-1c	*C. subellipsoidea*
HE586552	Europe	terrestrial	moderate continental	C* (U1)	687	ITS +	-	-	ITS2-A4 (2)	BC-1c	*C. subellipsoidea*
AY293939	N. America	symbiotic	cold desert	E	687	ITS +	-	-	ITS2-A4 (2)	BC-1c	*C. subellipsoidea*
AY293948	N. America	symbiotic	continental	E	624	ITS +	-	-	ITS2-A4 (3)	BC-1c	*C. subellipsoidea*
AY293942	Europe	symbiotic	cold desert	E	624	ITS +	-	-	ITS2-A4 (3)	BC-1c	*C. subellipsoidea*
AY293946	N. America	symbiotic	continental	E	626	ITS +	-	-	ITS2-A4 (4)	BC-1d*	*C. subellipsoidea*
AY293943	N. America	symbiotic	cold desert	E	625	ITS +	-	-	ITS2-A4 (5)	BC-1d*	*C. subellipsoidea*
AY293938	N. America	symbiotic	cold desert	E	625	ITS +	-	-	ITS2-A4 (5)	BC-1d*	*C. subellipsoidea*
AY293936	N. America	symbiotic	continental	E	625	ITS +	-	-	ITS2-A4 (5)	BC-1d*	*C. subellipsoidea*
AY293935	N. America	symbiotic	cold desert	E	625	ITS +	-	-	ITS2-A4 (14)	BC-1d*	*C. subellipsoidea*
AY293934	N. America	symbiotic	cold desert	E	625	ITS +	-	-	ITS2-A4 (15)	BC-1d*	*C. subellipsoidea*
AY293940	N. America	symbiotic	cold desert	E	625	ITS +	-	-	ITS2-A4 (16)	BC-1d*	*C. subellipsoidea*
HE586557	Europe	terrestrial	moderate continental	C* (U5)	659	ITS +	-	-	ITS2-A3 (6)	BC-1c	*C. subellipsoidea*
AY333650	N. America	symbiotic	continental	E	597	ITS +	-	-	ITS2-A4 (7)	BC-1c	*C. subellipsoidea*
AY293945	N. America	symbiotic	continental	E	624	ITS +	-	-	ITS2-A4 (8)	BC-1c	*C. subellipsoidea*
AY293944	N. America	symbiotic	continental	E	624	ITS +	-	-	ITS2-A4 (8)	BC-1c	*C. subellipsoidea*
AY293941	N. America	symbiotic	cold desert	E	623	ITS +	-	-	ITS2-A4 (8)	BC-1c	*C. subellipsoidea*
AY293937	N. America	symbiotic	cold desert	E	624	ITS +	-	-	ITS2-A4 (8)	BC-1c	*C. subellipsoidea*
AY293933	N. America	symbiotic	cold desert	E	624	ITS +	-	-	ITS2-A4 (8)	BC-1c	*C. subellipsoidea*
HE586554	Europe	terrestrial	moderate continental	C* (U3)	847	ITS +	-	-	ITS2-A4 (10)	BC-1c/d?*	*C. subellipsoidea*

Table S7B: Meta data of the GenBank entries found by the BLAST search of the different barcode regions and its evaluation.

	origin	life style	climate	C/E	length		V4	V9	ITS-2	BC	identification
HE586544	Europe	symbiotic	mediterran	C* (E3)	662	ITS +	-	-	ITS2-A4 (11)	BC-1c/d?*	*C. subellipsoidea*
HE586556	Europe	terrestrial	moderate continental	C* (U4)	618	ITS +	-	-	ITS2-A4 (12)	BC-1c	*C. subellipsoidea*
HE586555	Europe	terrestrial	moderate continental	C* (U4)	618	ITS +	-	-	ITS2-A4 (12)	BC-1c	*C. subellipsoidea*
HE586543	Europe	symbiotic	mediterran	C* (E2)	611	ITS +	-	-	ITS2-A4 (13)	BC-1c/d?*	*C. subellipsoidea*
AB488788	unknown	unknown	unknown	NIES 2252	1224	SSU -+	-	V9-A	-	-	*C. subellipsoidea* *
AB488795	Antarctic	terrestrial	cold desert	NIES 2353	1235	SSU -+	-	V9-A (5)	-	-	*C. subellipsoidea* *
JX169832	Europe	terrestrial	moderate continental	C* (GOGrp_K07)	1763	SSU ++	V4-G	V9-F1	-	-	*C. viridis*
HE586519	N. America	terrestrial	moderate continental	C* (CR-2)	2550	SSU/ITS ++-	V4-G	V9-F1	ITS2-G6*	BC-7a	*C. viridis*
FR865588	unknown	aquatic	unknown	CCAP 11/47	1826	SSU ++	V4-G	V9-F1	-	-	*C. viridis*
HQ287283	Europe	symbiotic	continental	E	1332	SSU +	V4-G (19)	-	-	-	*C. viridis*
HE586507	N. America	terrestrial	continental	UTEX SNO113	1779	SSU ++	V4-G	V9-F1	-	-	*C. viridis*
HE586509	Europe	terrestrial	moderate continental	C* (C10)	1762	SSU ++	V4-G	V9-F1 (9)	-	-	*C. viridis*
JQ717057	Asia	parasitic	continental	E (par)	1521	SSU +	V4-G	-	-	-	*C. viridis*
JN573865	N. America	symbiotic	moderate continental	E	1328	SSU +	V4-G	-	-	-	*C. viridis*
HQ287296	N. America	symbiotic	continental	E	1319	SSU +	V4-G	-	-	-	*C. viridis*
HQ287295	Europe	symbiotic	continental	E	1306	SSU +	V4-G	-	-	-	*C. viridis*
HQ287294	Europe	symbiotic	continental	E	525	SSU +	V4-G	-	-	-	*C. viridis*
HQ287291	Europe	symbiotic	continental	E	1304	SSU +	V4-G	-	-	-	*C. viridis*
HQ287290	Europe	symbiotic	continental	E	1317	SSU +	V4-G	-	-	-	*C. viridis*
HQ287289	Europe	symbiotic	continental	E	1317	SSU +	V4-G	-	-	-	*C. viridis*
HQ287288	Europe	symbiotic	continental	E	1314	SSU +	V4-G	-	-	-	*C. viridis*
HQ287287	Europe	symbiotic	continental	E	1062	SSU +	V4-G	-	-	-	*C. viridis*
HQ287286	Europe	symbiotic	continental	E	1308	SSU +	V4-G	-	-	-	*C. viridis*
HQ287285	Europe	symbiotic	continental	E	1336	SSU +	V4-G	-	-	-	*C. viridis*
HQ287284	Europe	symbiotic	continental	E	1312	SSU +	V4-G	-	-	-	*C. viridis*
HQ287282	Europe	symbiotic	continental	E	1100	SSU +	V4-G	-	-	-	*C. viridis*
HQ287281	Europe	symbiotic	continental	E	1333	SSU +	V4-G	-	-	-	*C. viridis*
HQ287280	Europe	symbiotic	continental	E	1324	SSU +	V4-G	-	-	-	*C. viridis*
HQ287278	Europe	symbiotic	continental	E	1335	SSU +	V4-G	-	-	-	*C. viridis*
HQ287277	Europe	symbiotic	continental	E	1043	SSU +	V4-G	-	-	-	*C. viridis*
HQ287276	Europe	symbiotic	continental	E	1337	SSU +	V4-G	-	-	-	*C. viridis*
HQ287275	Europe	symbiotic	continental	E	1305	SSU +	V4-G	-	-	-	*C. viridis*
HQ287274	Europe	symbiotic	continental	E	960	SSU +	V4-G	-	-	-	*C. viridis*
HQ287273	Europe	symbiotic	continental	E	1318	SSU +	V4-G	-	-	-	*C. viridis*
HQ287272	Europe	symbiotic	continental	E	1288	SSU +	V4-G	-	-	-	*C. viridis*
HQ287271	Europe	symbiotic	continental	E	1338	SSU +	V4-G	-	-	-	*C. viridis*
HQ287270	Europe	symbiotic	continental	E	847	SSU +	V4-G	-	-	-	*C. viridis*
FJ648513	Europe	symbiotic	moderate continental	SAG 216-4	1761	SSU ++	V4-G	V9-F1	-	-	*C. viridis*
AM981206	N. America	aquatic	continental	CPCC 508	1797	SSU ++	V4-G	V9-F1	-	-	*C. viridis*
EU127471	Europe	parasitic	continental	E (par)	1778	SSU ++	V4-G	V9-F1	-	-	*C. viridis*
EU127470	Europe	parasitic	continental	E (par)	1764	SSU ++	V4-G	V9-F1	-	-	*C. viridis*
AM167525	Europe	symbiotic	moderate continental	CCALA 306	1792	SSU ++	V4-G	V9-F1	-	-	*C. viridis*
AJ302939	Europe	symbiotic	moderate continental	SAG 2325	1798	SSU ++	V4-G	V9-F1	-	-	*C. viridis*
HQ287293	Europe	symbiotic	continental	E	1325	SSU +	V4-G (16)	-	-	-	*C. viridis*
HQ287292	Europe	symbiotic	continental	E	1315	SSU +	V4-G (16)	-	-	-	*C. viridis*
AB721029	Asia	aquatic	tropical	E	1682	SSU +	V4-G (20)	-	-	-	*C. viridis*
EU127472	Europe	aquatic	continental	E (par)	1450	SSU +	V4-G (21)	-	-	-	*C. viridis*

Table S7B: Meta data of the GenBank entries found by the BLAST search of the different barcode regions and its evaluation.

	origin	life style	climate	C/E	length		V4	V9	ITS-2	BC	identification
HE586523	N. America	terrestrial	continental	UTEX SNO113	1024	SSU/ITS -++	-	V9-F1	ITS2-G1 (22)	BC-7a	*C. viridis*
HE586522	N. America	terrestrial	continental	UTEX SNO113	1028	SSU/ITS -++	-	V9-F1	ITS2-G2	BC-7a	*C. viridis*
DQ660909	unknown	symbiotic	unknown	E	797	ITS +	-	-	ITS2-G2	BC-7a	*C. viridis*
HE586536	Europe	terrestrial	moderate continental	C* (C9)	898	SSU/ITS -+-	-	V9-F1 (8)	ITS2-G7*	BC-7a	*C. viridis*
HE586538	Europe	terrestrial	moderate continental	C* (C10)	897	SSU/ITS -++	-	V9-F1 (9)	ITS2-G3	BC-7a	*C. viridis*
HE586537	Europe	terrestrial	moderate continental	C* (C10)	762	ITS +	-	-	ITS2-G3	BC-7a	*C. viridis*
HE586535	Europe	terrestrial	moderate continental	C* (C8)	762	ITS +	-	-	ITS2-G3	BC-7a	*C. viridis*
HE586534	Europe	terrestrial	moderate continental	C* (C8)	764	ITS +	-	-	ITS2-G3 (23)	BC-7a	*C. viridis*
HE586531	Europe	terrestrial	moderate continental	C* (C5)	893	ITS +	-	?	ITS2-G3 (24)	BC-7a	*C. viridis*
HE586533	Europe	terrestrial	moderate continental	C* (C7)	903	ITS +	-	?	ITS2-G3 (25)	BC-7a	*C. viridis*
HE586532	Europe	terrestrial	moderate continental	C* (C6)	855	ITS +	-	?	ITS2-G3 (26)	BC-7a*	*C. viridis*
HE586539	Europe	terrestrial	moderate continental	C* (C11)	882	ITS +	-	?	ITS2-G3 (27)	BC-7a*	*C. viridis*
GQ487247	Europe	aquatic	moderate continental	CCALA 333	1777		17				*Xerochlorella*
GQ502290	Antarctic	terrestrial	cold desert	UTEX SNO65	2476		17				*Xerochlorella*
GQ502289	Antarctic	terrestrial	cold desert	CCAP 222/3	2476		17				*Xerochlorella*
FR865691	Antarctic	terrestrial	cold desert	CCAP 222/3	2025		17				*Xerochlorella*
FJ592475	S. America	terrestrial	cold desert	E	1083		18				*Xerochlorella*

Table S7B: Meta data of the GenBank entries found by the BLAST search of the different barcode regions and its evaluation.

	region: no. base differences, evaluation	comment
**HE586505**	**V9-D (6):** 3 bp, errors	
**FN298928**	-	
**HE586512**	-	
**HQ287928**	-	
**JQ082333**	-	
**HE586514**	**V4-B (11):** 1 bp, ambiguity	
**JX869405**	-	
**HE586513**	**V9-A (2):** 2 bp, ambiguities	
**HE586504**	**ITS2-C4 (20):** 1 bp (Helix II), ambiguity	
**FN298926**	-	
**HQ317304**	-	
**FJ648514**	-	
**FJ648512**	**V9-A (4):** 2 bp, ambiguity+error	
**FN597598***	**V4-C2 (12):** 2 bp; **ITS2-C2 (19):** 4 bp (Helices II+III), errors	**sequence with many errors and ITS-2 incomplete**
**FJ946891**	-	
**FN298927**	-	
**AB260896**	-	
**FJ592486**	-	
**FJ592339**	-	
**FJ592333**	-	
**AM743096**	-	
**HE586518**	**V4-C2 (2):** 1 bp	**new variant of ITS2 (ITS2-C8*)**
**FN597599**	**V4-C2 (8):** 1 bp, error	
**AY328522**	-	
**HE586524**	-	
**AY422078**	**V4-C1 (6):** 3 bp, errors	
**HE586545**	-	
**HE586551**	-	
**AY293967**	-	
**AY293966**	-	
**AY333648**	-	
**AY333647**	-	
**AY333649**	-	
**AY293964**	**ITS2-C6 (17):** 3 bp (Helix III), ambiguities	
**AY293965**	**ITS2-C6 (18):** 1 bp (Helix III), error	
**AY328524**	**ITS2-C4 (21):** 1 bp (Helix II)	
**FJ592491**	-	
**JQ411021**	**V4-C2 (2):** 1 bp	*** BLAST of complete entry**
**FJ592367**	**V4-C2 (9):** 1 bp, HCBC	*** BLAST of complete entry**
**JQ946088**	**V4-C2 (14):** 3 bp, missing base+errors	*** BLAST of complete entry**
**EU282454**	**V4-C2 (15):** 4 bp, errors	*** BLAST of complete entry**
**AY494499**	**V4-A2/C2 (13):** 2 bp, missing base+error	**** sequence with errors, too short for exact identification**
**FJ553991**	-	**new ITS2-J***
**HE586516**	**V4-B (3):** 4 bp; **ITS2-K1:** 1 bp (Helix III), ambiguity, error	
**FR850476**	**V4-B (10):** 4 bp, one error	
**HE586515**	**V4-B (4):** 1 bp	
**HE586550**	-	

Table S7B: Meta data of the GenBank entries found by the BLAST search of the different barcode regions and its evaluation.

	region: no. base differences, evaluation	comment
**HE617183**	**V4-B (7):** 2 bp, one HCBC	
**HE617184**	**V4-B (7):** 2 bp, one HCBC	
**KC155324**	**V4-B (7):** 2 bp, one HCBC	
**KC155323**	**V4-B (1):** 3 bp, one HCBC	
**HE586506**	**V9-F1 (7):** 1 bp, ambiguity	
**HE586508**	-	
**HE586549**	-	
**HE586548**	-	
**HE586530**	-	
**HE586529**	-	
**HE586528**	-	
**HE586547**	-	
**HE586546**	-	
**AY333646**	-	
**AB742451**	-	
**JQ315652**	-	
**HE586517**	**ITS2-A4 (1):** 2 bp (Helix I), 1 bp (Helix III)	
**HE586511**	**V9-A (1):** 1 bp, ambiguity, **ITS2-A4 (9):** 1 bp (Helix I), 1 bp (Helix III), ambiguities	
**HE586510**	**V9-A (3):** 2 bp, ambiguities, **ITS2-A4 (10):** 2 bp (Helix I), 2 bp (Helix III), ambiguities, one possible HCBC	
**GQ122371**	-	
**AY762603**	-	
**FR865679**	**V4-A2 (5):** 1 bp	
**AB488787**	-	
**HE586527**	-	
**AY328523**	-	
**AY293947**	-	
**AY293932**	**ITS2-A4 (1):** 2 bp (Helix I), 1 bp (Helix III)	
**HE586553**	**ITS2-A4 (2):** 3 bp (Helix I)	
**HE586552**	**ITS2-A4 (2):** 3 bp (Helix I)	
**AY293939**	**ITS2-A4 (2):** 3 bp (Helix I)	
**AY293948**	**ITS2-A4 (3):** 2 bp (Helix I)	
**AY293942**	**ITS2-A4 (3):** 2 bp (Helix I)	
**AY293946**	**ITS2-A4 (4):** 2 bp (Helix I), 2 bp (Helix III), one HCBC	
**AY293943**	**ITS2-A4 (5):** 2 bp (Helix I), 1 bp (Helix III), one HCBC	
**AY293938**	**ITS2-A4 (5):** 2 bp (Helix I), 1 bp (Helix III), one HCBC	
**AY293936**	**ITS2-A4 (5):** 2 bp (Helix I), 1 bp (Helix III), one HCBC	
**AY293935**	**ITS2-A4 (14):** 2 bp (Helix I), 1 bp (spacer Helices II-III), 1 bp (Helix III), one HCBC	
**AY293934**	**ITS2-A4 (15):** 2 bp (Helix I), 2 bp (Helix III), one HCBC	
**AY293940**	**ITS2-A4 (14):** 2 bp (Helix I), 1 bp (spacer Helices II-III), 2 bp (Helix III), one HCBC	
**HE586557**	**ITS2-A3 (6):** 3 bp (Helix I), 3 bp (Helix III), one error	
**AY333650**	**ITS2-A4 (7):** 4 bp (Helix I), 1 bp (Helix III)	
**AY293945**	**ITS2-A4 (8):** 1 bp (Helix I)	
**AY293944**	**ITS2-A4 (8):** 1 bp (Helix I)	
**AY293941**	**ITS2-A4 (8):** 1 bp (Helix I)	
**AY293937**	**ITS2-A4 (8):** 1 bp (Helix I)	
**AY293933**	**ITS2-A4 (8):** 1 bp (Helix I)	
**HE586554**	**ITS2-A4 (10):** 2 bp (Helix I), 2 bp (Helix III), ambiguities, one possible HCBC	

Table S7B: Meta data of the GenBank entries found by the BLAST search of the different barcode regions and its evaluation.

	region: no. base differences, evaluation	comment
**HE586544**	**ITS2-A4 (11):** 2 bp (Helix I), 2 bp (Helix III), ambiguities, one possible HCBC	
**HE586556**	**ITS2-A4 (12):** 1 bp (5.8S), ambiguity, 3 bp (Helix I)	
**HE586555**	**ITS2-A4 (12):** 1 bp (5.8S), ambiguity, 3 bp (Helix I)	
**HE586543**	**ITS2-A4 (13):** 3 bp (Helix I), 2 bp (Helix III), ambiguities, one possible HCBC	
**AB488788**	-	
**AB488795**	**V9-A (5):** 2 bp, errors	
**JX169832**	-	
**HE586519**	-	
**FR865588**	-	
**HQ287283**	**V4-G (19):** 1 bp, ambiguity	
**HE586507**	-	
**HE586509**	**V9-F1 (9):** 2 bp, errors	
**JQ717057**	-	
**JN573865**	-	
**HQ287296**	-	
**HQ287295**	-	
**HQ287294**	-	
**HQ287291**	-	
**HQ287290**	-	
**HQ287289**	-	
**HQ287288**	-	
**HQ287287**	-	
**HQ287286**	-	
**HQ287285**	-	
**HQ287284**	-	
**HQ287282**	-	
**HQ287281**	-	
**HQ287280**	-	
**HQ287278**	-	
**HQ287277**	-	
**HQ287276**	-	
**HQ287275**	-	
**HQ287274**	-	
**HQ287273**	-	
**HQ287272**	-	
**HQ287271**	-	
**HQ287270**	-	
**FJ648513**	-	
**AM981206**	-	
**EU127471**	-	
**EU127470**	-	
**AM167525**	-	
**AJ302939**	-	
**HQ287293**	**V4-G (16):** 1 bp	
**HQ287292**	**V4-G (16):** 1 bp	
**AB721029**	**V4-G (20):** 1 bp, HCBC	
**EU127472**	**V4-G (20):** 1 bp, error	

Table S7B: Meta data of the GenBank entries found by the BLAST search of the different barcode regions and its evaluation.

	region: no. base differences, evaluation	comment
**HE586523**	**ITS2-G1 (22):** 4 bp (Helix I)	
**HE586522**	-	
**DQ660909**	-	
**HE586536**	**V9-F1 (8):** 1 bp, error	
**HE586538**	**V9-F1 (9):** 2 bp, errors	
**HE586537**	-	
**HE586535**	-	
**HE586534**	**ITS2-G3 (23):** 1 bp (Helix I), error	
**HE586531**	**ITS2-G3 (24):** 1 bp (Helix III), error	
**HE586533**	**ITS2-G3 (25):** 3 bp (Helix I), ambiguity	
**HE586532**	**ITS2-G3 (26):** 3 bp (Helix I), one HCBC	**entry contains many ambiguities and errors, therefore consider as BC7a**
**HE586539**	**ITS2-G3 (27):** 3 bp (Helix I), one HCBC, 1 bp (Helix III) ambiguities	**entry contains many ambiguities and errors, therefore consider as BC7a**
**GQ487247**		
**GQ502290**		
**GQ502289**		
**FR865691**		
**FJ592475**		
